# Effects of Gamma Radiation on Single- and Multicomponent Organic Crystalline Materials

**DOI:** 10.1021/acs.cgd.2c01504

**Published:** 2023-03-02

**Authors:** Samantha J. Kruse, Leonard R. MacGillivray, Tori Z. Forbes

**Affiliations:** Department of Chemistry, University of Iowa, Chemistry Building, Iowa City, Iowa 52242, United States

## Abstract

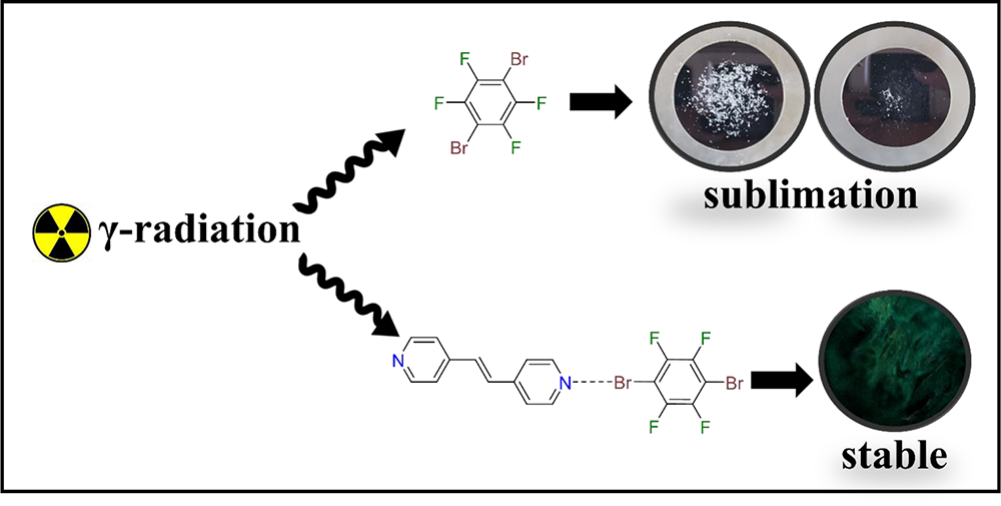

Exploration of highly ionizing radiation damage to organic materials has mainly been limited to polymers and single-component organic crystals due to their use in coatings and scintillation detection. Additional efforts are needed to create new tunable organic systems with stability in highly ionizing radiation to rationally design novel materials with controllable chemical and physical properties. Cocrystals are a promising class of compounds in this area because of the ability to rationally design bonding and molecular interactions that could lead to novel material properties. However, currently it is unclear if cocrystals exposed to radiation will maintain crystallinity, stability, and physical properties. Herein, we report the effects of γ radiation on both single-component- and multicrystalline organic materials. After irradiation with 11 kGy dose both single- (*trans*-stilbene, *trans*-1,2-bis(4-pyridyl)ethylene (**4,4′-bpe**), 1,*n*-diiodotetrafluorobenzene (**1,*n*-C_6_I_2_F_4_**), 1,*n*-dibromotetrafluorobenzene (**1,*n*-C_6_Br_2_F_4_**), 1,*n*-dihydroxybenzene (**1,*n*-C_6_H_6_O_2_**) (where *n* = 1, 2, or 3)), and multicomponent materials (**4,4′-bpe**)·(**1,*n*-C_6_I_2_F_4_**), (**4,4′-bpe**)·(**1,*n*-C_6_Br_2_F_4_**), and (**4,4′-bpe**)·(**1,*n*-C_6_H_6_O_2_**) were analyzed and compared to their preirradiated forms. Radiation damage was evaluated via single-crystal- and powder-X-ray diffraction, Raman spectroscopy, differential scanning calorimetry, and solid-state fluorimetry. Single-crystal X-ray diffraction analysis indicated minimal changes in the lattice postirradiation, but additional crystallinity changes for bulk materials were observed via powder X-ray diffraction. Overall, cocrystalline forms with **4,4′-bpe** were more stable than the related single-component systems and were related to the relative stability of the individual conformers to γ radiation. Fluorescence signals were maintained for *trans*-stilbene and **4,4′-bpe**, but quenching of the signal was observed for the cocrystalline forms to varying degrees. Three of the single components, 1,2-diiodotetrafluorobenzene (**1,2-C_6_I_2_F_4_**), 1,4-diiodotetrafluorobenzene (**1,4-C_6_I_2_F_4_**), and 1,4-dibromotetrafluorobenzene (**1,4-C_6_Br_2_F_4_**), also underwent sublimation within an hour of exposure to air postirradiation. Further analysis using differential scanning calorimetry (DSC) and Raman spectroscopy attributed this phenomenon to removal of impurities adsorbed to the crystal surface during irradiation.

## Abstract 2

Upon exposure to γ radiation, single-component aromatic organic small molecules cocrystallized with highly conjugated small molecules afford mitigation of structural defects.

## Introduction

Materials that display resistance to ionizing radiation by conserving structural integrity and their physical and chemical properties are vital to the development of sustainable solar cells, sensitive radiation detectors, nuclear forensics, aerospace materials, shielded nuclear reactors, and radiomedicine.^1−9^ In space science, cosmic rays deliver high-energy radiation to materials that can lead to significant structural degradation of these materials.^10−16^ Radiation damage is also a concern for the storage and handling of nuclear waste due to the presence of a suite of radionuclides that releases ionization radiation (α, β, and γ) that will degrade materials associated with containment and long-term monitoring.^17^ In radiomedicine, γ radiation is used for cancer diagnostics and therapies; thus, novel materials for both detection (scintillation) and delivery are important to the advancement of the field.^18^ Radiation can also cause secondary damage to electronic devices, when protective coatings are not able to sufficiently withstand the dose.^19^ Therefore, advancements in materials that can withstand high-radiation fields are crucial in many sectors and rely on a fundamental understanding of how radiation interacts with these materials to rationally design stable materials with large exposures.^20^

Previous work on solids typically focused on inorganic solid-state materials and polymers exposed to high ionizing radiation. Inorganic compounds are used as optical materials for coatings on solar cells and satellites (e.g., metal oxides, glasses) but can undergo physical changes with exposure to radiation, such as the formation of F-centers that lead to darkening and a reduction of light transmission.^10−16,21^ Inorganic materials tend to be more radiation-resistant than organic compounds but are also more expensive for practical applications and tend to be more brittle and sensitive to environmental conditions.^4,10−16,21−24^ Both plastics and organic crystalline materials (e.g., stilbene, anthracene) are widely used scintillators materials, but plastics outperform crystalline forms. For organic crystals, shifts in the band edge are observed at low doses, and heavy damage is observed at 21 kGy.^25−28^ Similarly, most organic polymers can withstand low (10–100 Gy) or moderate (1–10 kGy) doses without significant changes in mechanical properties (tensile strength, glass transition temperature) or radiation adsorption behavior. Even with the stability of the organic polymers under a moderate dose, there is still a concern about their overall durability when exposed to continuous irradiation, particularly in the presence of oxygen gas.^25−28^ Radiation-induced damage within polymer materials is related to the formation of radical species that causes chain scission reactions and cross-linking that changes the overall properties of the material.^29−31^ However, a study by Quaranta et al. on two-component polymeric materials demonstrated enhanced radiation resistance, suggesting that additional stability and tunability can be achieved in binary systems.^28^

Cocrystals have similarity to organic crystalline materials, but the presence of multiple molecules within the extended lattice enables control of composition and dimensionality in the solid state, with potential to offer similar tunability and radiation resistance as the two-component polymer systems. These multicomponent materials can be engineered to possess precise noncovalent forces such as hydrogen and/or halogen bonds to control of dimensionality, packing, and interactions that can lead to unique chemical and physical properties.^32−36^ In addition, cocrystals are arguably less costly, toxic, typically more abundant, and more mechanically flexible, than inorganic materials. While single-component organic crystalline materials are widely used as scintillation materials, the relative stability of multicomponent cocrystals to ionizing radiation has not been evaluated in the previously reported literature. Based upon the previous work by Quaranta et al., we hypothesized that cocrystals will be more radiation-resistant than single-component systems because of the rationally designed intermolecular interactions within the materials that can improve overall lattice stability.^28^

**Scheme 1:**
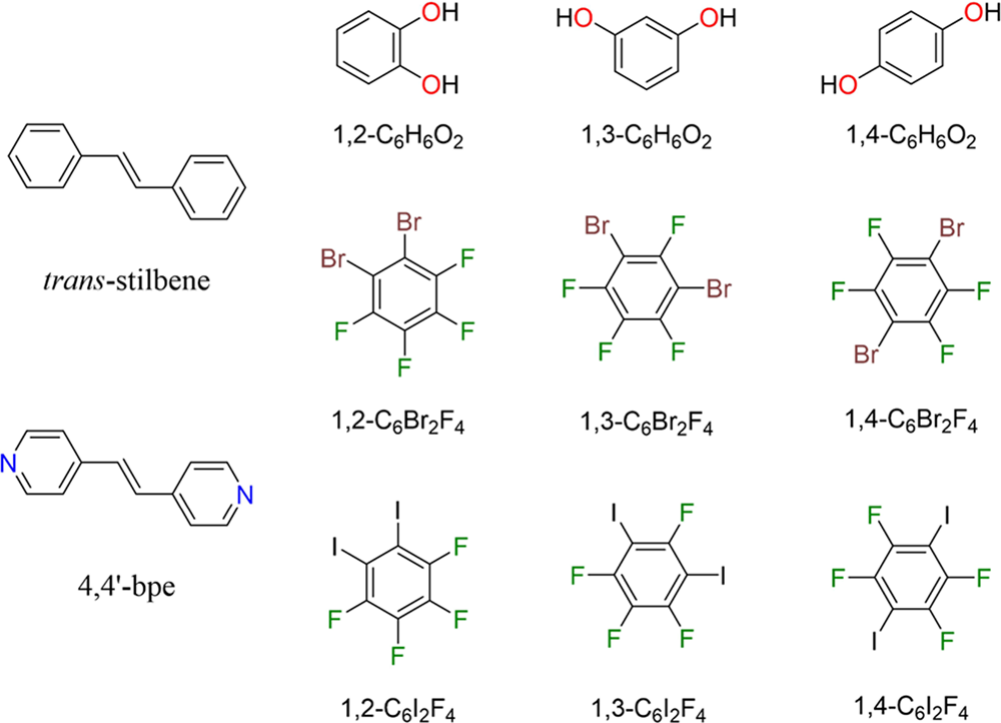
Wire Representation of *trans*-stilbene, Which Is a Common Single-Component Organic Crystal Utilized in Radiation Detection *trans*-Stilbene can be structurally compared to 1,2-bis(4-pyridyl)ethylene (**4,4′-bpe**), which can be combined with the functionalized benzene rings to form binary cocrystals.

In this study, we report the effects of ionizing γ radiation on the structural integrity on single-component materials: *trans*-stilbene, *trans*-1,2-bis(4-pyridyl)ethylene (**4,4′-bpe**), 1,*n*-diiodotetrafluorobenzene (**1,*n*-C_6_I_2_F_4_**), 1,*n*-dibromotetrafluorobenzene (**1,*n*-C_6_Br_2_F_4_**), and 1,*n*-dihydroxybenzene (**1,*n*-C_6_H_6_O_2_**) (where *n* = 1, 2, or 3), as well as the related binary cocrystals (**4,4′-bpe**)·(**1,*n*-C_6_I_2_F_4_**), (**4,4′-bpe**)·(**1,*n*-C_6_Br_2_F_4_**), and (**4,4′-bpe**)·(**1,*n*-C_6_H_6_O_2_**) (Scheme 1). A common theme of components is aromaticity, which is known for increased radiation resistance in organic materials. Aromaticity was viewed as most important since π-electrons reduce the probability of localization of excitation at a specific bond within the molecule, which therefore could provide fewer potential defects such as bond breakage.^37^ The bipyridine **4,4′-bpe** is also a common reactant used to construct cocrystals, and importantly for the current study it is a ‘derivative’ of a commonly used organic scintillator, namely, *trans*-stilbene. For the binary cocrystals, we evaluated how the incorporation of noncovalent interactions, such as halogen- (X-) and hydrogen- (H-) bonding interactions, within these systems provides rationally designed structural integrity with exposure to γ radiation. All these binary cocrystals form infinite 1-D arrays of C–X/H···N halogen/hydrogen bonds and crystallize in yields >95% and do not retain any solvent/mother liquor. Both the pure solid-state single components and the respective H- or X-bonded cocrystalline forms were characterized by single-crystal and powder X-ray diffraction before and after irradiation to evaluate crystallinity. Additional characterization via Raman and solid-state fluorescence spectroscopy and DSC was used to further explore changes in the physical properties of the single component and binary material.

## Experimental Section

### Synthesis

Synthesis of the cocrystals was conducted using similar methodologies to previous reports by using a 1:1 ratio of reactant: coformer (**4,4′-bpe**: **1,*n*-C_6_I_2_F_4_**, **1,*n*-C_6_Br_2_F_4_** or **1,*n*-C_6_H_6_O_2_**, where *n* = 1, 2, or 3). In each case, the reactant and coformer were weighed into separate 20 mL scintillation vials.^38^ For the formation of the halogen-bonded cocrystals, both the reactant (**4,4′-bpe**) and coformer (**1,*n*-C_6_I_2_F_4_** or **1,*n*-C_6_Br_2_F_4_**, where *n* = 1, 2, or 3) were dissolved in CHCl_3_ (2 mL each) and then mixed to form the final reaction solution. For the hydrogen-bonded cocrystals, the reactant was dissolved in CHCl_3_ (2 mL), and the coformer (**1,*n*-C_6_H_6_O_2_**, where *n* = 1, 2, or 3) was dissolved in a mixed solvent of EtOH and CHCl_3_ (1 mL each, respectively) and then mixed together to form the final solution. Vials were capped tightly to allow for slow evaporation affording high-quality single crystals for diffraction within 2 days and relative yields of approximately 95%. Samples were evaporated to complete dryness.

### γ-Irradiation

CAUTION: Cs-137 is a radioactive γ emitter. Irradiation experiments were carried out by trained personnel in a licensed research facility.

Single-component- and cocrystals were each added to 0.5 dram borosilicate glass vials. The vials were evacuated and backfilled with inert gas (Argon) and tightly capped before irradiation to prevent the presence of reactive O_2_ in the system. Samples were transported to the University of Iowa Free Radical and Radiation Facility for irradiation with a Cs-137 monoenergetic source (0.667 MeV). The γ irradiator has the ability to irradiate samples at a rate between 10 and 3200 cGy·min^–1^, and for this study, the samples were irradiated in their dram vials for 8.45 h to yield a total delivered dose of 11.00 kGy. Samples were safe to handle immediately after irradiation.

### Crystal Structure Determination

A high-quality single crystal of each compound pre and postirradiation was isolated on a MiTeGen micromount and mounted on a Bruker D8 Quest single-crystal diffractometer equipped with a microfocus X-ray beam (Mo Kα; λ = 0.71073 Å) and a CMOS detector. Frames were collected at 100 K (Oxford Systems low temperature cryosystem) with the Bruker APEX4 software package. Peak intensities were corrected for Lorentz, polarization, background, and absorption effects using the APEX4 software. Omega and phi scans were collected to provide full coverage of the diffraction space with high redundancy. Initial structure solution was determined by intrinsic phasing and refined on the basis of F^2^ for all unique data using the SHELXL version 5 program. H atoms were placed with a riding model for **4,4′-bpe** and **1,*n*-C_6_H_6_O_2_** molecules. Selected details on the structural refinement and selected bond distances and angles can be found in Tables S1–S17 in the Supporting Information (SI) section. The Bruker APEX4 software package was also used to obtain mosaicity values for each data set to compare crystallinity of each sample pre and postirradiation.

### Powder X-ray Diffraction

Both the single-component- and cocrystals were ground to a polycrystalline powder, and an internal standard of NaCl was added to the mixture. NaCl was chosen because the diffraction peaks did not interfere with any of the single- or multicomponents. Each sample contained 20 mg of material that was ground with 5 mg of NaCl for 5 min to form a fine powder and then sieved to create a homogeneous mixture. These samples were analyzed on a Bruker D-5000 powder X-ray diffractometer (Cu Kα = 1.54 Å) equipped with a LynxEye solid-state detector to determine the purity of the sample. Scans were performed from 5 to 60° 2θ with a step size of 0.05° 2θ and a count time of 1 s/step. Experimental patterns were compared pre and postirradiation for each sample.

### Raman Spectroscopy

Solid-state Raman spectroscopy was performed on both single-component and co-crystals. These samples were isolated and ground for 5 min to form a fine powder and pressed into a flat layer on a glass slide. Solid-state Raman spectra were acquired on the single-component and co-crystalline materials with a SnRI High-Resolution Sierra 2.0 Raman spectrometer equipped with 785 nm laser energy and a 2048 pixels TE-cooled CCD. Laser power was set to the maximum output value of 15 mW, giving the highest achievable spectral resolution of 2 cm^–1^. Each sample was irradiated for an integration time ranging from 0.25 to 2 s and automatically reiterated three times in a multiacquisition mode with the raster on. The average of the spectra acquired for a sample is reported as the final Raman spectrum. In order to accurately process the Raman signals observed, the background was subtracted using PreDICT 64-bit software, multiple peaks were fit using the peak analysis protocol with Lorentzian functions, and all the fitting parameters were converged in the OriginPro 9.60 (OriginLab, Northampton, MA) 64-bit software.

### Differential Scanning Calorimetry

A DSC Q100 (TA Instrument, USA) calorimeter heating from 50 to 140 °C at 5 °C·min^–1^ was used to assess sublimation properties of **1,4-C_6_Br_2_F_4_** pre and postirradiation. Calibration was carried out with an indium and sapphire standard, and an empty, hermetically sealed aluminum pan was used as a reference. Approximately 7 mg of **1,4-C_6_Br_2_F_4_** was weighed using a Toledo microbalance with 1 μg accuracy. The sample was placed in an aluminum pan, capped with an aluminum lid, and hermetically sealed. Data were analyzed using the free-to-use TRIOS version 5.1.1 software by TA Instruments.

### Solid-State Fluorometry

A CRAIC Microspectrometer solid-state UV–VIS–NIR equipped with a mercury lamp was used to collect fluorescence measurements on pre and postirradiated samples. Crystalline samples were placed onto glass slides and focused under the microscope. Measurements and figures were collected under a 10x objective and a set wavelength of 365 nm. Spectra were generated from 25 averaged scans of each sample, and spectra were averaged over three different crystals with an integration time ranging from 500 to 1500 s. Dark scans for background collection were taken between each sample.

## Results and Discussion

### Assessment of Crystallinity for Pre and Postirradiation Using Single-Crystal and Powder X-ray Diffraction

Pre and postirradiation samples were first evaluated via single-crystal X-ray diffraction to explore changes in the unit cell dimensions and the structural features of each material except for **1,3-C_6_I_2_F_4_**, **1,2-C_6_Br_2_F_4_**, and **1,3-C_6_Br_2_F_4_** since these single components are liquids at room temperature and could not be evaluated with this technique. Unit cell dimensions could not be reported for **1,2-C_6_I_2_F_4_**, **1,4-C_6_I_2_F_4_**, and **1,4-C_6_Br_2_F_4_** after exposure to γ radiation owing to sample stability issues that will be discussed vide infra. For each single-component and co-crystalline material, five fast scans were collected on five different crystals within the vial pre and postirradiation for averaged values of unit cell parameters (Table 1). Unit cell percent changes were calculated using percent change (eq 1) where *x*_1_ and *x*_2_ are the unit cell parameters pre and postirradiation, respectively.
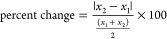
1

**Table 1:** Average Intensity Percent Decrease upon Exposure to γ Radiation for Each Single- and Multicomponent Crystalline Solid

coformers	single-component percent intensity change (%)	cocrystallized with **4,4′-bpe** percent intensity change (%)
**1,2-C_6_I_2_F_4_**	15.3	4.2
**1,3-C_6_I_2_F_4_**	liquid	50.7
**1,4-C_6_I_2_F_4_**	79.2	49.9
**1,2-C_6_Br_2_F_4_**	liquid	48.7
**1,3-C_6_Br_2_F_4_**	liquid	7.9
**1,4-C_6_Br_2_F_4_**	9.4	2.9
**1,2-C_6_H_6_O_2_**	19.9	18.8
**1,3-C_6_H_6_O_2_**	92.9	80.4
**1,4-C_6_H_6_O_2_**	36.3	4.6
*trans*-Stilhene	14.47	
**4,4′-bpe**	3.0	

**Figure 1:**
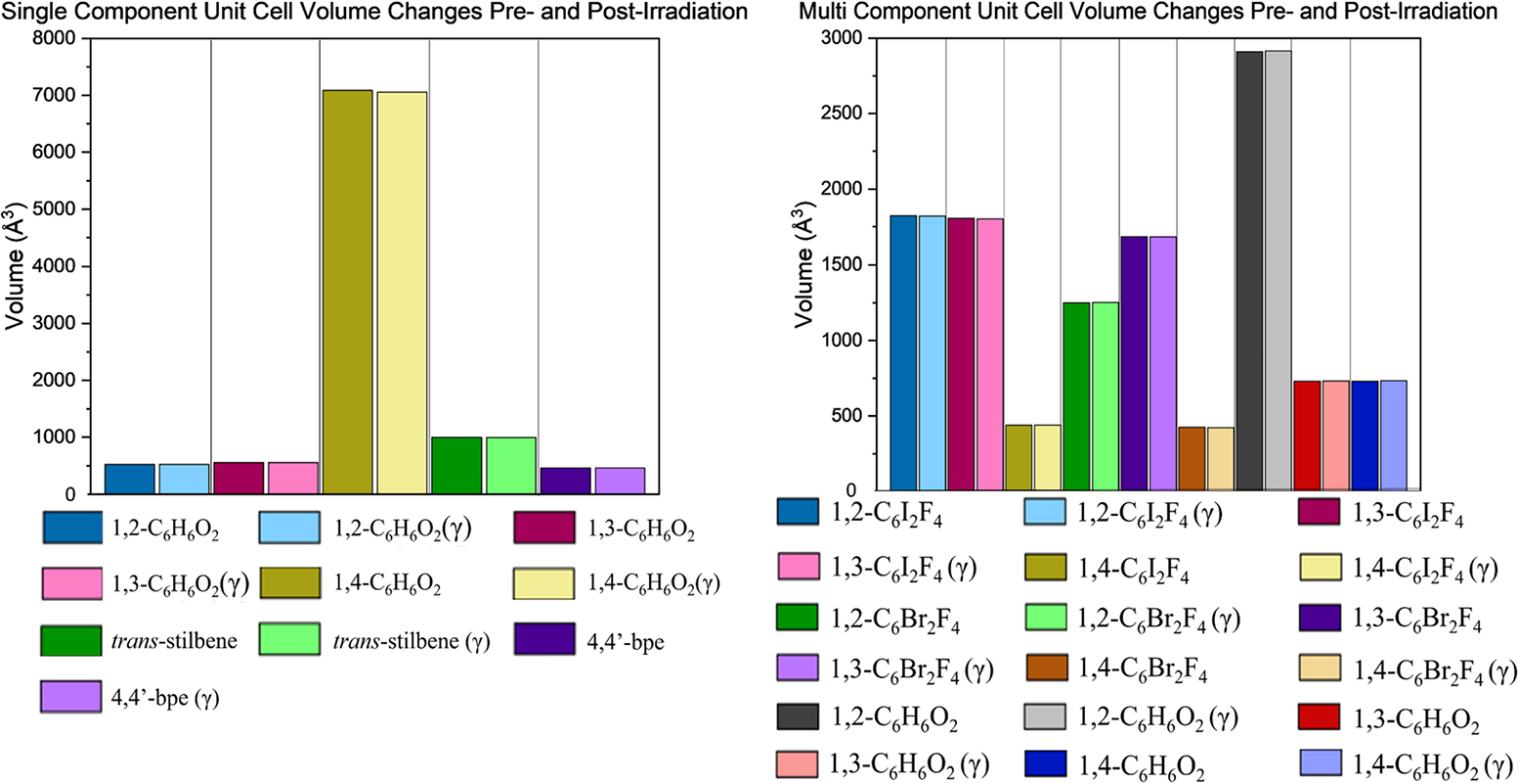
Bar graph of unit cell volume changes from radiation exposure for each single-component (left) and cocrystals formed with **4,4′-bpe** (right) crystals. Postirradiated samples are denoted with a lighter color respective to its nonirradiated form and (γ) in the legend. Error bars are too small to be seen.

Largest changes between samples from irradiation correspond to the volume of the unit cell, but the percent changes were still all below 1% (Figure 1). Given that the unit cells are relatively small, we determined that a change greater than 2% would be considered significant; therefore, no change significant deviations in the unit cell parameters were observed for these systems. Additional exploration of the structural features associated with the structural determination of these materials also did not yield any observable changes in the molecule or its overall packing. We note that there are also no observable differences in the unit cell parameters for the *trans*-stilbene material.

**Figure 2:**
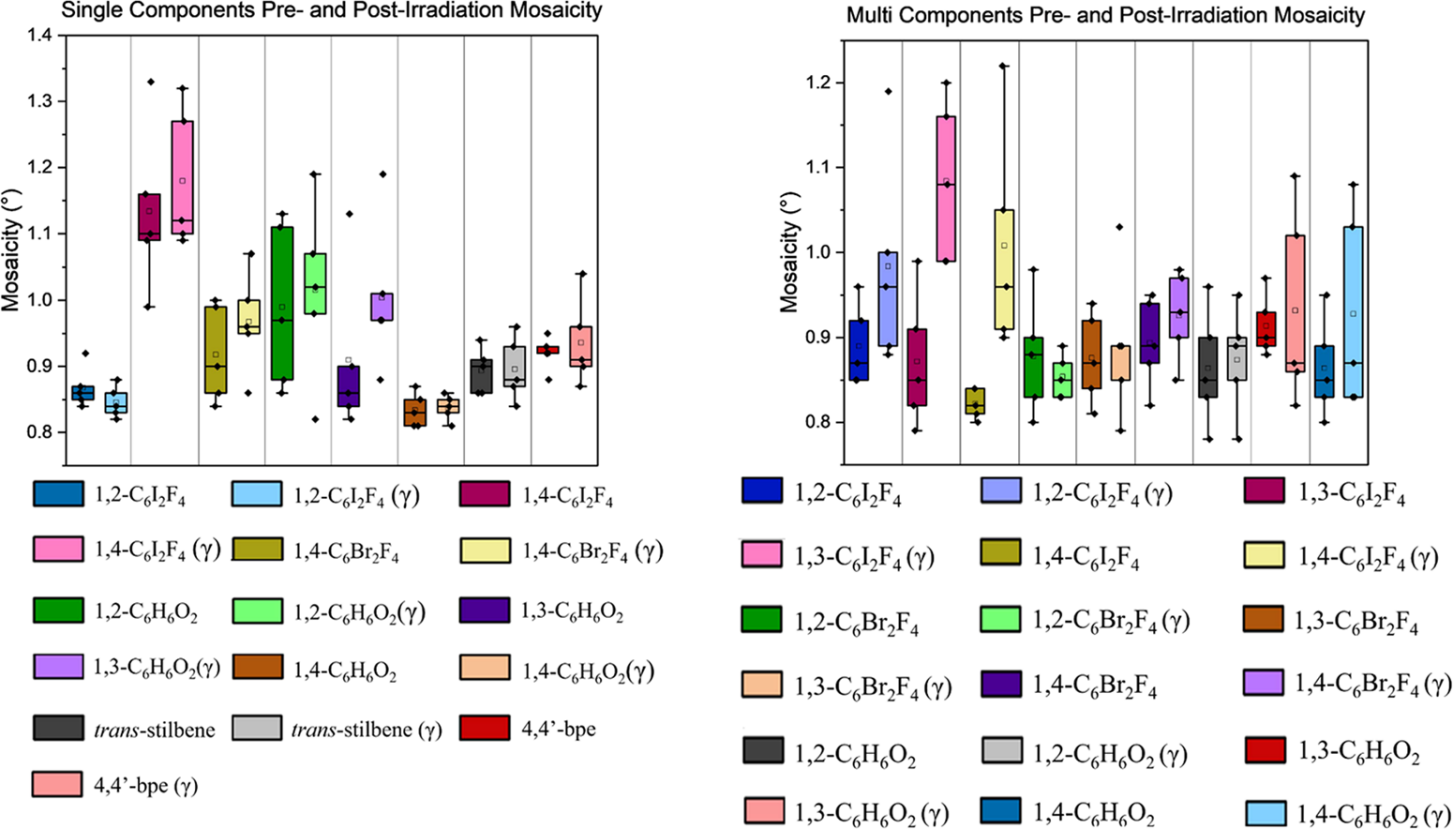
Box and whiskers graph of mosaicity values before and after radiation exposure. Single components are show in the graph on the left, cocrystals formed with **4,4′-bpe** are shown on the right. Postirradiated samples are denoted with a lighter color with respect to its nonirradiated form and (γ) in the legend.

Overall crystallinity was also assessed using mosaicity as a metric, which is a measure of spread of crystal plane orientation. Large changes in mosaicity can indicate either an increase or decrease in crystallinity with radiation exposure.^39^ Fast scans were collected for the single-component crystals postirradiation before complete sublimation could occur, and full scans were collected for the rest (Figure 2). Fast scans were collected on five different crystals for each sample, collecting a full 180° scan, and 200–250 reflections were harvested per collecting to obtain mosaicity values. Mosaicity values were averaged between five full data sets, and standard deviations were computed in Excel. Each sample has an observed increase in mosaicity, which would correspond to a slight decrease in crystallinity. However, the mosaicity values fall within range of their pre- or post-irradiated form; therefore, this increase in mosaicity was not found to be statistically significant.

**Figure 3:**
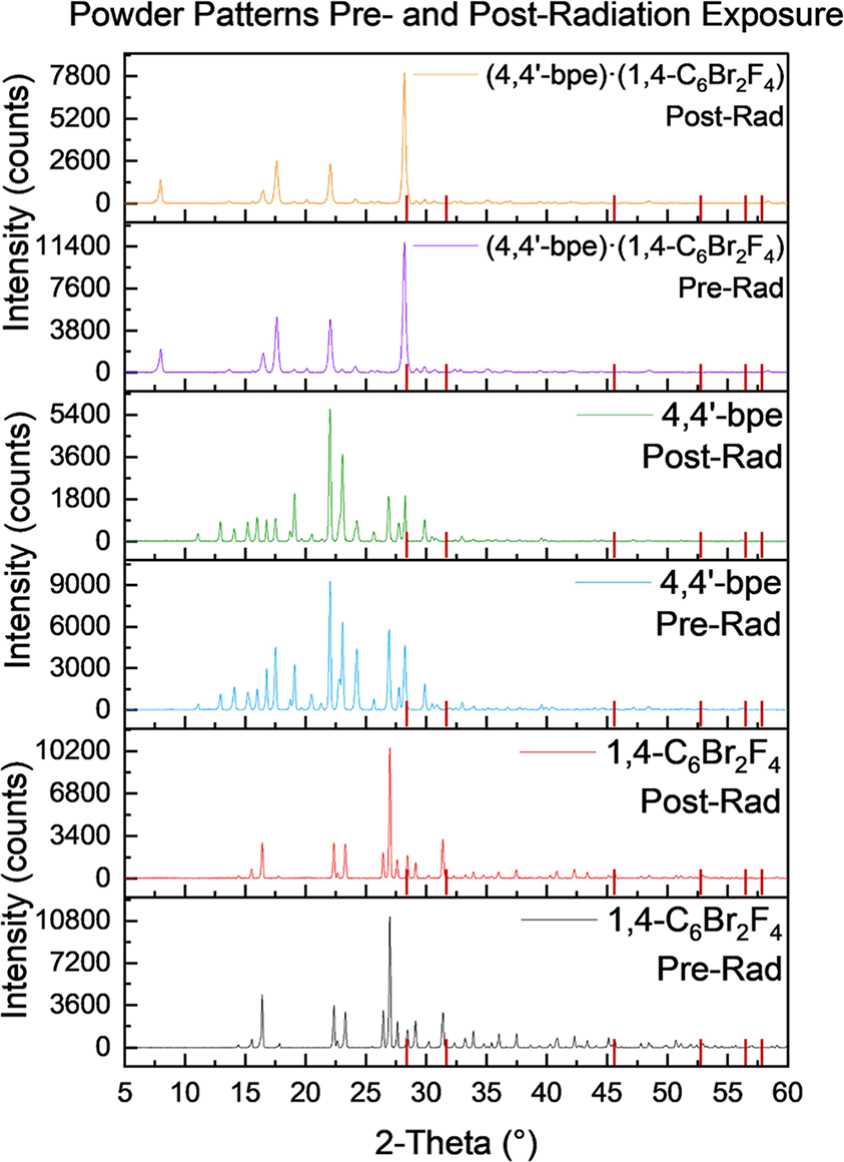
Normalized powder patterns of selected single-component- and binary cocrystals pre and postirradiation. Intensities have been normalized with a NaCl standard to provide semiquantitative analysis of the percent changes. Red lines on the x-axis represent the NaCl standard peak positions.

Analysis of single-crystal X-ray diffraction provides detailed information on select, highly crystalline particles; therefore, powder X-ray diffraction was used to evaluate the uniformity and bulk changes to the samples from radiation exposure. A NaCl standard was used to quantify any peak broadening or intensity changes that could potentially be observed between the pre and postirradiation samples. The most intense peak observed for NaCl is located at 31.79° 2Θ, whereas the major peaks associated with both the single-component- and co-crystalline samples reside between 10 and 30° 2Θ. An example of the powder X-ray diffraction results for the pure components (**4,4′bpe**; **1,4-C_6_Br_2_F_4_**) and co-crystalline form (**4,4′bpe**)·(**1,4-C_6_Br_2_F_4_**) is provided in Figure 3. The intensities were normalized using the generalized reference intensity ratio method described by Bish and Post and Snyder.^40^ Raw and processed diffractograms for all samples can be found in the Supporting Information section (Figures S1–S23).

Figure 3 demonstrates that the intensity of postirradiated samples is notably less than that in its preirradiated form, but there is no obvious phase change for the samples. In addition, the raw data (Figures S7–S23) indicated that the signal to noise for the samples changes postirradiation, but there is not significant evidence for the formation of an amorphous product. Minimal changes to the powder patterns are expected as the zero mass of γ radiation enables the ionizing radiation to travel farther distances than other forms^37^ but does not have a large amount of energy transfer. Therefore, the irradiation will not cause significant degradation of the crystalline lattice to form the amorphous material. This can be compared to α radiation, for example, where the ^4^He particle travels small distances (1–3 cm) because of the large mass and significant linear energy transfer that results in bond breakage.^41^ We also note that there was no observable widening of Braggs peaks postirradiation, which indicated that there was also not a significant decrease in the coherent domains of diffraction or particle size.

The relative intensity ratio method was utilized to provide a semiquantitative methodology to explore the differences in crystallinity. Averaged percent changes in intensity were calculated from the five most intensity peaks within the powder patterns to compare samples pre and postirradiation (Table 1). Changes in the single components ranged from a 3 to 93% decrease in intensities with single-component **1,4-C_6_I_2_F_2_** and **1,3-C_6_H_6_O_2_** showing the most degradation after irradiation. Of the single components, **4,4′bpe** performed the best with only a decrease of 3%, which was better than the change observed for the *trans*-stilbene standard where the decrease in crystallinity was 14%. Turning to the cocrystalline forms with **4,4′bpe**, we observed that in all cases there was a decrease in the overall degradation of the material. The stability of the cocrystals can be highlighted for (**4,4′bpe**)·(**1,4-C_6_I_2_F_4_**) where the decrease in crystallinity changed from 79 to 50% going from the single (**1,4-C_6_I_2_F_4_**) conformer to the multicomponent system. Similarly, degradation of the **1,4-C_6_H_6_CO_2_** was observed at 36% and then dropped to 4.6% with the formation of (**4,4′-bpe**)·(**1,2-C_6_H_6_O_2_**). An additional observation was noted for (**4,4′-bpe**)·(**1,2-C_6_H_6_O_2_**), where the preradiation form contained fairly poor signal to noise that improved upon irradiation (Figure S6). Previous reports suggest that the γ radiation either removes the amorphous content, or induces crystallization, thus, removing any prominent background features which could be associated with amorphous materials.^42,43^

While there are limitations to the relative intensity ratio methodology, it does provide some additional insights into the system. The biggest challenge for the methodology is that it requires careful sample preparation to create a homogeneous mixture, but additional problems (i.e., extinction, microabsorption, and preferred orientation of the sample) create additional sources of error.^43^ In this study, the sample was carefully prepared to create a homogeneous mixture, and we do not expect significant errors due to extinction or microabsorption because we are making comparisons to identical materials pre and postradiation. While there is no clear evidence of preferred orientation, there is evidence that the irradiation is impacting specific lattice planes by differing amounts (Tables S15–S31). An example of this can be noted in **(4,4′-bpe)**·**(1,3-C_6_Br_2_F_4_),** where three of the most intense peaks in the diffractogram do not change between pre and postradiation, whereas the other two decrease by 28 or 46%. This is contrasted with (**4,4′-bpe**)·(**1,2-C_6_Br_2_F_4_**), where the percent change between the five most intense peaks decreases by a narrower amount (43–54%). Comparing the (**4,4′-bpe**)·(**1,*n***-**C_6_Br_2_F_4_**), where *n* = 2, 3, or 4, cocrystals to each other structurally, the powder pattern with the fewest changes was associated with **1,4-C_6_Br_2_F_4_** where packing involved infinite 1-D linear assemblies compared to the other two which had discrete assemblies. Variations in crystal packing that result in differences in bond strengths between the lattice planes will be explored in greater detail in future studies.

Several other studies have utilized powder X-ray diffraction techniques to evaluate γ irradiation stability but are challenging to compare to the current study. There is limited analysis of organic crystals using this technique and so it is difficult to make additional comparisons between these systems. Other irradiation studies for materials do not provide information on the exact change in the intensities of the samples, providing qualitative analysis of peak positions and arbitrary values for intensities.^44^ The study by Hossain et al. provided detailed X-ray diffraction data for hydroxyapatite samples that underwent gamma irradiation but utilized parameters such as the crystallinity index and degree of crystallinity that are specific to the identity of the solid.^45^ Therefore, additional studies are warranted to provide a more consistent methodology to evaluate changes in crystallinity for irradiated materials.

The single- and multicomponent materials were also analyzed by Raman spectroscopy (Figures S24–S51). Each of the spectra was peak fit, and then the vibrational modes were assigned using previously published literature.^46−55^ Overall, the features within the Raman spectra for all the single- and multicomponent phases could be matched to the previously reported vibrational bands. There were variations in the intensities between the pre and postirradiation materials but because there was no internal spectroscopic standard in these samples there is no significance to these differences.

### Evaluating Physical Properties via Solid-State Fluorimetry

**Figure 4:**
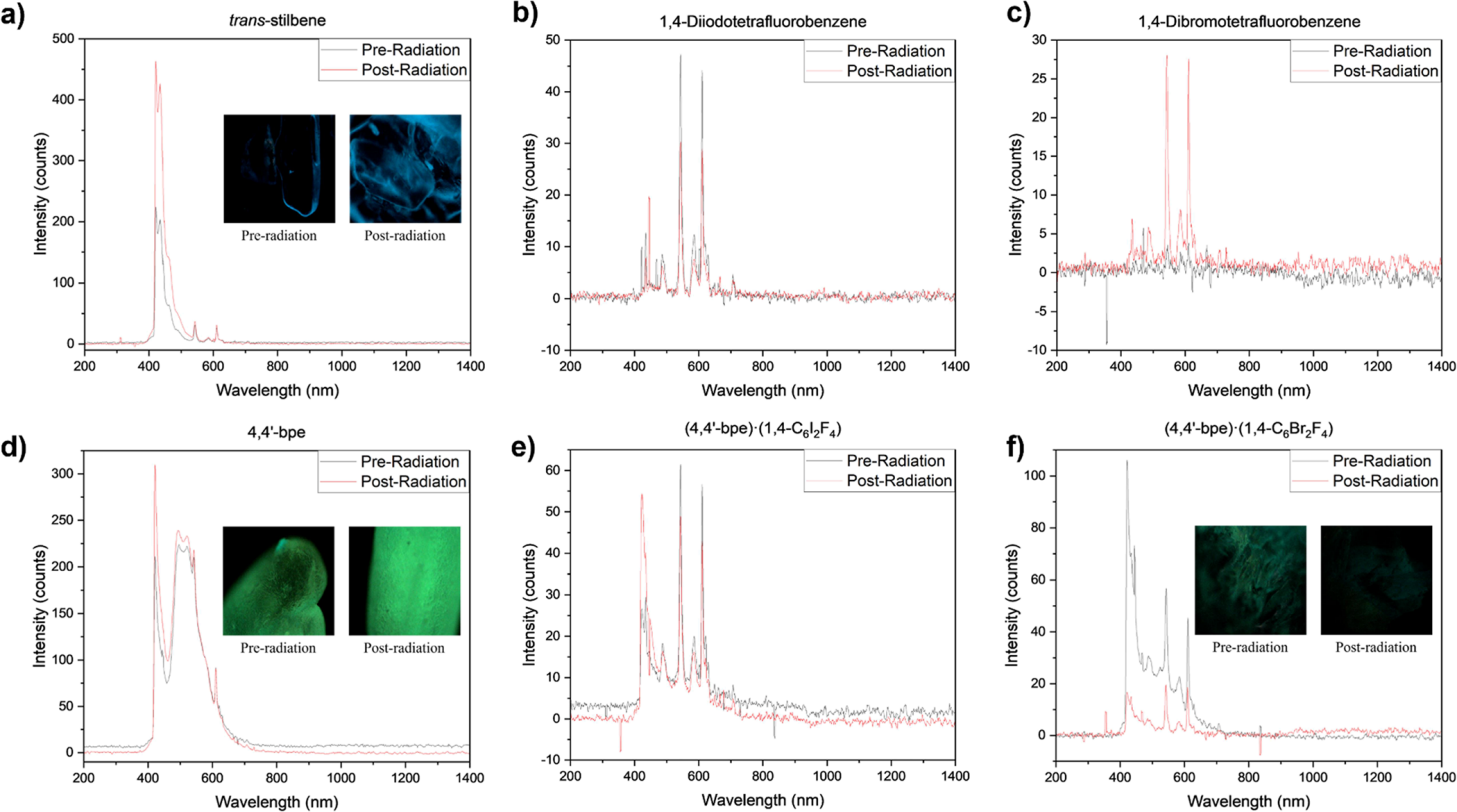
Solid-state fluorescence spectra of (a) *trans*-stilbene, (b) **1,4-C_6_I_2_F_4_**, (c) **1,4-C_6_Br_2_F_4_**, (d) **4,4′-bpe**, (e) (**4,4′-bpe**)·(**1,4-C_6_I_2_F_4_**), and (f) (**4,4′-bpe**)·(**1,4-C_6_Br_2_F_4_**) pre and postirradiation depicted as black and red, respectively. Images in (a), (d), and (e) include single crystals under a microscope at 365 nm wavelength and 10× objective before and after radiation exposure.

**Figure 5:**
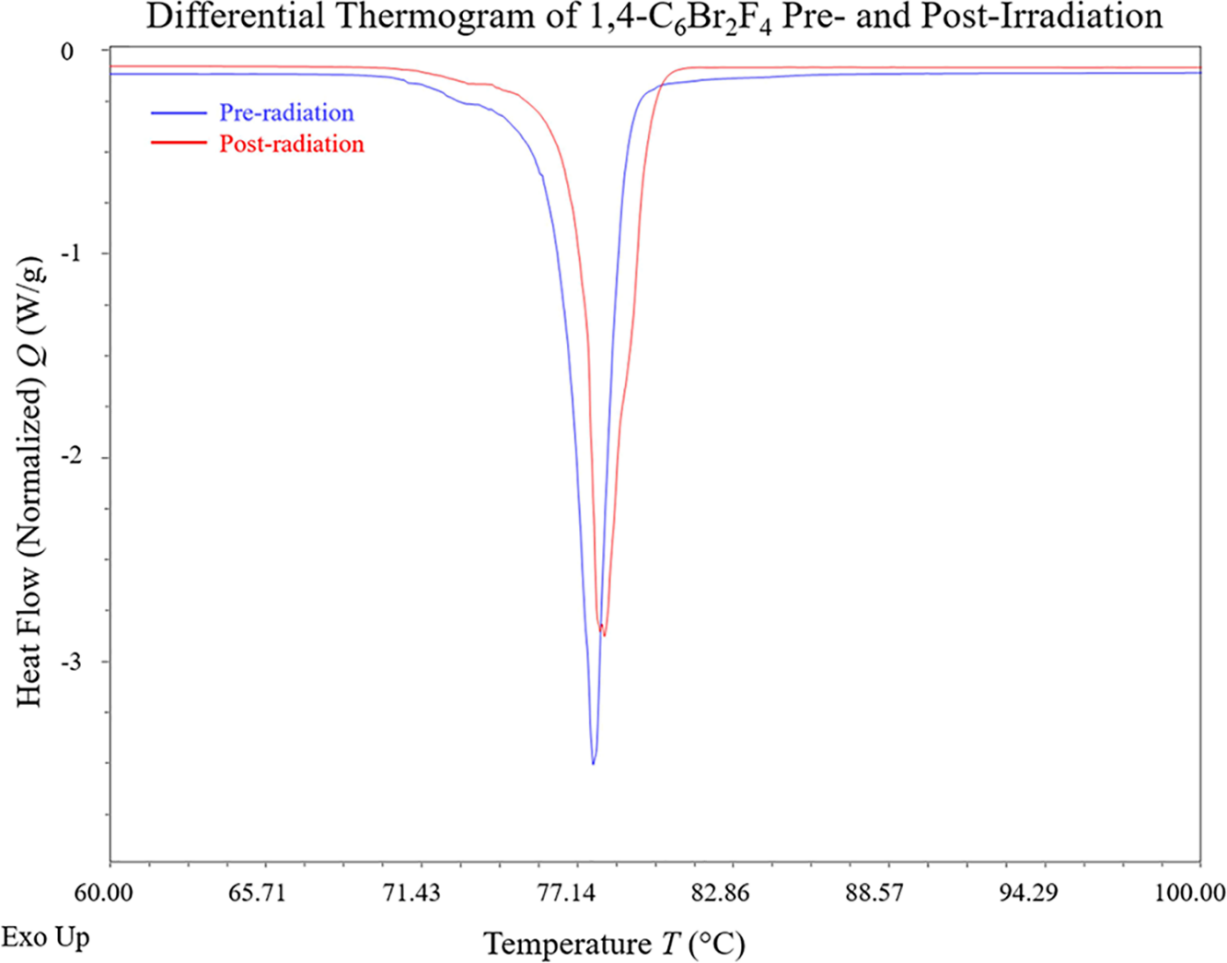
Differential thermograms overlaid for **1,4-C_6_Br_2_F_4_** before (blue) and after (red) 11 kGy of radiation exposure. Exothermic transitions are up, and endothermic transitions are down.

Given the importance of fluorescence in the use of organic crystals for scintillation devices, we also explored the fluorescence signal associated with the single- and multicomponent systems and compared to *trans*-stilbene as a reference. Single-component *trans*-stilbene and **4,4′-bpe** have inherent fluorescent properties upon excitation 365 nm due to high conjugation within these respective molecules, and this is clearly depicted in Figure 5. The single components with a single aromatic ring showed no or weak fluorescence both pre and postradiation (Figures S52–S57). This is expected as these single-component phases are known for being relatively weak fluorophores due to aggregating-induced quenching within the solid state.^56^ Turning to the multicomponent phases, we note differing levels of quenching of the fluorescence signal for the preradiation samples. This has been previously noted to occur by Tamuly et al. where they indicated **1,3-C_6_H_6_CO_2_** and **1,4-C_6_H_6_CO_2_** will engage in hydrogen bonding interactions with nitrogen-containing fluorophores to induce quenching in solution.^57^ Brahma et al. also noted that **1,3-C_6_H_6_CO_2_** caused quenching of phenazine in the solid state caused by parallel cofacial π-stacks among the phenazine molecules.^58^ A similar decrease in the fluorescence signal is noted for all multicomponent samples containing **1,*n*-C_6_H_6_CO_2_**. The cocrystals (**4,4′-bpe**)·(**1,4-C_6_Br_2_F_4_**) and (**4,4′-bpe**)·(**1,4-C_6_I_2_F_4_**) retained the largest amount of fluorescence compared to the **4,4′-bpe** single phase for the preirradiation materials (Figure 4). This agrees well with previously reported synthesis and characterization of halogenated benzene cocrystals reported by Gao et al. and Li et al. where fluorescence was also observed in these solid-state materials.^59,60^

Upon irradiation, the fluorescence is maintained for both single-component systems but is more variable for the multicomponent phases. Very little differences were noted in the fluorescence of the *trans*-stilbene and **4,4′-bpe** materials, but there was more variability for the other coformers. No fluorescent signal was observed for the initial **1,4-C_6_Br_2_F_4_** and **1,4-C_6_H_6_O_2_** materials, but after irradiation, a weak signal was detected. In the postirradiation multicomponent materials, the signal decreased substantially for the (**4,4′-bpe**)·(**1,4-C_6_Br_2_F_4_**), while the weaker fluorescing system of (**4,4′-bpe**)·(**1,4-C_6_I_2_F_4_**) had a smaller decrease postirradiation. It is interesting to note that the decrease in the crystallinity of the (**4,4′-bpe**)·(**1,4-C_6_Br_2_F_4_**) system is quite small (3%) compared to that observed for (**4,4′-bpe**)·(**1,4-C_6_I_2_F_4_**) 51%) because an increase in disorder is expected to decrease in the intensity of fluorescence in solid materials.^61,62^ For inorganic materials, NaCl, there is an increase in fluorescence upon exposure to gamma radiation due to the formation of an F-center (a free electron trapped within the lattice of the crystal).^63^ In other cases, an increase in disorder within the lattice resulted in a decrease in the intensity of fluorescence in solid materials.^61,62^ Additional experiments are ongoing to mechanistically explore the changes that occur in multicomponent systems upon irradiation and the impacts on their fluorescence properties.

### Case Study in Stability: Changes in Sublimation for **1,4-C_6_Br_2_F_4_** Pre and Postirradiation

As mentioned vide supra, the **1,4-C_6_Br_2_F_4_** postirradiation could not be compared to the as-synthesized materials because of stability issues, and we decided this warranted further analysis. More specifically, when postirradiation single crystals of **1,4-C_6_Br_2_F_4_** were placed on the X-ray diffractometer, the material sublimed within 40 min at 298 K under standard conditions. Data collection was also attempted with the sample cooled to 100 K under a N_2_ cryostream, but sublimation still occurred under these conditions. While this single component is under the category of perfluorinated carbons and is known to undergo sublimation,^38^ this phenomenon typically occurs at room temperature or elevated temperatures over an extended period of time (typically days). Similarly, **1,2-C_6_I_4_F_4_** and **1,4-C_6_I_4_F_4_** were stable enough for data collection preradiation, but also sublimed within an hour postradiation, preventing us from obtaining a complete data set for both single-crystal and powder X-ray diffraction.

As sublimation is phase change event, additional thermodynamic analysis for **1,4-C_6_Br_2_F_4_** for both pre and postirradiated forms was performed using DSC (Figure 5). In both cases, there is an endothermic transition that occurs at 77.72 and 78.12 °C for the pre and postradiation samples, respectively. Averaged triplicate data collected from DSC provide Δ*H*_sub_ = −24.568(4) and −21.581(8) kJ/mol for pre and postradiation samples, respectively. This is a total of 2.987 kJ/mol more exothermic for the irradiated sample, which is consistent with the observation that the postirradiation sample sublimed more readily than the initial sample.

**Figure 6:**
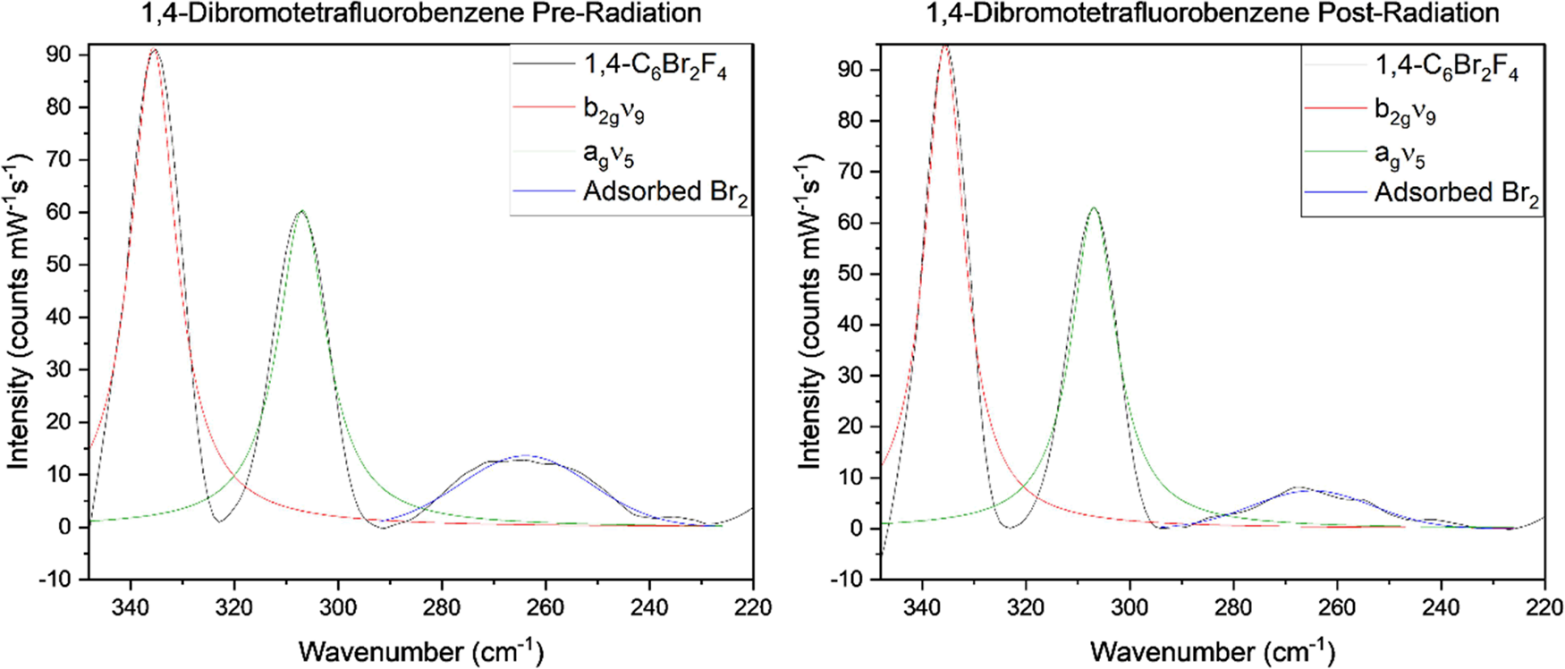
Spectral window of interest for the Br_2(g)_ adsorption feature in the solid-state Raman spectra of pre (left) and postirradiated (right) **1,4-C_6_Br_2_F_4_**.^48,54,55^

Further analysis of the **1,4-C_6_Br_2_F_4_** by Raman spectroscopy revealed a possible explanation for the change in stability for pre and postirradiation materials. Full Raman spectra and assigned vibrational modes are provided in the Supporting Information section (Figures S24–S51), but weak features in the spectral window between 225 and 350 cm^–1^ are highlighted in Figure 6. Notably for preirradiation of **1,4-C_6_Br_2_F_4_**, there is a band present at 275 cm^–1^ that does not correspond to the reported vibrational features for this molecule, and this feature decreases in intensity with irradiation by γ rays. We hypothesized that this band was an impurity that was adsorbed onto the surface of the **1,4-C_6_Br_2_F_4_** phase. More so, the synthesis of **1,4-C_6_Br_2_F_4_** reports the use of excess Br_2(g)_ and could have likely physiosorbed onto the surface of the product.^54,55^ This is supported by other studies which report that the Raman spectral features of physiosorbed Br_2_ occur between 270 and 300 cm^–1^, where the exact peak position was dependent on the identity of the material substrate.^54^ Previous work on the adsorption of Br_2_ onto Si crystals suggests that the overall energy of the surface adsorption is favorable, suggesting that this is likely to occur within the **1,4-C_6_Br_2_F_4_**.^55^ The decrease in the spectral feature for the irradiated samples suggest the removal of some of the adsorbed Br_2_. It is possible that this explains the difference in the sublimation behavior of the material as the Br_2_ may passivate the surface and slow the sublimation process. Removal of a certain amount of Br_2_ upon irradiation may enhance this behavior and results in the enthalpic change associated with desorption of Br_2_. Interestingly though, this was not observed the Raman spectra for either **1,2-C_6_I_2_F_4_** or **1,4-C_6_I_2_F_4_**.

## Conclusions

Herein, we have reported the effects of gamma radiation on 17 single-component and cocrystalline organic materials through techniques such as single- and powder-X-ray diffraction, DSC, Raman spectroscopy, and solid-state fluorometry. While little differences were noted in the single-crystal X-ray diffraction data, powder X-ray diffraction depicts decreases in peak intensity related to lowering of the crystallinity of the sample. This work demonstrates that selecting individual crystals is not adequate to understand overall changes in the bulk material and that powder X-ray diffraction provides a better picture of the impacts of radiation. Overall, the data presented here support the hypothesis that cocrystals have the ability to mitigate structural defects when exposed to γ radiation, particularly when one of the coformers (**4,4′bpe**) shows specific radiation resistance. Solid-state fluorimetry studies also demonstrate that the fluorescent behavior is variable with exposure to gamma irradiation and is likely dependent on the properties of the coformer, crystalline packing, and impacts of lattice defects or free electrons within the material. In addition, the perfluorinated single components **1,2-C_6_I_2_F_4_**, **1,4-C_6_I_2_F_4_** and **1,4-C_6_Br_2_F_4_** which are known to sublime over an extended period, had an enhancement of the physical property through radiation exposure. Evidence provided sublimation occurred more quickly due to impurities adsorbed to the original sample and their removal through irradiation.

This study clearly demonstrates that organic cocrystalline materials may be competitive alternatives to current organic scintillators and provide new opportunities for tunability through rational design. However, appropriate consideration of components to be formed into a cocrystal must be considered such as intermolecular interactions present and dimensionality for radiation-resistant applications. Increasing the amount of aromaticity within the multicomponent material should increase both the radiation resistance and the fluorescent signal, but the influence of π-stacking and hydrogen bonding within the crystalline lattice must be further evaluated to understand its impact on the enhancement or quenching of the signal. Finally, future studies evaluating the formation of free electrons from the irradiation process would provide additional insights into the solid-state reactivity that further guide our understanding of the process and possible design principles for the development of new materials for radiation resistance and detection.

## Supporting Information Available

Crystallographic information, vibrational spectroscopy, luminescence data of all seventeen materials investigated in this study, processed and raw powder X-ray diffraction patterns of each material, and experimental differential scanning calorimetric thermograms of perfluorinated aromatic rings pre- and post-irradiation, as well as a visual of the sublimation of **1,4-C_6_Br_2_F_4_** (PDF)

## References

[ref1] Papez N.; Gajdos A.; Sobola D.; Dallaev R.; Macku R.; Skarvada P.; Grmela L. Effect of gamma radiation on properties and performance of GaAS based solar cells. Appl. Surf. Sci. 2020, 527, 146766 10.1016/j.apsusc.2020.146766.

[ref2] Nikolic D.; Stankovic K.; Timotijevic L.; Rajovic Z.; Vujisic M. Comparative Studies of Gamma Radiation Effects on Solar Cells, Photodiodes, and Phototransistors. Int. J. Photoenergy 2013, 2013, 843174 10.1155/2013/843174.

[ref3] Hamada M. M.; Rela P. R.; da Costa F. E.; de Mesquita C. H. Radiation damage studies on the optical and mechanical properties of plastic scintillators. Nucl. Instrum. Methods Phys. Res. A 1999, 422, 148–154. 10.1016/S0168-9002(98)01091-2.

[ref4] Yang F.; Zhang L.; Zhu R. Gamma-Ray Induced Radiation Damage Up to 340 Mrad in Various Scintillation Crystals. IEEE Trans. Nucl. Sci. 2016, 63, 612–619. 10.1109/TNS.2015.2505721.

[ref5] Stecker F. W. Cosmic Gamma Rays; National Aeronautics and Space Administration, 1971.

[ref6] Adrovic F. Gamma Radiation; InTech, 2012.

[ref7] Ojova M. J.; Batyukhnova O. G. Glasses for Nuclear Waste Immobilization. In Waste Management Conference; Tuscon, AZ, 2007.

[ref8] McCloy J. S.; Goel A. Glass-ceramics for nuclear-waste immobilization. MRS Bull. 2017, 42, 233–240. 10.1557/mrs.2017.8.

[ref9] Mattsson S. Introduction: The Importance of Radiation Protection in Nuclear Medicine. In Radiation Protection in Nuclear Medicine; Springer: Berlin, Heidelberg, 2013; pp. 1 −3.

[ref10] Kamiya K.; Ozasa K.; Akiba S.; Niwa O.; Kodama K.; Takamura N.; Zaharieva E. K.; Kimura Y.; Wakeford R. Long-term effects of radiation exposure on health. Lancet 2015, 386, 469–478. 10.1016/S0140-6736(15)61167-9. 26251392

[ref11] Yoshikawa M.; Itoh H.; Morita Y.; Nashiyama I.; Misawa S.; Okumura H.; Yoshida S. Effects of gamma-ray irradiation on cubic silicon carbide metal-oxide-semiconductor structure. J. Appl. Phys. 1991, 70, 1309–1312. 10.1063/1.349587.

[ref12] Wong M. H.; Takeyama A.; Makino T.; Ohshima T.; Sasaki K.; Kuramata A.; Tamakoshi S.; Higashiwaki M. Radiation hardness of β-Ga_2_O_3_ metal-oxide-semiconductor field-effect transistors again gamma-ray irradiation. Appl. Phys. Lett. 2018, 112, 023503 10.1063/1.5017810.

[ref13] Arshak K.; Korostynska O. Response of metal oxide thin film structures to radiation. Mater. Sci. Eng. B 2006, 133, 1–7. 10.1016/j.mseb.2006.06.012.

[ref14] Kreidl N. J.; Hensler J. Formation of Color Center in Glass Exposed to Gamma Radiation. J. Am. Ceram. Soc. 2006, 38, 423–432. 10.1111/j.1151-2916.1955.tb14568.x.

[ref15] Wang T. T.; Zhang X. Y.; Sun M.; Du X. γ-Irradiation effects in borosilicate glass studied by EPR and UV-Vis spectroscopies. Nucl. Instrum. Methods Phys. Res. B 2020, 464, 106–110. 10.1016/j.nimb.2019.12.007.

[ref16] Bourezgui A.; Kacem I.; Daoudi M.; Al-Hossainy A. F. Influence of Gamma-Irradiation on Structural, Optical and Photocatalytic Performance of TiO_2_ Nanoparticles Under Controlled Atmospheres. J. Electron. Mater. 2020, 49, 1904–1921. 10.1007/s11664-019-07887-z.

[ref17] Zinkle S. J.; Was G. S. Materials challenges in nuclear energy. Acta Mater. 2013, 61, 735–758. 10.1016/j.actamat.2012.11.004.

[ref18] University of Rochester Medical Center Radiation Therapy and Cancer Treatment. https://www.urmc.rochester.edu/encyclopedia/content.aspx?ContentTypeID=85&ContentID=p00583 (accessed 2022-10-06).

[ref19] Isherwood L. H.; Athwal G.; Spencer B. F.; Casiraghi C.; Baidak A. Gamma Radiation-Induced Oxidation, Doping, and Etching of Two-Dimensional MoS_2_ Crystals. J. Phys. Chem. Commun. 2021, 125, 4211–4222. 10.1021/acs.jpcc.0c10095. PMC8025684 33841606

[ref20] Turhan M. F.; Akman F.; Taser A.; Dilsiz K.; Ogul H.; Kacal M. R.; Agar O. Gamma radiation hielding performance of Cu_x_Ag_(1-x)_-alloys: Experimental, theoretical and simulation results. Prog. Nucl. Energy 2022, 143, 104036 10.1016/j.pnucene.2021.104036.

[ref21] Kumar S.; Mann K. S.; Singh T.; Singh S. Investigations on the gamma-ray shielding performance of green concrete using theoretical, experimental and simulation techniques. Prog. Nucl. Energy 2021, 134, 103654 10.1016/j.pnucene.2021.103654.

[ref22] Volkringer C.; Falaise C.; Devaux P.; Giovine R.; Stevenson V.; Pourpoint F.; Lafon O.; Osmond M.; Jeanjacques C.; Marcillaud B.; Sabroux J. C.; Loiseau T. Stability of metal-organic frameworks under gamma irradiation. Chem. Commun. 2016, 52, 12502–12505. 10.1039/C6CC06878B. 27722563

[ref23] Ma C.; Liu H.; Wolterbeek H. T.; Denkova A. G.; Crespo P. S. Effects of High Gamma Doses on the Structural Stability of Metal-Organic Frameworks. Langmuir 2022, 38, 8928 10.1021/acs.langmuir.2c01074. 35816708 PMC9330767

[ref24] Volkwinger C.; Falaise C.; Devaux P.; Giovine R.; Stevenson V.; Pourpoint F.; Lafon O.; Osmond M.; Jeanjacques C.; Marcillaud B.; Sabroux J. C.; Loiseau T. Stability of metal-organic frameworks under gamma irradiation. Chem. Commun. 2016, 52, 12502–12505. 10.1039/C6CC06878B. 27722563

[ref25] Nambiar S.; Yeow J. T. W. Polymer-Composite Materials for Radiation Protection. Appl. Mater. Interfaces 2012, 4, 5717–5726. 10.1021/am300783d. 23009182

[ref26] Fraboni B.; Fraleoni-Morgera A.; Zaitsenva N. Ionizing Radiation Detectors Based on Solution-Grown Organic Single Crystals. Adv. Funct. Mater. 2016, 26, 2276–2291. 10.1002/adfm.201502669.

[ref27] Baccaro S.; Cemmi A.; Di Sarcina I.; Esposito B.; Ferrara G.; Grossi A.; Montecchi M.; Podda S.; Pompii F.; Quinteiera L.; Riva M. Radiation Damage Tests on Diamond and Scintillation Detector Components for the ITER Radial Neutron Camera. IEEE Trans. Nucl. Sci. 2018, 65, 2046–2053. 10.1109/TNS.2018.2807841.

[ref28] Quaranta A.; Carturan S.; Marchi T.; Antonaic A.; Kravchuk V. L.; Degerlier M.; Gramegna F.; Maggioni G. Radition hardness of polysiloxane scintillators analyzed by ion beam induced luminescence. Nucl. Instrum. Methods Phys. Res. B 2010, 268, 3155–3159. 10.1016/j.nimb.2010.05.077.

[ref29] Siddhartha; Aarya S.; Dev K.; Raghuvanshi S. K.; Krishna J. B. M.; Wahab M. A. Effects of gamma radiation on the structural and optical properties of Polyethyleneterephthalate (PET) polymer. Radiat. Phys. Chem. 2012, 81, 458–462. 10.1016/j.radphyschem.2011.12.023.

[ref30] Jeong J. O.; Park J. S.; Kim Y. A.; Yang S. J.; Jeong S. I.; Lee J. Y.; Lim Y. M. Gamma Ray-Induced Polymerization and Cross-Linking for Optimization of PPy/PVP Hydrogel as Biomaterial. Polymer 2020, 12, 111 10.3390/polym12010111. PMC7023038 31948023

[ref31] Demeter M.; Virgolici M.; Vancea C.; Scarisoreanu A.; Kaya M. G. A.; Meltzer V. Network structure studies on γ-irradiated collagen-PVP superabsorbent hydrogels. Radiat. Phys. Chem. 2017, 131, 51–59. 10.1016/j.radphyschem.2016.09.029.

[ref32] Zaitseva N.; Carman L.; Glenn A.; Hatarik R.; Hamel S.; Rupert B.; Faust M.; Schabes B.; Cherepy N.; Payne S. Pulse Shape Discrimination in Impure and Mixed Single-Crystal Organic Scintillators. IEEE Trans. Nucl. Sci. 2011, 58, 3411–3420. 10.1109/TNS.2011.2171363.

[ref33] Liu G.; Liu J.; Ye X.; Nie L.; Gu P.; Tao X.; Zhang Q. Self-Healing Behavior in a Thermo-Mechanically Responsive Cocrystal during a Reversible Phase Transition. Angew. Chem. Int. Ed. 2017, 56, 198–202. 10.1002/anie.201609667. 27930841

[ref34] Saha S.; Desiraju G. R. Using structural modularity in cocrystals to engineer properties: elasticity. Chem. Commun. 2016, 52, 7676–7679. 10.1039/C6CC03226E. 27228952

[ref35] Tothadi S.; Sanphui P.; Desiraju G. R. Obtaining Synthon Modularity in Ternary Cocrystals with Hydrogen Bonds and Halogen Bonds. Cryst. Growth Des. 2014, 14, 5293–5302. 10.1021/cg501115k.

[ref36] Mir N. A.; Dubey R.; Desiraju G. R. Strategy and Methodology in the Synthesis of Multicomponent Molecular Solids: The Quest for Higher Cocrystals. Acc. Chem. Res. 2019, 52, 2210–2220. 10.1021/acs.accounts.9b00211. 31318527

[ref37] Choppin G.; Liljenzin J.; Rydberg J.; Ekberg C. Radiochemistry and Nuclear Chemistry, 4th ed.; Elsevier, 2013; pp. 225–228.

[ref38] De Santis A.; Forni A.; Liantonio R.; Metrangolo P.; Pilati T.; Resnati G. N···Br Halogen Bonding: One-Dimensional Infinite Chans through the Self-Assembly of Dibromotetrafluorobenzenes with Dipyridyl Derivatives. Chemistry 2003, 9, 3974–3983. 10.1002/chem.200204655. 12916124

[ref39] Darwin C. G. XCII.The reflexion of X-rays from imperfect crystals. Philos. Mag. J. Sci. 1922, 43, 800–829. 10.1080/14786442208633940.

[ref40] Snyder R. L. The Use of Reference Intensity Ratios in X-ray Quantitative Analysis. Powder Diffr. 1992, 7, 186–193. 10.1017/S0885715600018686.

[ref41] Fairley M.; Myers N. M.; Szymanowski J. E. S.; Sigmon G. E.; Burns P. C.; LaVerne J. Z. Stability of Solid Uranyl Peroxides under Irradiation. Inorg. Chem. 2019, 58, 14112–14119. 10.1021/acs.inorgchem.9b02132. 31556996

[ref42] Busfield W. K.; O’Donnell J. H. Effects of Gamma Radiation on the Mechanical Properties and Crytallinity of Polypropylene Film. Eur. Polym. J. 1979, 15, 379–387. 10.1016/0014-3057(79)90157-5.

[ref43] Stojanovic Z.; Kavarevia-Popovic Z.; Galovic S.; Milicevic D.; Suljovrujic E. Crystallinity changes and melting behavior of the uniaxially oriented iPP exposed to high doses of gamma radiation. Polym. Degrad. Stab. 2005, 87, 279–286. 10.1016/j.polymdegradstab.2004.07.021.

[ref44] Tilak S.; Suresh Kumar H. M. Influence of gamma radiation on the properties of L-ascorbic acid crystal for photonic applications. J. Mater. Sci.: Mater. Electron. 2021, 32, 8174–8182. 10.1007/s10854-021-05543-z.

[ref45] Hossain M. S.; Shaikh M. A. A.; Rahaman M. S.; Ahmed S. Modification of the crystallographic parameters in a biomaterial employing a series of gamma radiation doses. Mol. Syst. Des. Eng. 2022, 7, 1239–1248. 10.1039/D2ME00061J.

[ref46] Yadav R. A.; Singh I. S. The Raman and Infrared Spectra and Normal Coordinate Analysis for 1,2-Diiodotetrafluorobenzene. J. Raman Spectrosc. 2005, 14, 353–357. 10.1002/jrs.1250140512.

[ref47] Hanson G. R.; Jensen P.; McMurtrie J.; Rintoul L.; Micallef A. S. Halogen Bonding between an Isoindoline Nitroxide and 1,4-Diiodotetrafluorobenzene: New Tools and Tecton for Self-Assembling Organic Spin Systems. Chem. A Eur. J.l 2009, 15, 4156–4164. 10.1002/chem.200801920. 19283822

[ref48] Green J. H. S.; Harrison D. J. Vibrational spectra of benzene derivatives—XVIII Dihalogenotetrafluorobenzenes. Spectrochim. Acta, Part A 1977, 33, 193–197. 10.1016/0584-8539(77)80014-7.

[ref49] Greaves S. J.; Griffith W. P. Vibrational spectra of catechol, catechol-*d_2_*, and -*d_6_* and the catecholate monoanion. Spectrochim. Acta, Part A 1991, 47, 133–140. 10.1016/0584-8539(91)80185-L.

[ref50] Onawole A. T.; Halim M. A.; Ullah N.; Al-Saadi A. A. Structural, spectroscopic and docking properties of resorcinol, its -OD isotopomer and dianion derivative: a comparative study. Struct. Chem. 2018, 29, 403–414. 10.1007/s11224-017-1037-5.

[ref51] Kubinyi M.; Billes F.; Grofcsik A.; Keresztury G. Vibrational spectra and normal coordinate analysis of phenol and hydroquinone. J. Mol. Struct. 1992, 266, 339–344. 10.1016/0022-2860(92)80089-Z.

[ref52] Meić Z.; Güsten H. Vibrational studies of *trans*-stilbenes—I. Infrared and Raman spectra of *trans*-stilbene and deuterated *trans*-stilbenes. Spectrochim. Acta, Part A 1978, 34, 101–111. 10.1016/0584-8539(78)80193-7.

[ref53] Yang W.; Hulteen J.; Schatz G. C.; Van Duyne R. P. A surface-enhanced hyper-Raman and surface-enhanced Raman scattering study of *trans*-1,2-bis(4-pyridyl)ethylene adsorbed onto silver film over nanosphere electrodes. Vibrational assignments: Experiment and theory. J. Chem. Phys. 1996, 104, 1413–4323. 10.1063/1.471241.

[ref54] Nikul P. V.; Maksimov A. M.; Platonov V. E.; Lotkov A. I.; Meisner L. L. Preparation of 1,4-Dibromotetrfluorobenzene from 4-Bromoetetrafluorobenzenethiol and Bromine. Reactions of Dibromotetrafluorobenzene with KSH. J. Fluorine Notes 2011, 2, 5–6.

[ref55] Biswas S.; Deshpande S. V.; Dunn D. N.; Narasimhan S. Tuning patterning conditions by co-adsorption of gases: Br_2_ and H_2_ of Si(001). J. Chem. Phys. 2013, 139, 184713 10.1063/1.4828702. 24320297

[ref56] Yu F.; Zhang X.; Zhao H.; Jiang Z.; Wang T.; Wang N.; Huang X.; Zhou L.; Hao H. Enhanced luminescence of single-benzene fluorescent molecules through halogen bond cocrystals. CrystEngComm 2022, 24, 3537–3545. 10.1039/D2CE00229A.

[ref57] Tamuly C.; Barooah N.; Laskar M.; Sarma R. J.; Baruah J. B. Fluorescence Quenching and Enhancement by H-bonding Interactions in Some Nitrogen Containing Fluorophores. Supramol. Chem. 2006, 18, 605–613. 10.1080/10610270601045537.

[ref58] Brahma R.; Pal Singh M.; Baruah J. B. Stacking among the clips of the poly-aromatic rings of phenazine with hydroxy-aromatics and photophysical properties. RSC Adv. 2019, 9, 33403–33412. 10.1039/C9RA07602F. 35529104 PMC9073320

[ref59] Gao H. Y.; Shen Q. J.; Zhao X. R.; Yan X. Q.; Pang X.; Jin W. J. Phosphorescent co-crystal assembled by 1,4-diiodotetrafluorobenzene with carbazole, based on C-I···π halogen bonding. J. Mater. Chem. 2012, 22, 5336–5343. 10.1039/c2jm16257a.

[ref60] Li L.; Liu Z. F.; Wu W. X.; Jin W. J. Cocrystals with tunable luminescence colour self-assembled by a predictable method. Acta Crystallogr. B 2018, 74, 610–617. 10.1107/S2052520618013483.

[ref61] Hirayama F.; Lipsky S. The effect of crystalline phase on the fluorescence characteristics of solid cyclohexane and bicyclohexyl. Chem. Phys. Lett. 1973, 22, 172–176. 10.1016/0009-2614(73)80563-9.

[ref62] Zhu H.; Huang J.; Kong L.; Tian Y.; Yang J. Branched triphenylamine luminophores: Aggregation-induced fluorescence emission, and tunable near-infrared solid-state fluorescence characteristics *via* external mechanical stimuli. Dye Pigments 2018, 151, 140–148. 10.1016/j.dyepig.2017.12.053.

[ref63] Agullo-Lopez F. Dependence of the room temperature F-centre coloration of the irradiation dose sequence. Solid State Commun. 1966, 4, 275–277. 10.1016/0038-1098(66)90451-0.

