# Supplementary material for: Effects of Gamma Radiation on Single- and Multicomponent Organic Crystalline Materials
Source: Cryst Growth Des. 2023 Mar 2;23(5):3357–66. doi: 10.1021/acs.cgd.2c01504 (PMC10162448; doi:10.1021/acs.cgd.2c01504)
Supplement: Supplementary file 1 — cg2c01504_si_001.pdf [file cg2c01504_si_001.pdf]

Supporting Information Section

Effects of Gamma Radiation on Single- and Multi-Component Organic Crystalline Materials

*Samantha J. Kruse, Len R. MacGillivray, and Tori Z. Forbes**

Department of Chemistry, University of Iowa Chemistry Building, Iowa City, IA, 52242 (USA)

Supporting Information

Table of Contents

Crystallographic Information

Crystallographic information and unit cell percent changes for pre- and post-irradiated forms of:

Table S1: 1,2-$C_6H_6O_2$……7

Table S2: 1,3-$C_6H_6O_2$……7

Table S3: 1,4-$C_6H_6O_2$……7

Table S4: *trans*-stilbene……8

Table S5: 4,4'-bpe……8

Table S6: (4,4'-bpe)(1,2-$C_6I_2F_4$)……8

Table S7: (4,4'-bpe)(1,3-$C_6I_2F_4$)……9

Table S8: (4,4'-bpe)(1,4-$C_6I_2F_4$)……9

Table S9: (4,4'-bpe)(1,2-$C_6Br_2F_4$)……9

Table S10: (4,4'-bpe)(1,3-$C_6Br_2F_4$)……10

Table S11: (4,4'-bpe)(1,4-$C_6Br_2F_4$)……10

Table S12: (4,4'-bpe)(1,2-$C_6H_6O_2$)……10

Table S13: (4,4'-bpe)(1,3-$C_6H_6O_2$)……,,,……11

Table S14: (4,4'-bpe)(1,4-$C_6H_6O_2$)……11

Powder X-ray Diffraction

Background subtracted powder patterns for:

Figure S1: 1,*n*-$C_6I_2F_4$ (where n = 2 or 4)……12

Figure S2: 1,*n*-$C_6H_6O_2$ (where n = 2, 3 or 4)……13

Figure S3: ***trans*-stilbene** and **4,4'-bpe**..........**14**

Figure S4: (**4,4'-bpe**)·(**1,*n*-$C_6I_2F_4$**) (where n = 2, 3 or 4)..........**15**

Figure S5: (**4,4'-bpe**)·(**1,*n*-$C_6Br_2F_4$**) (where n = 2, 3 or 4)..........**16**

Figure S6: (**4,4'-bpe**)·(**1,*n*-$C_6H_6O_2$**) (where n = 2, 3 or 4)..........**17**

Powder patterns pre- and post-irradiation without background subtraction for:

Figure S7: **1,2-$C_6I_2F_4$**..........**18**

Figure S8: **1,4-$C_6I_2F_4$**..........**18**

Figure S9: **1,4-$C_6Br_2F_4$**..........**19**

Figure S10: **1,2--$C_6H_6O_2$**..........**19**

Figure S11: **1,3-$C_6H_6O_2$**..........**20**

Figure S12: **1,4-$C_6H_6O_2$**..........**20**

Figure S13: ***trans*-stilbene**..........**21**

Figure S14: **4,4'-bpe**..........**21**

Figure S15: **(4,4'-bpe)□(1,2-$C_6I_2F_4$)**..........**22**

Figure S16: **(4,4'-bpe)□(1,3-$C_6I_2F_4$)**..........**22**

Figure S17: **(4,4'-bpe)□(1,4-$C_6I_2F_4$)**..........**23**

Figure S18: **(4,4'-bpe)□(1,2-$C_6Br_2F_4$)**..........**23**

Figure S19: **(4,4'-bpe)□(1,3-$C_6Br_2F_4$)**..........**24**

Figure S20: **(4,4'-bpe)□(1,4-$C_6Br_2F_4$)**..........**24**

Figure S21: **(4,4'-bpe)□(1,2-$C_6H_6O_2$)**..........**25**

Figure S22: **(4,4'-bpe)□(1,3-$C_6H_6O_2$)**..........**25**

Figure S23: **(4,4'-bpe)□(1,4-$C_6H_6O_2$)**..........**26**

Powder pattern intensities and percent changes for:

Table S15: 1,2-$C_6I_2F_4$……27

Table S16: 1,4-$C_6I_2F_4$……27

Table S17: 1,4-$C_6Br_2F_4$……27

Table S18: 1,2-$C_6H_6O_2$……28

Table S19: 1,3-$C_6H_6O_2$……28

Table S20: 1,4-$C_6H_6O_2$……28

Table S21: *trans*-stilbene……28

Table S22: 4,4'-bpe……29

Table S23: (4,4'-bpe)(1,2-$C_6I_2F_4$)……29

Table S24: (4,4'-bpe)(1,3-$C_6I_2F_4$)……29

Table S25: (4,4'-bpe)(1,4-$C_6I_2F_4$)……29

Table S26: (4,4'-bpe)(1,2-$C_6Br_2F_4$)……30

Table S27: (4,4'-bpe)(1,3-$C_6Br_2F_4$)……30

Table S28: (4,4'-bpe)(1,4-$C_6Br_2F_4$)……30

Table S29: (4,4'-bpe)(1,2-$C_6H_6O_2$)……30

Table S30: (4,4'-bpe)(1,3-$C_6H_6O_2$)……31

Table S31: (4,4'-bpe)(1,4-$C_6H_6O_2$)……31

Raman Spectroscopy

1,2-$C_6I_2F_4$:

Figure S24: Fitted Raman spectrum and band assignments pre-irradiation……32

Figure S25: Fitted Raman spectrum and band assignments for post-irradiation……33

1,4-$C_6I_2F_4$:

Figure S26: Fitted Raman spectrum and band assignments for pre-irradiation……34

Figure S27: Fitted Raman spectrum and band assignments for post-irradiation………..…...…..**34**

1,4-$C_6Br_2F_4$:

Figure S28: Fitted Raman spectrum and band assignments for pre-irradiation…..….......……...**35**

Figure S29: Fitted Raman spectrum and band assignments for post-irradiation……..…...….....**35**

1,2-$C_6H_6O_2$:

Figure S30: Fitted Raman spectrum and band assignments for pre-irradiation…..……..............**36**

Figure S31: Fitted Raman spectrum and band assignments for post-irradiation………..…...…..**37**

1,3-$C_6H_6O_2$:

Figure S32: Fitted Raman spectrum and band assignments for pre-irradiation…..…..….....…...**38**

Figure S33: Fitted Raman spectrum and band assignments for post-irradiation…..…………...…..**39**

1,4-$C_6H_6O_2$:

Figure S34: Fitted Raman spectrum and band assignments for pre-irradiation…..……......…...**40**

Figure S35: Fitted Raman spectrum and band assignments for post-irradiation………..…...…..**41**

***trans*-stilbene :**

Figure S36: Fitted Raman spectrum and band assignments for pre-irradiation…..…...……..…...**42**

Figure S37: Fitted Raman spectrum and band assignments for post-irradiation………..…..…....**43**

4,4’-bpe :

Figure S38: Fitted Raman spectrum and band assignments for pre-irradiation…..…...…..……...**43**

Figure S39: Fitted Raman spectrum and band assignments for post-irradiation……...……..…..**44**

(4,4’-bpe)□(1,2-$C_6I_2F_4$) :

Figure S40: Fitted Raman spectrum and band assignments for pre-irradiation………..….……..**44**

Figure S41: Fitted Raman spectrum and band assignments for post-irradiation.…..…...……....**45**

(4,4’-bpe)□(1,3-$C_6I_2F_4$) :

Figure S42: Fitted Raman spectrum and band assignments for pre-irradiation…........................**45**

Figure S43: Fitted Raman spectrum and band assignments for post-irradiation.…..……..….....**46**

(4,4’-bpe)□(1,4-$C_6I_2F_4$) :

Figure S44: Fitted Raman spectrum and band assignments for pre-irradiation…………...……..**46**

Figure S45: Fitted Raman spectrum and band assignments for post-irradiation…………..…….**47**

(4,4’-bpe)□(1,2-$C_6Br_2F_4$) :

<u>Figure S46:</u> Fitted Raman spectrum and band assignments for pre-irradiation……….**47**

<u>Figure S47:</u> Fitted Raman spectrum and band assignments for post-irradiation……….**48**

(4,4’-bpe)□(1,3-$C_6Br_2F_4$) :

<u>Figure S48:</u> Fitted Raman spectrum and band assignments for pre-irradiation……….**48**

<u>Figure S49:</u> Fitted Raman spectrum and band assignments for post-irradiation……….**49**

(4,4’-bpe)□(1,4-$C_6Br_2F_4$) :

<u>Figure S50:</u> Fitted Raman spectrum and band assignments for pre-irradiation……….**49**

<u>Figure S51:</u> Fitted Raman spectrum and band assignments for post-irradiation……….**50**

Solid-State Fluorimetry

Solid-state fluorescence spectra overlay of pre- and post-radiation for:

<u>Figure S52:</u> 1,2-$C_6I_2F_4$……….51

<u>Figure S53:</u> 1,4-$C_6I_2F_4$……….52

<u>Figure S54:</u> 1,4-$C_6Br_2F_4$……….53

<u>Figure S55:</u> 1,2-$C_6H_6O_2$……….54

<u>Figure S56:</u> 1,3-$C_6H_6O_2$……….55

<u>Figure S57:</u> 1,4-$C_6H_6O_2$……….56

<u>Figure S58:</u> (4,4’-bpe)□(1,2-$C_6I_2F_4$)……….57

<u>Figure S59:</u> (4,4’-bpe)□(1,3-$C_6I_2F_4$)……….58

<u>Figure S60:</u> (4,4’-bpe)□(1,4-$C_6I_2F_4$)……….59

<u>Figure S61:</u> (4,4’-bpe)□(1,2-$C_6Br_2F_4$)……….60

<u>Figure S62:</u> (4,4’-bpe)□(1,3-$C_6Br_2F_4$)……….61

<u>Figure S63:</u> (4,4’-bpe)□(1,2-$C_6H_6O_2$)……….62

Figure S64: (4,4’-bpe)□(1,3-$C_6H_6O_2$)……63

Figure S65: (4,4’-bpe)□(1,4-$C_6H_6O_2$)……64

Differential Scanning Calorimetry

Integrated differential thermogram of:

Figure S66: 1,2-$C_6I_2F_4$ pre-radiation……65

Figure S67: 1,2-$C_6I_2F_4$ post-radiation……66

Figure S68: 1,4-$C_6I_2F_4$ pre-radiation……67

Figure S69: 1,4-$C_6I_2F_4$ post-radiation……68

Figure S70: 1,4-$C_6Br_2F_4$ pre-radiation……69

Figure S71: 1,4-$C_6Br_2F_4$ post-radiation……70

Post-Radiation Sublimation

Figure S72: 1,4-$C_6Br_2F_4$ powder post-irradiation undergoing sublimation……70

References……71

Crystallographic Information

Crystallographic information and unit cell percent changes

Table S1. Crystal unit cell parameters for **1,2-$C_6H_6O_2$** pre- (left) and post-radiation (middle) along with unit cell parameter percent changes (right).

Pre-Irradiation				Post-Irradiation				Unit Cell Percent Changes			
Monoclinic P2_1*/n*				*Monoclinic P*2_1*/n*				Same Space Group			
a/Å	9.779(8)	α/°	90	a/Å	9.781(7)	α/°	90	a	0.020%	α	-
b/Å	5.650(4)	β/°	112.14(2)	b/Å	5.641(4)	β/°	114.18(4)	b	0.159%	β	0.450%
c/Å	10.387(5)	γ/°	90	c/Å	10.388(6)	γ/°	90	c	0.010%	γ	-
V/Å^3	523.1(2)	Z	4	V/Å^3	522.9(6)	Z	4	V	0.010%	Z	-
R_{int}	3.15%	R_1	1.89%	R_{int}	3.21%	R_1	1.72%				
GooF = 1.112				GooF = 1.279							

Table S2. Crystal unit cell parameters for **1,3-$C_6H_6O_2$** pre- (left) and post-radiation (middle) along with unit cell parameter percent changes (right).

Pre-Irradiation				Post-Irradiation				Unit Cell Percent Changes			
Orthorhombic Pna2_1				*Orthorhombic Pna*2_1				Same Space Group			
a/Å	10.4604(5)	α/°	90	a/Å	10.4544(10)	α/°	90	a	0.014%	α	-
b/Å	9.4054(5)	β/°	90	b/Å	9.3824(8)	β/°	90	b	0.061%	β	-
c/Å	5.6595(3)	γ/°	90	c/Å	5.6588(5)	γ/°	90	c	0.003%	γ	-
V/Å^3	556.81(5)	Z	16	V/Å^3	555.06(9)	Z	16	V	0.079%	Z	-
R_{int}	3.47%	R_1	2.59%	R_{int}	4.63	R_1	3.23%				
GooF = 1.129				GooF = 1.018							

Table S3. Crystal unit cell parameters for **1,4-$C_6H_6O_2$** pre- (left) and post-radiation (middle) along with unit cell parameter percent changes (right).

Pre-Irradiation				Post-Irradiation				Unit Cell Percent Changes			
Rhombahedral R-3				*Rhombahedral R*-3				Same Space Group			
a/Å	38.256(8)	α/°	90.00(3)	a/Å	38.215(8)	α/°	90.00(3)	a	0.027%	α	-
b/Å	38.256(8)	β/°	90.00(3)	b/Å	38.215(8)	β/°	90.00(3)	b	0.027%	β	-
c/Å	5.593(2)	γ/°	120.00(3)	c/Å	5.580(2)	γ/°	120.00(3)	c	0.058%	γ	-
V/Å^3	7089.(4)	Z	6	V/Å^3	7057.(4)	Z	6	V	0.113%	Z	-
R_{int}	5.24%	R_1	3.89%	R_{int}	5.94%	R_1	4.14%				
GooF = 1.194				GooF = 1.154							

Table S4. Crystal unit cell parameters for ***trans*-stilbene** pre- (left) and post-radiation (middle) along with unit cell parameter percent changes (right).

Pre-Irradiation				Post-Irradiation				Unit Cell Percent Changes			
Monoclinic $P2_1/c$				*Monoclinic $P2_1/c$*				Same Space Group			
a/Å	15.4733(12)	α/°	90	a/Å	15.4616(8)	α/°	90	a	0.019%	α	-
b/Å	5.6724(5)	β/°	112.090(3)	b/Å	5.6589(3)	β/°	112.175(2)	b	0.060%	β	0.02%
c/Å	12.2798(9)	γ/°	90	c/Å	12.2921(6)	γ/°	90	c	0.025%	γ	-
V/Å^3	998.7(1)	Z	4	V/Å^3	995.9(1)	Z	4	V	0.070%	Z	-
R_{int}	5.53%	R_1	5.39%	R_{int}	3.04%	R_1	5.18%				
GooF = 1.120				GooF = 1.142							

Table S5. Crystal unit cell parameters for **4,4'-bpe** pre- (left) and post-radiation (middle) along with unit cell parameter percent changes (right).

Pre-Irradiation				Post-Irradiation				Unit Cell Percent Changes			
Monoclinic *$P2_1/c$*				Monoclinic *$P2_1/c$*				Same Space Group			
a/Å	5.7301(1)	α/°	90	a/Å	5.8263(4)	α/°	90	a	0.416%	α	-
b/Å	10.5202(2)	β/°	91.920(6)	b/Å	10.5360(6)	β/°	91.754(3)	b	0.393%	β	0.00%
c/Å	7.6131(1)	γ/°	90	c/Å	7.5606(5)	γ/°	90	c	0.173%	γ	-
V/Å^3	458.6(1)	Z	4	V/Å^3	455.93(5)	Z	4	V	0.146%	Z	-
R_{int}	4.08%	R_1	3.75%	R_{int}	4.15%	R_1	3.82%				
GooF = 1.136				GooF =							

Table S6. Crystal unit cell parameters for (**4,4'-bpe**)·(**1,2-$C_6I_2F_4$**) pre- (left) and post-radiation (middle) along with unit cell parameter percent changes (right).

Pre-Irradiation				Post-Irradiation				Unit Cell Percent Changes			
Monoclinic $P2_1/c$				*Monoclinic $P2_1/c$*				Same Space Group			
a/Å	8.0222(9)	α/°	90	a/Å	8.0224(2)	α/°	90	a	0.001%	α	-
b/Å	11.9061(6)	β/°	96.924(3)	b/Å	11.9052(8)	β/°	96.942(3)	b	0.002%	β	0.005%
c/Å	19.2015(4)	γ/°	90	c/Å	19.2010(14)	γ/°	90	c	0.001%	γ	-
V/Å^3	1822.8(4)	Z	4	V/Å^3	1820.4(2)	Z	4	V	0.033%	Z	-
R_{int}	3.95%	R_1	1.90%	R_{int}	2.93%	R_1	1.85%				
GooF = 1.102				GooF = 1.052							

Table S7. Crystal unit cell parameters for (**4,4'-bpe**)·(**1,3-$C_6I_2F_4$**) pre- (left) and post-radiation (middle) along with unit cell parameter percent changes (right).

Pre-Irradiation				Post-Irradiation				Unit Cell Percent Changes			
Monoclinic *P*2$_1$/*n*				Monoclinic *P*2$_1$/*n*				Same Space Group			
a/Å	14.7545(1)	α/°	90	a/Å	14.7541(6)	α/°	90	a	0.001%	α	-
b/Å	6.1306(5)	β/°	101.545(2)	b/Å	6.1315(8)	β/°	101.551(3)	b	0.004%	β	0.00%
c/Å	20.378(2)	γ/°	90	c/Å	20.374(2)	γ/°	90	c	0.005%	γ	-
V/Å^3	1806.0(2)	Z	4	V/Å^3	1802.1(3)	Z	4	V	0.005%	Z	-
R_{int}	4.79%	R_1	3.36%	R_{int}	4.92%	R_1	4.02%				
GooF = 1.072				GooF = 1.122							

Table S8. Crystal unit cell parameters for (**4,4'-bpe**)·(**1,4-$C_6I_2F_4$**) pre- (left) and post-radiation (middle) along with unit cell parameter percent changes (right).

Pre-Irradiation				Post-Irradiation				Unit Cell Percent Changes			
Triclinic P-1				*Triclinic P*-1				Same Space Group			
a/Å	6.2392(3)	α/°	84.929(2)	a/Å	6.2418(4)	α/°	84.891(2)	a	0.010%	α	0.011%
b/Å	8.3137(4)	β/°	71.125(2)	b/Å	8.3187(5)	β/°	71.092(2)	b	0.000%	β	0.012%
c/Å	9.1093(5)	γ/°	79.199(2)	c/Å	9.1044(5)	γ/°	79.157(2)	c	0.015%	γ	0.013%
V/Å^3	438.98(4)	Z	2	V/Å^3	439.04(5)	Z	2	V	0.003%	Z	-
R_{int}	0.0464	R_1	0.0391	R_{int}	3.02%	R_1	2.01%				
GooF = 1.084				GooF = 1.142							

Table S9. Crystal unit cell parameters for (**4,4'-bpe**)·(**1,2-$C_6Br_2F_4$**) pre- (left) and post-radiation (middle) along with unit cell parameter percent changes (right).

Pre-Irradiation				Post-Irradiation				Unit Cell Percent Changes			
*Monoclinic P*2$_1$/*c*				*Monoclinic P*2$_1$/*c*				Same Space Group			
a/Å	9.8592(8)	α/°	90	a/Å	9.8617(3)	α/°	90	a	0.006%	α	-
b/Å	7.9699(7)	β/°	98.417(3)	b/Å	7.9942(3)	β/°	98.388(2)	b	0.076%	β	0.020%
c/Å	16.076(1)	γ/°	90	c/Å	16.0703(6)	γ/°	90	c	0.009%	γ	-
V/Å^3	1249.6(3)	Z	4	V/Å^3	1253.37(8)	Z	4	V	0.075%	Z	-
R_{int}	3.93%	R_1	3.17%	R_{int}	4.72%	R_1	2.09%				
GooF = 1.096				GooF = 1.228							

Table S10. Crystal unit cell parameters for (**4,4'-bpe**)·(**1,3-$C_6Br_2F_4$**) pre- (left) and post-radiation (middle) along with unit cell parameter percent changes (right).

Pre-Irradiation				Post-Irradiation				Unit Cell Percent Changes			
Monoclinic P2_1/*c*				*Monoclinic P*2_1/*c*				Same Space Group			
a/Å	7.8650(4)	α/°	90	a/Å	7.8779(4)	α/°	90	a	0.041%	α	-
b/Å	5.9641(5)	β/°	94.723(2)	b/Å	5.9636(3)	β/°	94.747(2)	b	0.002%	β	0.006%
c/Å	35.9540(1)	γ/°	90	c/Å	35.9535(19)	γ/°	90	c	0.0003%	γ	-
V/Å^3	1685.14(2)	Z	4	V/Å^3	1683.32(15)	Z	4	V	0.027%	Z	-
R_{int}	4.16%	R_1	4.51%	R_{int}	6.27%	R_1	5.32%				
GooF = 1.054				GooF = 1.291							

Table S11. Crystal unit cell parameters for (**4,4'-bpe**)·(**1,4-$C_6Br_2F_4$**) pre- (left) and post-radiation (middle) along with unit cell parameter percent changes (right).

Pre-Irradiation				Post-Irradiation				Unit Cell Percent Changes			
Triclinic P-1				*Triclinic P*-1				Same Space Group			
a/Å	6.041(3)	α/°	82.72(4)	a/Å	6.0461(6)	α/°	83.150(6)	a	0.021%	α	0.130%
b/Å	6.900(5)	β/°	86.54(2)	b/Å	6.8303(7)	β/°	86.549(6)	b	0.254%	β	0.003%
c/Å	11.069(6)	γ/°	68.31(3)	c/Å	11.069(1)	γ/°	68.533(5)	c	0.000%	γ	0.081%
V/Å^3	425.2(4)	Z	2	V/Å^3	422.30(8)	Z	2	V	0.171%	Z	-
R_{int}	3.65%	R_1	2.01%	R_{int}	4.38%	R_1	3.35%				
GooF = 1.095				GooF = 1.131							

Table S12. Crystal unit cell parameters for (**4,4'-bpe**)·(**1,2-$C_6H_6O_2$**) pre- (left) and post-radiation (middle) along with unit cell parameter percent changes (right).

Pre-Irradiation				Post-Irradiation				Unit Cell Percent Changes			
*Monoclinic P*2/*c*				*Monoclinic P*2/*c*				Same Space Group			
a/Å	23.880(1)	α/°	90	a/Å	23.8798(13)	α/°	90	a	0.002%	α	-
b/Å	7.3556(5)	β/°	108.923(2)	b/Å	7.3593(4)	β/°	108.933(2)	b	0.013%	β	0.002%
c/Å	17.513(1)	γ/°	90	c/Å	17.5380(10)	γ/°	90	c	0.036%	γ	-
V/Å^3	2909.9(3)	Z	2	V/Å^3	2915.4(3)	Z	4	V	0.047%	Z	-
R_{int}	7.10%	R_1	4.98%	R_{int}	5.59%	R_1	4.59%				
GooF = 1.021				GooF = 1.096							

Table S13. Crystal unit cell parameters for (**4,4'-bpe**)·(**1,3-$C_6H_6O_2$**) pre- (left) and post-radiation (middle) along with unit cell parameter percent changes (right).

Pre-Irradiation				Post-Irradiation				Unit Cell Percent Changes			
Triclinic P-1				*Triclinic P*-1				Same Space Group			
a/Å	8.0080(12)	α/°	73.123(4)	a/Å	8.0391(4)	α/°	73.139(2)	a	0.097%	α	0.005%
b/Å	9.8121(14)	β/°	72.594(5)	b/Å	9.8107(4)	β/°	72.449(2)	b	0.004%	β	0.050%
c/Å	10.8650(16)	γ/°	66.150(4)	c/Å	10.8613(5)	γ/°	66.135(2)	c	0.009%	γ	0.006%
V/Å^3	730.90(19)	Z	2	V/Å^3	732.91(6)	Z	2	V	0.069%	Z	-
R_{int}	7.44%	R_1	4.70%	R_{int}	6.57%	R_1	4.44%				
GooF = 1.114				GooF = 1.111							

Table S14. Crystal unit cell parameters for (**4,4'-bpe**)·(**1,4-$C_6H_6O_2$**) pre- (left) and post-radiation (middle) along with unit cell parameter percent changes (right).

Pre-Irradiation				Post-Irradiation				Unit Cell Percent Changes			
Monoclinic $P2_1/c$				*Monoclinic* $P2_1/c$				Same Space Group			
a/Å	5.9917(5)	α/°	90	a/Å	5.9953(2)	α/°	90	a	0.015%	α	-
b/Å	17.2490(13)	β/°	92.359(4)	b/Å	17.2604(6)	β/°	92.346(2)	b	0.017%	β	0.004%
c/Å	7.0790(5)	γ/°	90	c/Å	7.0968(2)	γ/°	90	c	0.063%	γ	-
V/Å^3	731.00(10)	Z	4	V/Å^3	733.77(4)	Z	4	V	0.095%	Z	-
R_{int}	4.97%	R_1	3.93%	R_{int}	3.57%	R_1	5.35%				
GooF = 1.171				GooF = 1.170							

Powder X-ray Diffraction

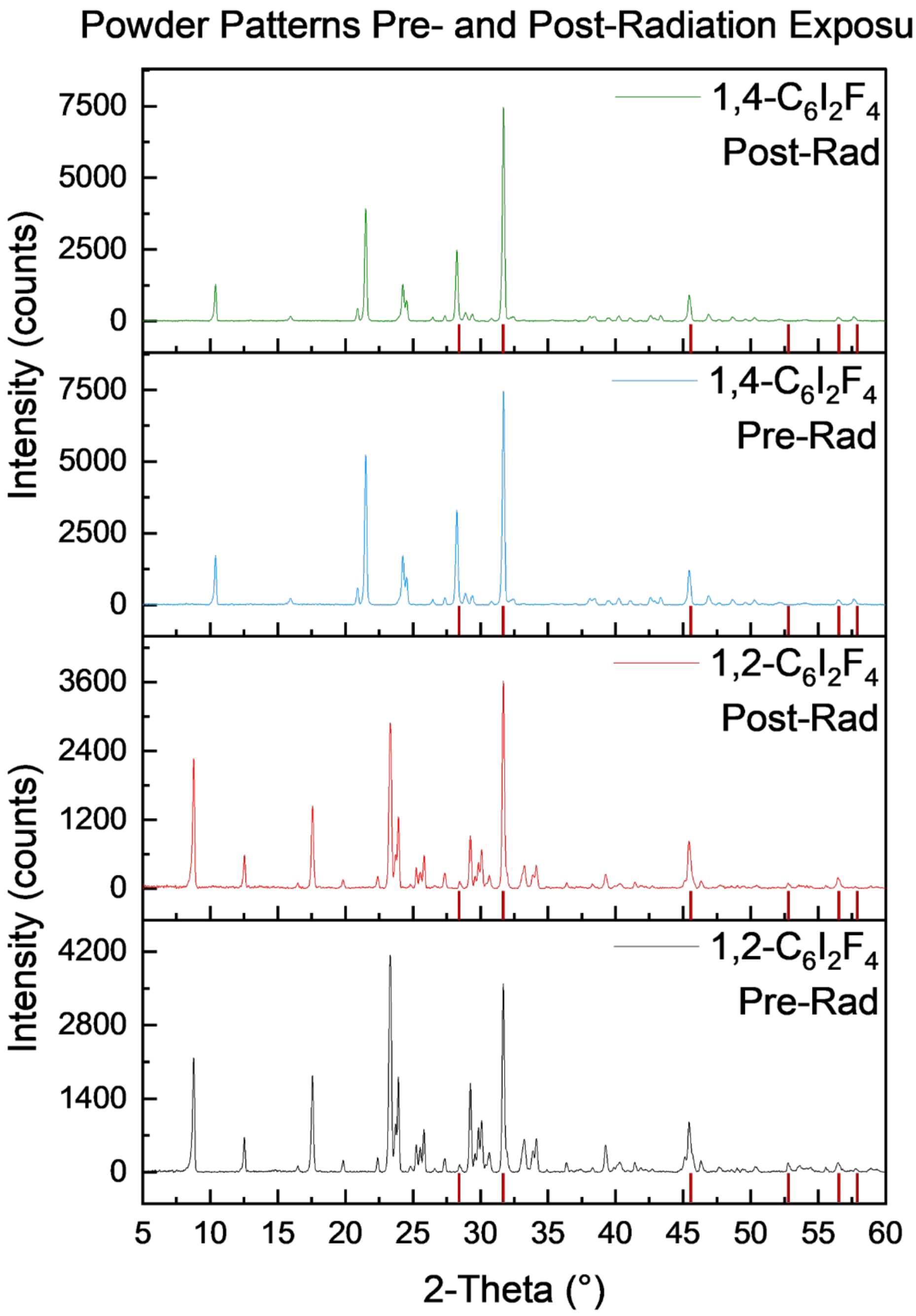

Figure S1. Normalized powder patterns pre- and post-irradiation for single component samples **1,*n*-$C_6I_2F_4$** (where $n = 2$ or 4). Red lines on x-axis represent NaCl peaks.

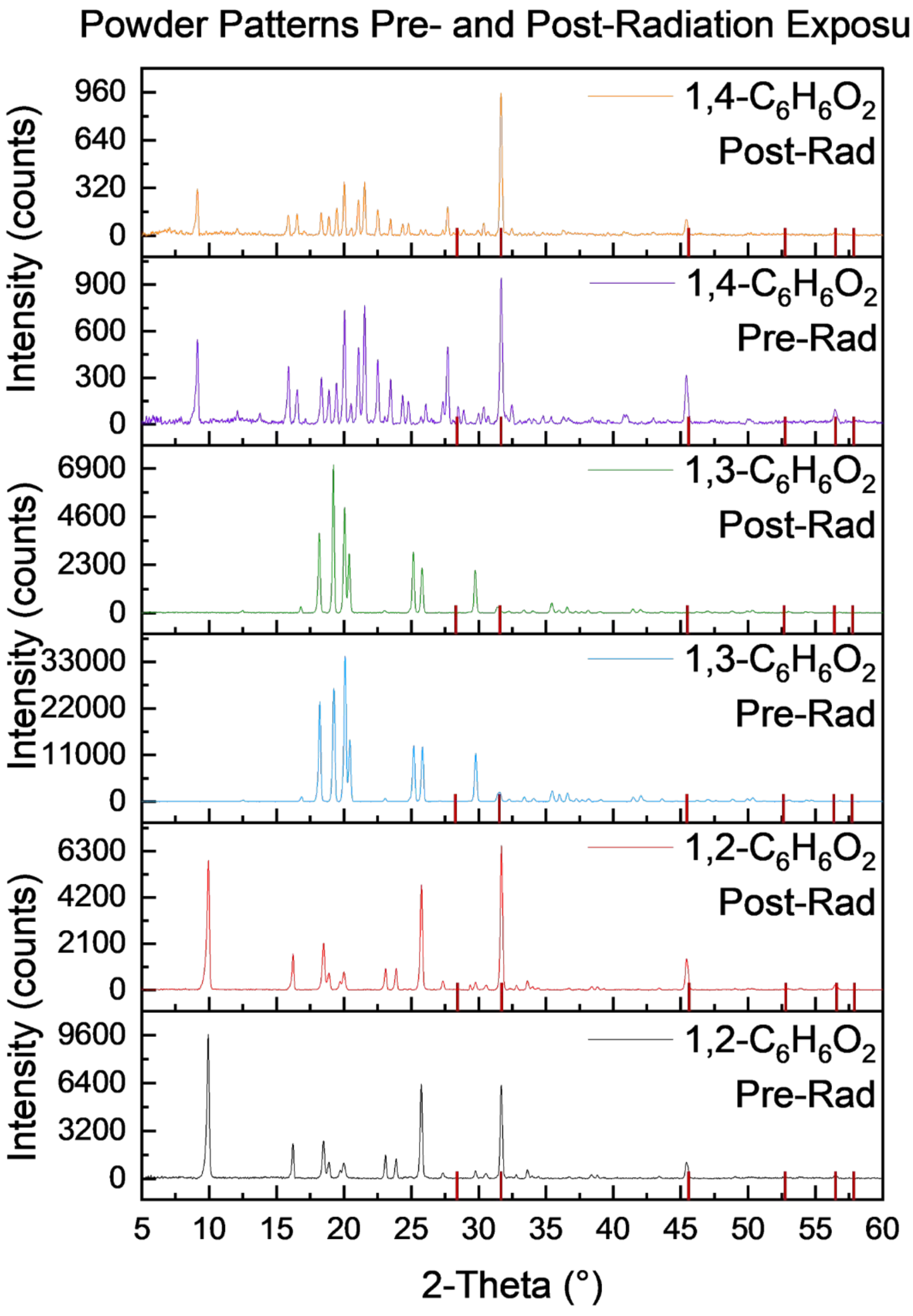

Figure S2. Normalized powder patterns pre- and post-irradiation for single component samples **1,*n*-$C_6H_6O_2$** (where n = 2, 3 or 4). Red lines on x-axis represent NaCl peaks.

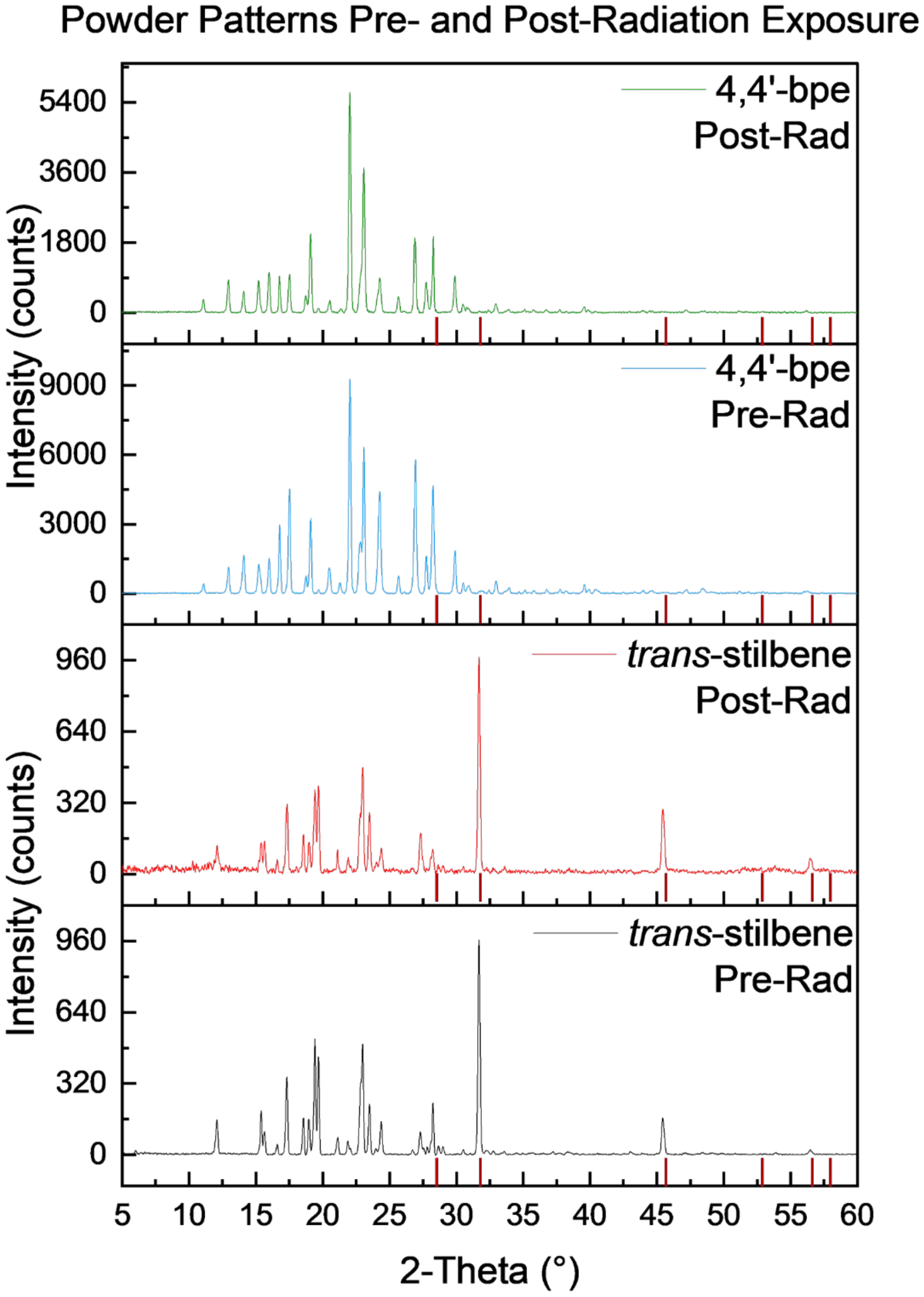

Figure S3. Normalized powder patterns pre- and post-irradiation for ***trans*-stilbene** and **4,4'-bpe**. Red lines on x-axis represent NaCl peaks.

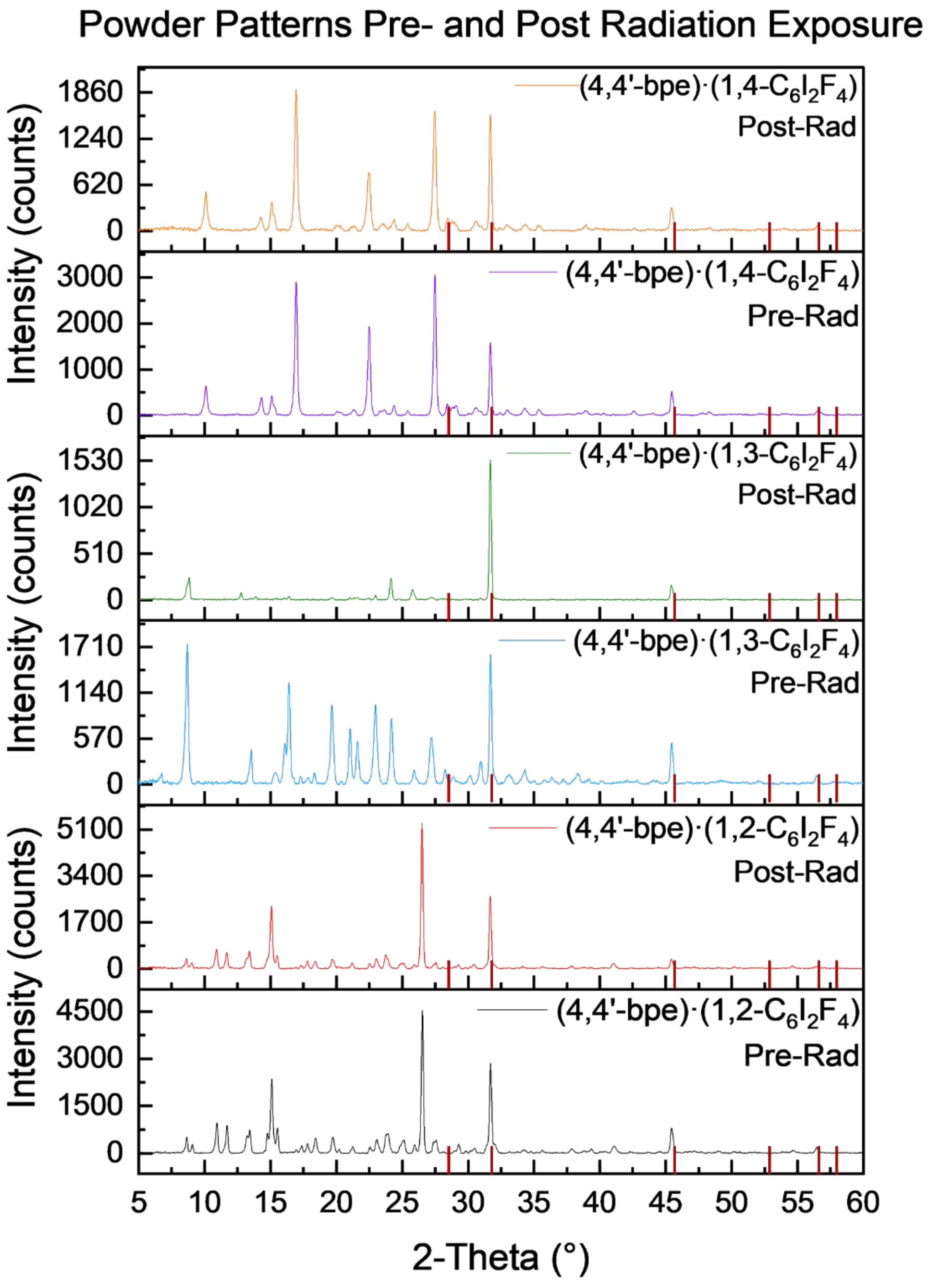

Figure S4. Normalized powder patterns pre- and post-irradiation for multicomponent samples (**4,4'-bpe**)·(**1,*n*-$C_6I_2F_4$**). (where n = 2, 3 or 4). Red lines on x-axis represent NaCl peaks.

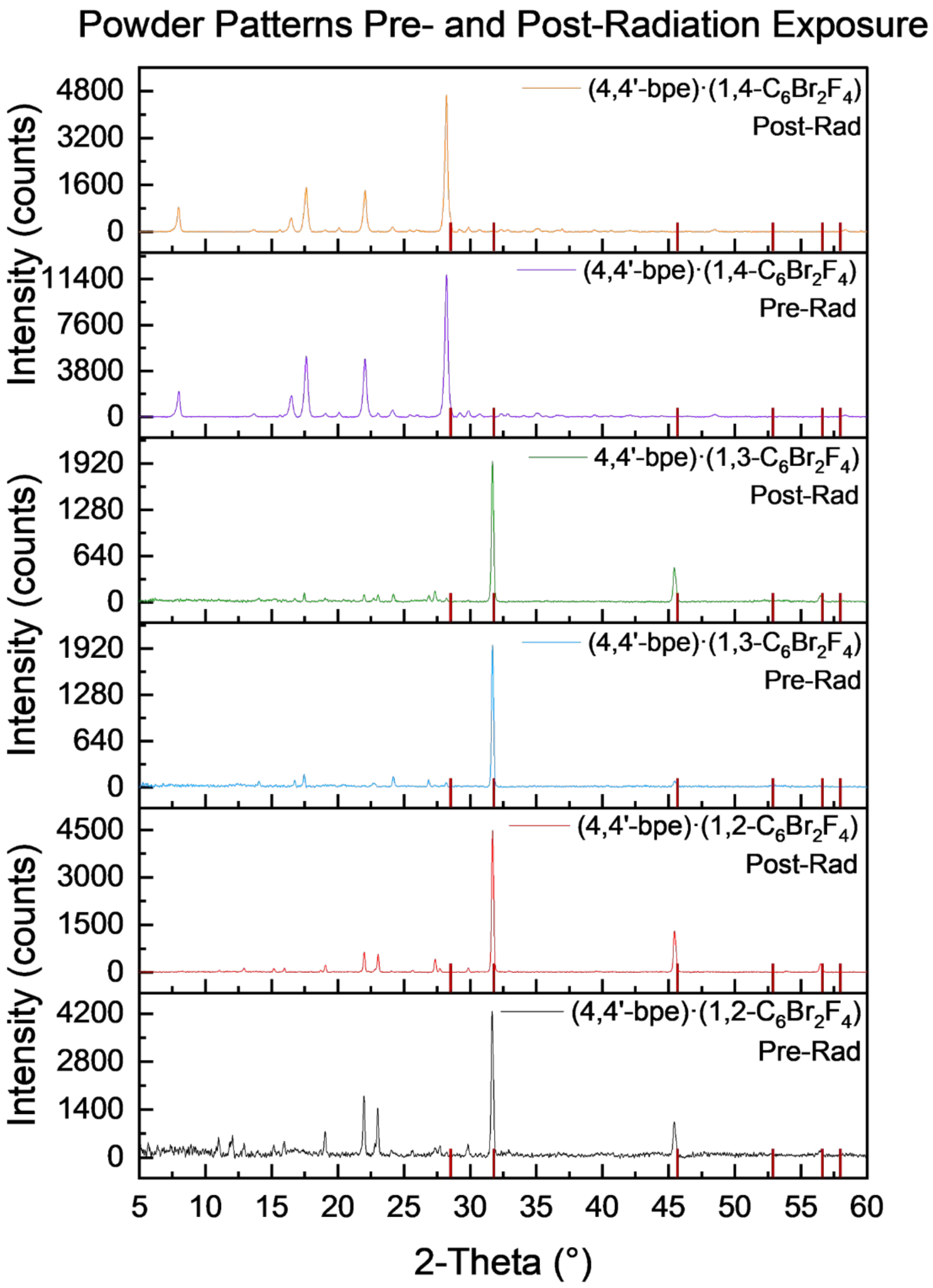

Figure S5. Normalized powder patterns pre- and post-irradiation for multicomponent samples (**4,4'-bpe**)·(**1,*n*-$C_6Br_2F_4$**). (where *n* = 2, 3 or 4). Red lines on x-axis represent NaCl peaks.

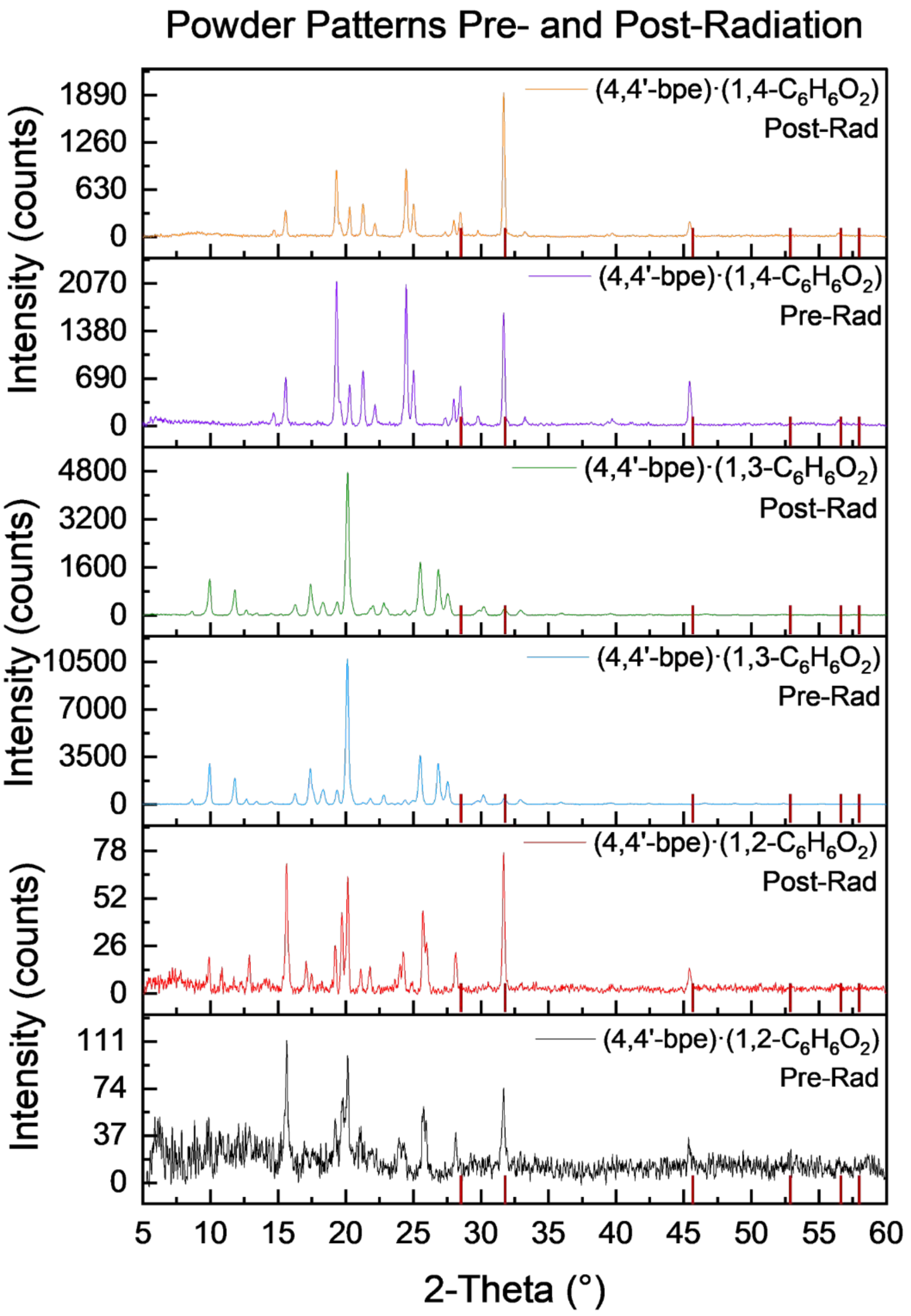

Figure S6. Normalized powder patterns pre- and post-irradiation for multicomponent samples (**4,4'-bpe**)·(**1,*n*-$C_6H_6O_2$**). (where n = 2, 3 or 4). Red lines on x-axis represent NaCl peaks.

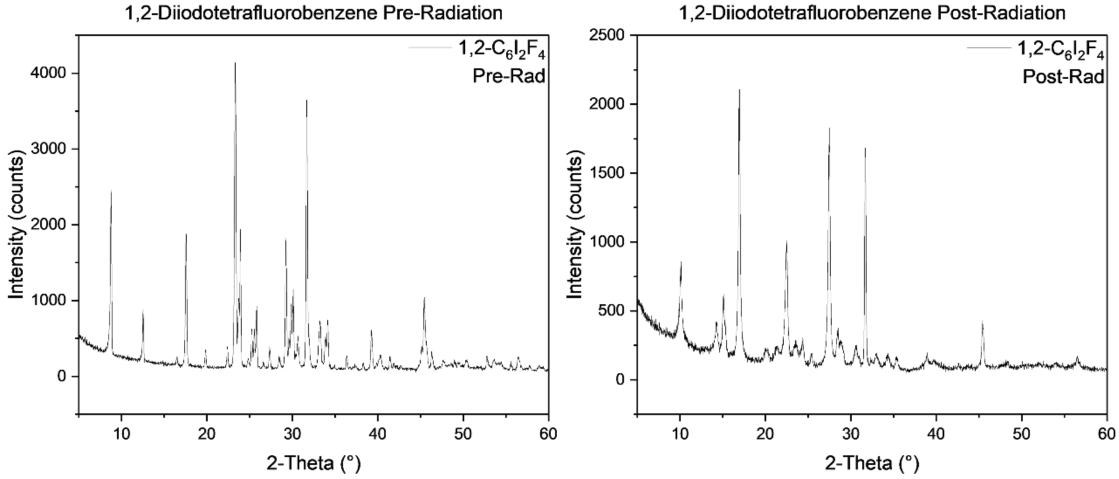

Figure S7. Powder pattern **1,2-$C_6I_2F_4$** pre- (left) and post-radiation (right) exposure without background subtraction.

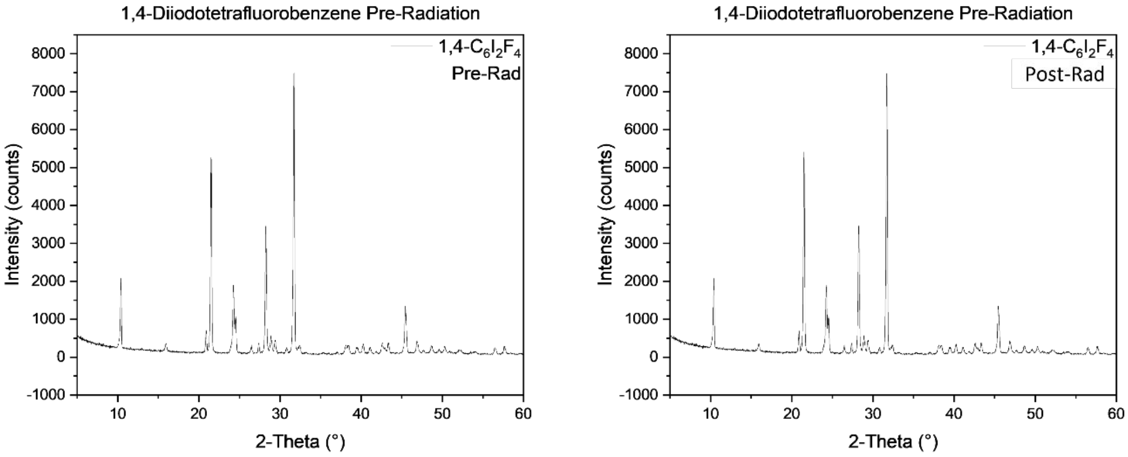

Figure S8. Powder pattern **1,4-$C_6I_2F_4$** pre- (left) and post-radiation (right) exposure without background subtraction.

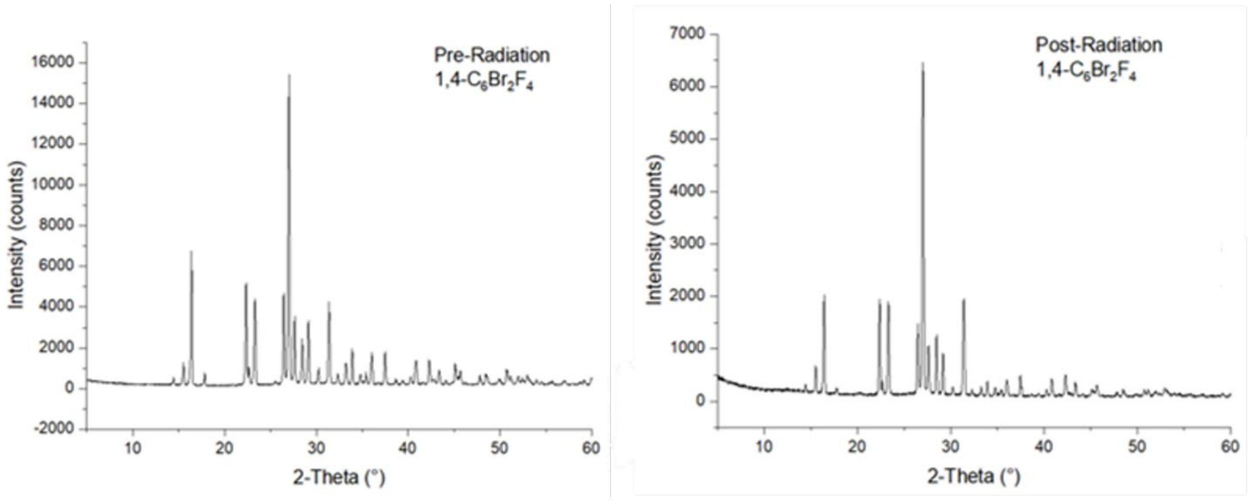

Figure S9. Powder pattern **1,4-$C_6Br_2F_4$** pre- (left) and post-radiation (right) exposure without background subtraction.

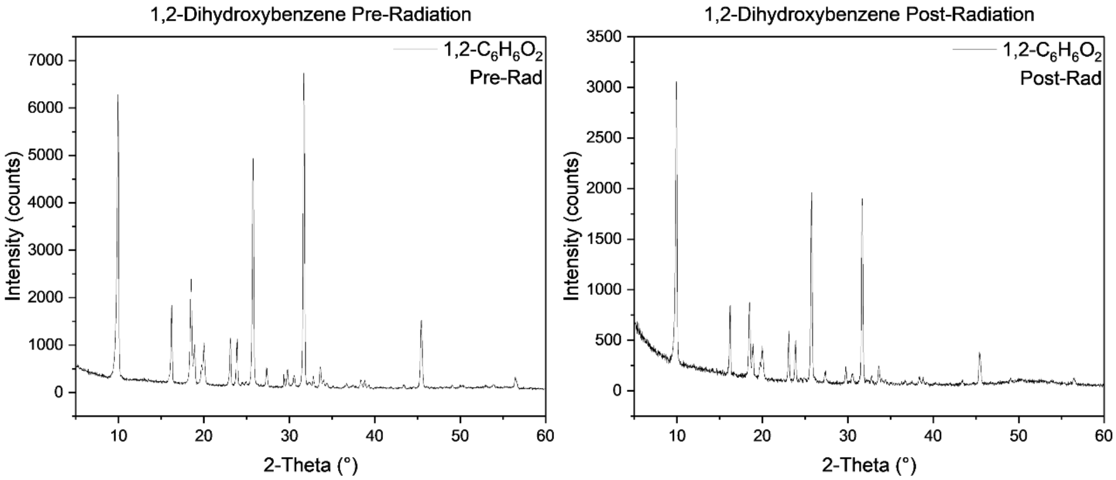

Figure S10. Powder pattern **1,2-$C_6H_6O_2$** pre- (left) and post-radiation (right) exposure without background subtraction.

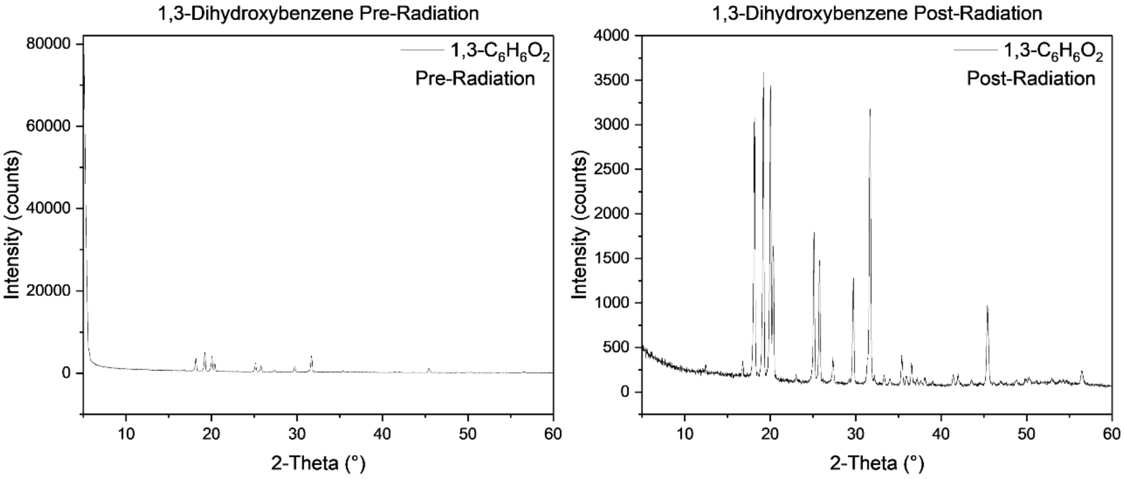

Figure S11. Powder pattern **1,3-$C_6H_6O_2$** pre- (left) and post-radiation (right) exposure without background subtraction.

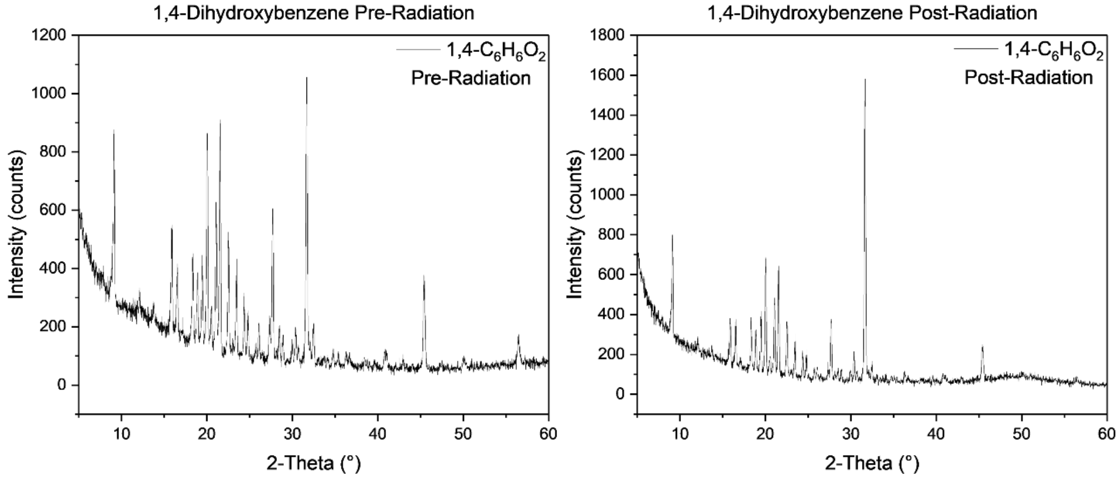

Figure S12. Powder pattern **1,4-$C_6H_6O_2$** pre- (left) and post-radiation (right) exposure without background subtraction.

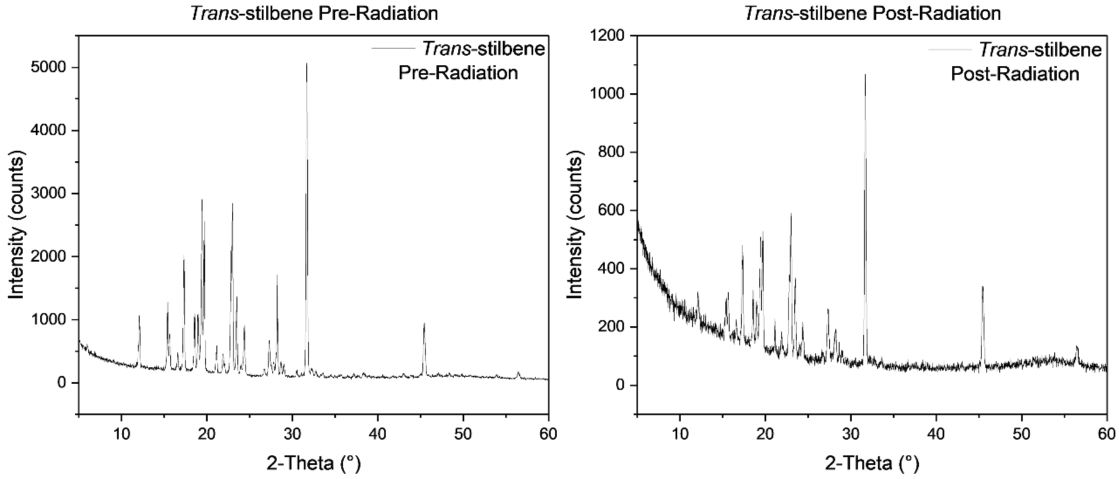

Figure S13. Powder pattern ***trans*-stilbene** pre- (left) and post-radiation (right) exposure without background subtraction.

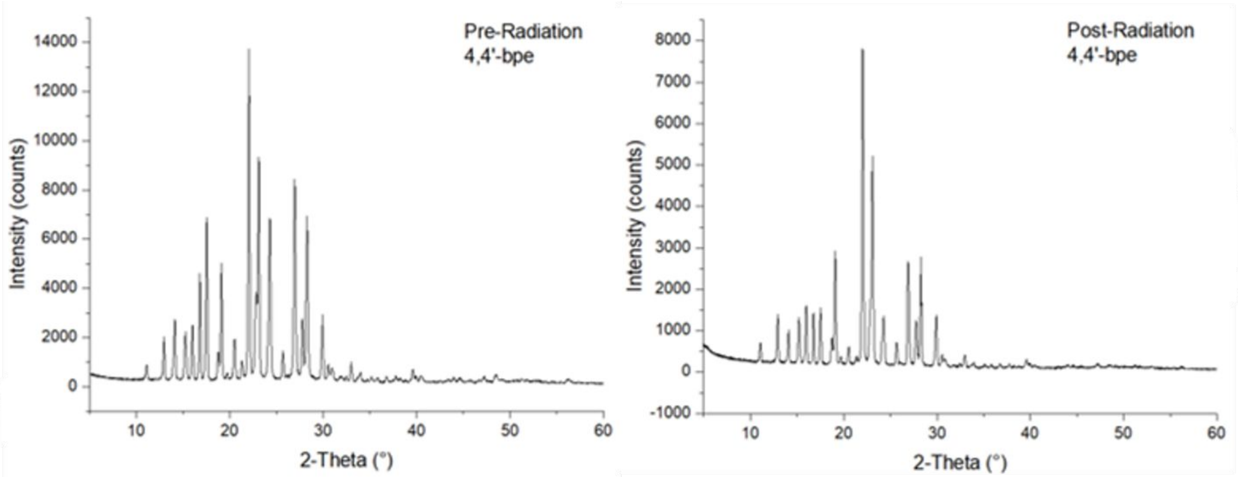

Figure S14. Powder pattern **4,4'-bpe** pre- (left) and post-radiation (right) exposure without background subtraction.

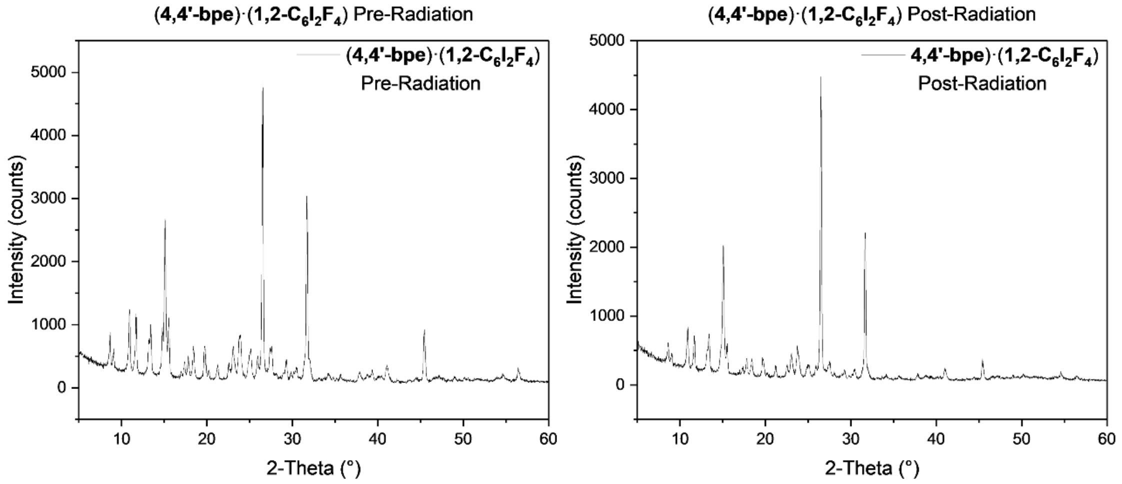

Figure S15. Powder pattern (**4,4’-bpe**)·(**1,2-$C_6I_2F_4$**) pre- (left) and post-radiation (right) exposure without background subtraction.

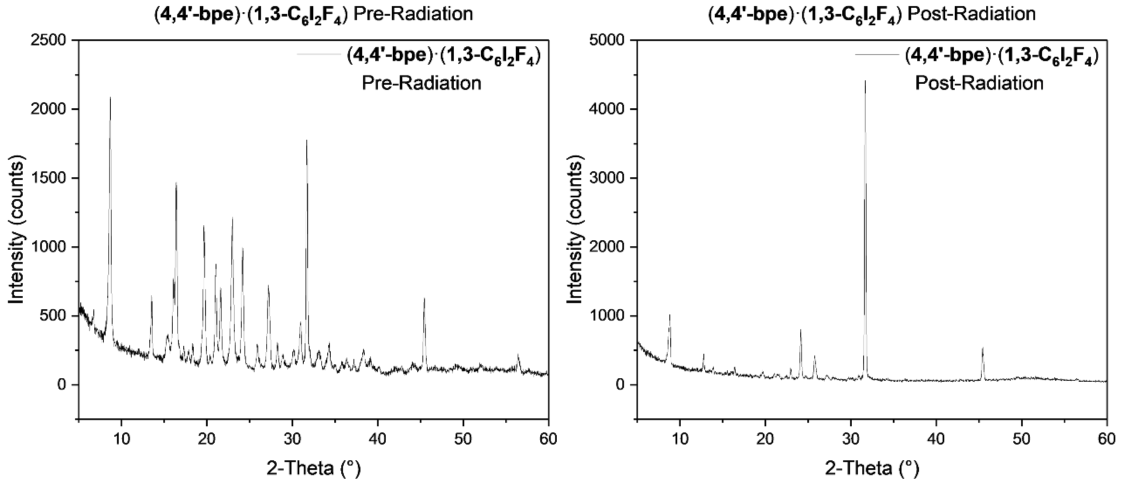

Figure S16. Powder pattern (**4,4’-bpe**)·(**1,3-$C_6I_2F_4$**) pre- (left) and post-radiation (right) exposure without background subtraction.

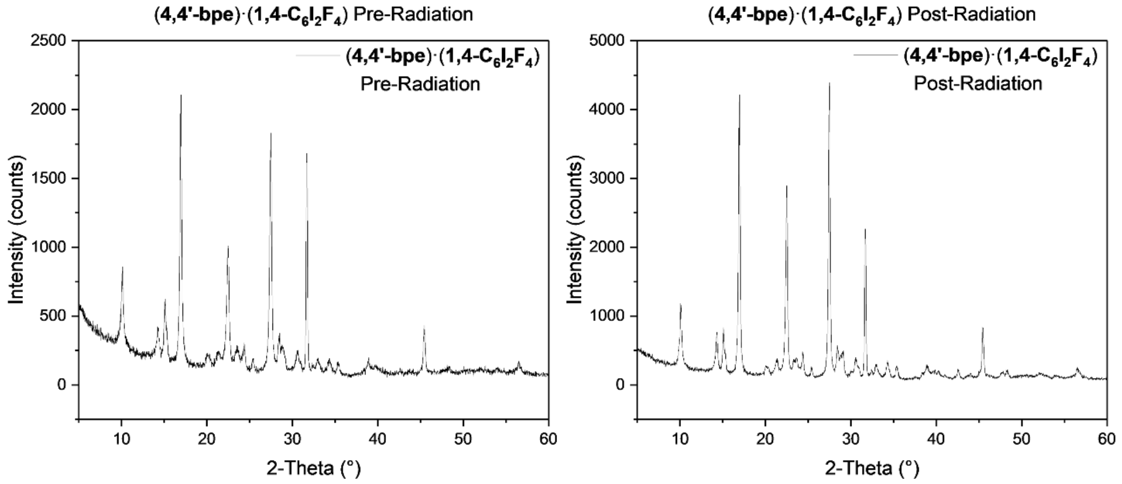

Figure S17. Powder pattern (**4,4'-bpe**)·(**1,4-$C_6I_2F_4$**) pre- (left) and post-radiation (right) exposure without background subtraction.

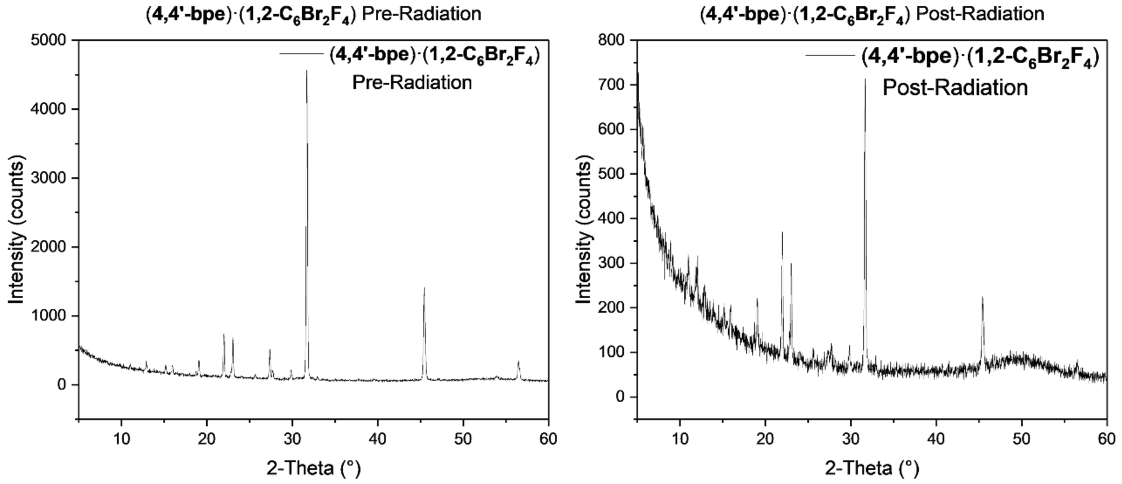

Figure S18. Powder pattern (**4,4'-bpe**)·(**1,2-$C_6Br_2F_4$**) pre- (left) and post-radiation (right) exposure without background subtraction.

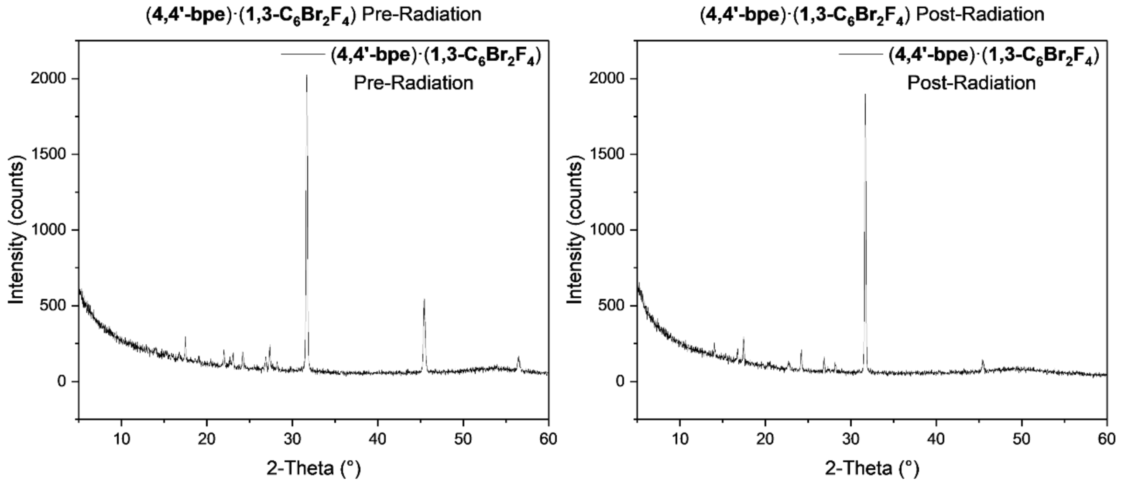

Figure S19. Powder pattern (**4,4'-bpe**)·(**1,3-$C_6Br_2F_4$**) pre- (left) and post-radiation (right) exposure without background subtraction.

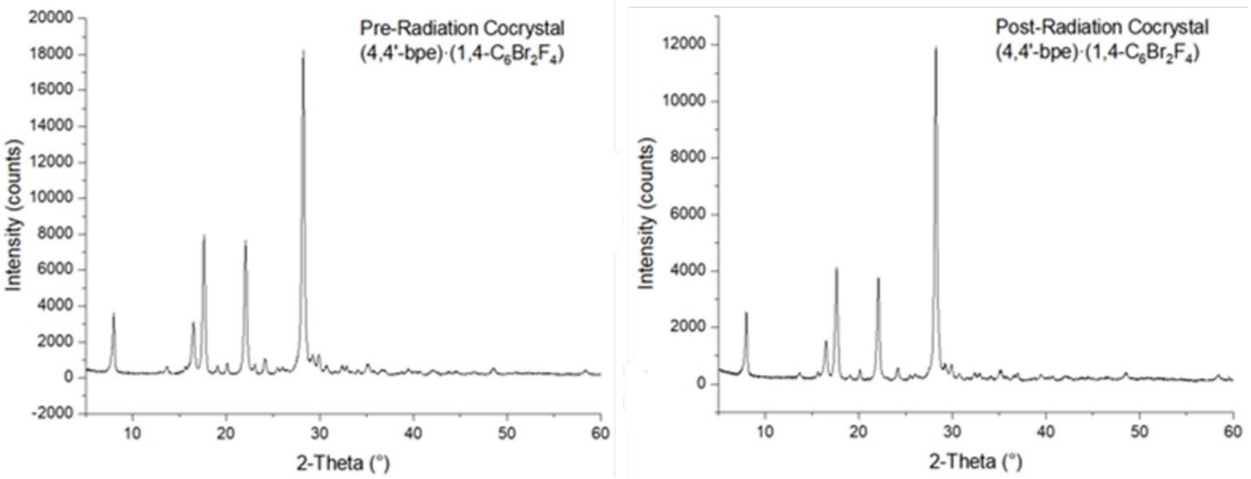

Figure S20. Powder pattern (**4,4'-bpe**)·(**1,4-$C_6Br_2F_4$**) pre- (left) and post-radiation (right) exposure without background subtraction.

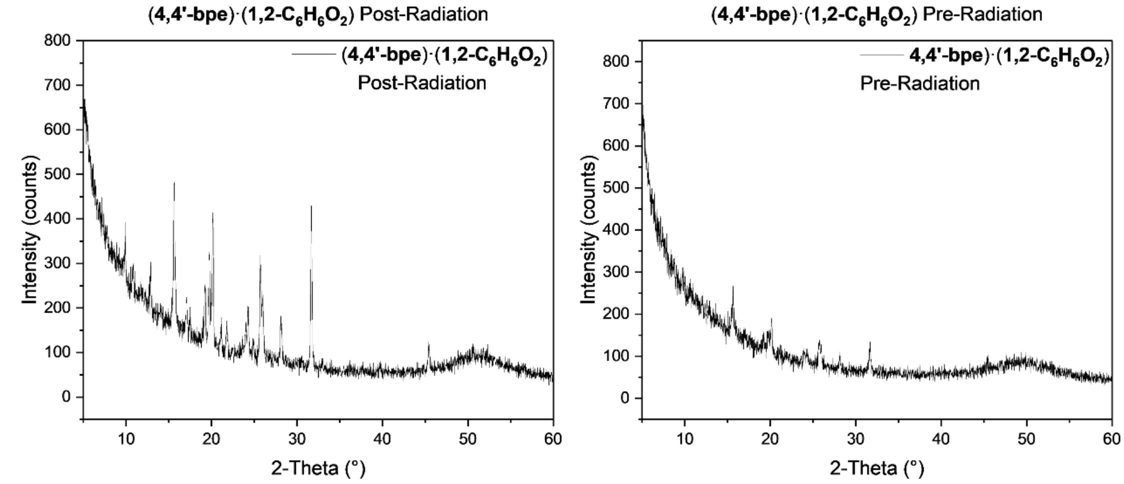

Figure S21. Powder pattern **(4,4'-bpe)·(1,2-$C_6H_6O_2$)** pre- (left) and post-radiation (right) exposure without background subtraction.

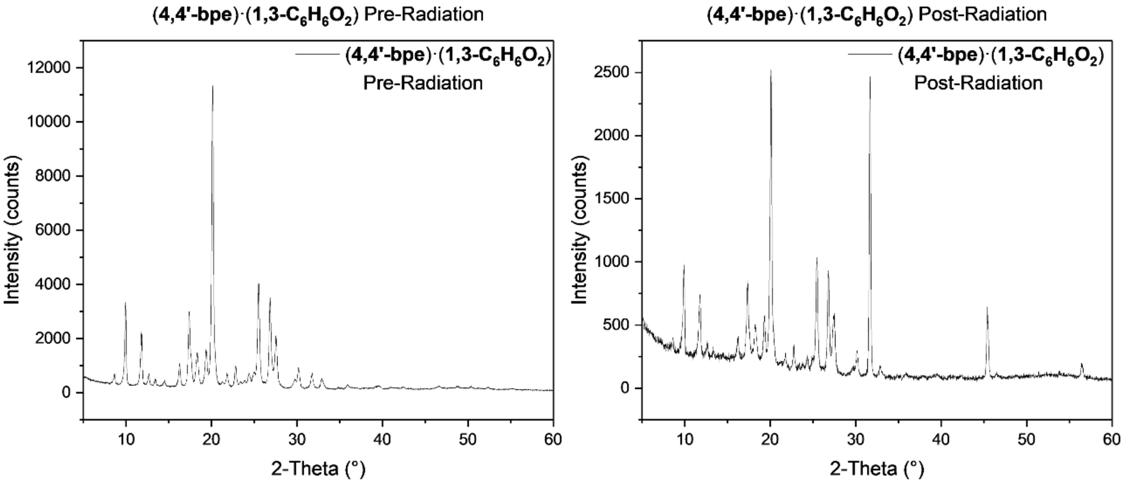

Figure S22. Powder pattern **(4,4'-bpe)·(1,3-$C_6H_6O_2$)** pre- (left) and post-radiation (right) exposure without background subtraction.

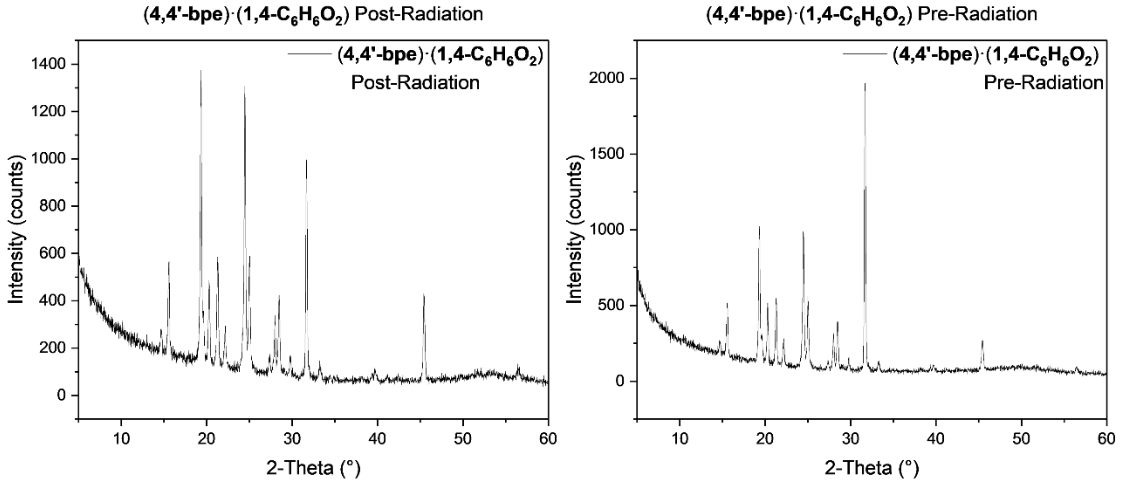

Figure S23. Powder pattern (**4,4'-bpe**)·(**1,4-$C_6H_6O_2$**) pre- (left) and post-radiation (right) exposure without background subtraction.

Powder pattern intensity changes

First, patterns were normalized to the most intense peak of NaCl (2θ = 31.67°). Differences in powder pattern intensities were taken using the five most intense peaks associated with the sample and not the standard, NaCl. Each peak was then divided by the most intense peak associated with NaCl to relate pre- and post-irradiated samples without having to rely on any error that may be associated with sample preparation.

Table S15. Intensity changes in powder patterns for **1,2-$C_6I_2F_4$**.

2-Theta (°)	Pre-radiation Intensity (counts)	Post-radiation Intensity (counts)	Pre-radiation Intensity / NaCl	Post-radiation Intensity / NaCl	Percent Change (%)
23.33	4057	2865	1.153	0.8143	17.22
8.779	1925	1961	0.5471	0.5574	-0.9264
17.57	1733	1294	0.4926	0.3678	14.50
23.94	1696	1232	0.4820	0.3501	15.84
29.26	1689	915.2	0.4801	0.2601	29.71

Table S16. Intensity changes in powder patterns for **1,4-$C_6I_2F_4$***. *Note: This specific powder underwent some change upon exposure to radiation.

2-Theta (°)	Pre-radiation Intensity (counts)	Post-radiation Intensity (counts)	Pre-radiation Intensity / NaCl	Post-radiation Intensity / NaCl	Percent Change (%)
26.20	3888	6.78	1.516	0.02520	99.65
16.09	3116	22.29	1.215	0.02831	98.57
25.39	2265	3.971	0.8837	0.1241	99.64
21.45	1605	1662	0.6262	0.7058	-1.744
22.53	1281	0.552	0.4998	0.2292	99.913

Table S17. Intensity changes in powder patterns for **1,4-$C_6Br_2F_4$**.

2-Theta (°)	Pre-radiation Intensity (counts)	Post-radiation Intensity (counts)	Pre-radiation Intensity / NaCl	Post-radiation Intensity / NaCl	Percent Change (%)
16.40	6088	4377	1.442	1.036	16.34
22.34	4893	4118	1.159	0.9755	8.600
26.99	14280	13760	3.383	3.259	1.854
26.44	4364	3033	1.033	0.7185	17.99
23.29	4178	3984	0.9898	0.9438	2.376

Table S18. Intensity changes in powder patterns for **1,2-$C_6H_6O_2$**.

2-Theta (°)	Pre-radiation Intensity (counts)	Post-radiation Intensity (counts)	Pre-radiation Intensity / NaCl	Post-radiation Intensity / NaCl	Percent Change (%)
9.953	2752.8	5803.9	1.525	0.8930	26.16
25.75	1827.2	4715.8	1.012	0.7255	16.52
18.49	708.1	2083.3	0.3925	0.3205	10.09
16.22	679.5	1603.4	0.3766	0.2467	20.84
23.09	457.4	968.8	0.2535	0.1490	25.95

Table S19. Intensity changes in powder patterns for **1,3-$C_6H_6O_2$**.

2-Theta (°)	Pre-radiation Intensity (counts)	Post-radiation Intensity (counts)	Pre-radiation Intensity / NaCl	Post-radiation Intensity / NaCl	Percent Change (%)
20.09	34284.6	9711.7	16.46	0.5784	93.21
19.27	25735.6	8205.1	12.36	0.4886	92.39
18.21	23537.2	6568.3	11.30	0.3911	93.31
20.4	14528.2	3970.6	6.979	0.2364	93.44
25.20	13015	4153.6	6.252	0.2473	92.38

Table S20. Intensity changes in powder patterns for **1,4-$C_6H_6O_2$**.

2-Theta (°)	Pre-radiation Intensity (counts)	Post-radiation Intensity (counts)	Pre-radiation Intensity / NaCl	Post-radiation Intensity / NaCl	Percent Change (%)
21.54	750	509.3	0.8119	0.3461	40.22
20.04	722.8	547	0.7825	0.3717	35.58
9.132	526.3	474.9	0.5697	0.3227	27.67
21.08	479.4	355.4	0.5190	0.2415	36.48
22.53	399.2	263.8	0.4321	0.1792	41.36

Table S21. Intensity changes in powder patterns for ***trans*-stilbene**.

2-Theta (°)	Pre-radiation Intensity (counts)	Post-radiation Intensity (counts)	Pre-radiation Intensity / NaCl	Post-radiation Intensity / NaCl	Percent Change (%)
23.01	2480.5	464.7	0.5073	0.4861	2.132
19.68	2220.5	385.1	0.4541	0.4028	5.983
19.41	2588.3	356.7	0.5293	0.3731	17.30
17.34	1778	304.8	0.3636	0.3188	6.561
28.23	1153.3	95.8	0.2358	0.1002	40.36

Table S22. Intensity changes in powder patterns for **4,4'-bpe**.

2-Theta (°)	Pre-radiation Intensity (counts)	Post-radiation Intensity (counts)	Pre-radiation Intensity / NaCl	Post-radiation Intensity / NaCl	Percent Change (%)
17.45	2393.8	1933.9	0.8271	0.7807	2.888
24.19	2512.7	1989.6	0.8682	0.8031	3.891
26.87	3026.3	2543.4	1.045	1.026	0.9143
28.20	1916.9	1560.4	0.6623	0.6299	2.510
16.94	1613.1	1253.5	0.5573	0.5060	4.829

Table S23. Intensity changes in powder patterns for (**4,4'-bpe**)·(**1,2-$C_6I_2F_4$**).

2-Theta (°)	Pre-radiation Intensity (counts)	Post-radiation Intensity (counts)	Pre-radiation Intensity / NaCl	Post-radiation Intensity / NaCl	Percent Change (%)
26.52	4492.9	4132.7	1.596	1.988	-10.94
15.10	2260.4	1755.2	0.8031	0.8445	-2.515
10.94	946.6	541.1	0.3363	0.2603	12.73
11.70	868.1	444.7	0.3084	0.2139	18.08
13.40	719.9	496.4	0.2557	0.2388	3.421

Table S24. Intensity changes in powder patterns for (**4,4'-bpe**)·(**1,3-$C_6I_2F_4$**).

2-Theta (°)	Pre-radiation Intensity (counts)	Post-radiation Intensity (counts)	Pre-radiation Intensity / NaCl	Post-radiation Intensity / NaCl	Percent Change (%)
8.695	1721.6	4132.7	1.073	0.9591	5.6150
16.41	1233.9	1755.2	0.7692	0.4073	30.75
19.64	958.5	245.5	0.5975	0.05697	82.58
22.97	972.7	269.5	0.6063	0.06254	81.29
24.18	808.1	662.1	0.5037	0.1536	53.25

Table S25. Intensity changes in powder patterns for (**4,4'-bpe**)·(**1,4-$C_6I_2F_4$**).

2-Theta (°)	Pre-radiation Intensity (counts)	Post-radiation Intensity (counts)	Pre-radiation Intensity / NaCl	Post-radiation Intensity / NaCl	Percent Change (%)
16.94	3912.6	1860.1	2.526	0.8759	48.512
22.50	2580.7	748.1	1.666	0.3522	65.09
10.10	843.1	487.7	0.5444	0.2296	40.66
15.09	555.4	377.7	0.3586	0.1778	33.69
14.26	509	164.1	0.3286	0.07727	61.93

Table S26. Intensity changes in powder patterns for (**4,4'-bpe**)·(**1,2-$C_6Br_2F_4$**).

2-Theta (°)	Pre-radiation Intensity (counts)	Post-radiation Intensity (counts)	Pre-radiation Intensity / NaCl	Post-radiation Intensity / NaCl	Percent Change (%)
21.97	264.9	626.7	0.4169	0.1405	49.58
23.00	207.7	576.3	0.3268	0.1292	43.33
19.04	107.5	227.7	0.1691	0.05105	53.63
12.91	56.2	139.4	0.08845	0.03125	47.77
15.96	55.7	132.9	0.08766	0.02979	49.26

Table S27. Intensity changes in powder patterns for (**4,4'-bpe**)·(**1,3-$C_6Br_2F_4$**).

2-Theta (°)	Pre-radiation Intensity (counts)	Post-radiation Intensity (counts)	Pre-radiation Intensity / NaCl	Post-radiation Intensity / NaCl	Percent Change (%)
17.47	129.6	156.4	0.06712	0.08853	-13.76
24.19	108.7	125.8	0.05629	0.07121	-11.69
23.05	93.2	47.8	0.04827	0.02705	28.15
21.99	92.7	30.8	0.04801	0.01743	46.72
26.89	79.9	88.7	0.04138	0.05021	-9.641

Table S28. Intensity changes in powder patterns for (**4,4'-bpe**)·(**1,4-$C_6Br_2F_4$**).

2-Theta (°)	Pre-radiation Intensity (counts)	Post-radiation Intensity (counts)	Pre-radiation Intensity / NaCl	Post-radiation Intensity / NaCl	Percent Change (%)
28.20	5203.9	4719.9	1.293	1.566	-9.525
17.60	5072.3	3181.3	1.260	1.055	8.865
22.05	3812	2636	0.9476	0.8746	4.006
7.965	3058.7	1854.7	0.7603	0.6153	10.53
16.48	1278.6	947.5	0.3178	0.3143	0.5487

Table S29. Intensity changes in powder patterns for (**4,4'-bpe**)·(**1,2-$C_6H_6O_2$**).

2-Theta (°)	Pre-radiation Intensity (counts)	Post-radiation Intensity (counts)	Pre-radiation Intensity / NaCl	Post-radiation Intensity / NaCl	Percent Change (%)
15.61	101.2	316.8	1.421	0.9353	20.62
20.15	82.7	281.2	1.16	0.8302	16.63
25.69	54.1	204.5	0.7598	0.6037	11.44
19.71	50.6	188.4	0.7106	0.5562	12.18
25.99	51.6	123.9	0.7247	0.3658	32.91

Table S30. Intensity changes in powder patterns for (**4,4'-bpe**)·(**1,3-$C_6H_6O_2$**).

2-Theta (°)	Pre-radiation Intensity (counts)	Post-radiation Intensity (counts)	Pre-radiation Intensity / NaCl	Post-radiation Intensity / NaCl	Percent Change (%)
20.10	10625	567.7	20.35	2.346	79.32
26.8	2906.7	156.8	5.567	0.6482	79.14
25.51	3555.5	202.3	6.809	0.8362	78.12
9.945	2990.3	147.4	5.727	0.6093	80.76
17.37	2612.2	99.1	5.003	0.4096	84.86

Table S31. Intensity changes in powder patterns for (**4,4'-bpe**)·(**1,4-$C_6H_6O_2$**).

2-Theta (°)	Pre-radiation Intensity (counts)	Post-radiation Intensity (counts)	Pre-radiation Intensity / NaCl	Post-radiation Intensity / NaCl	Percent Change (%)
20.29	10800	10250	21.31	20.22	2.612
25.54	3675	3447	7.251	6.801	3.201
17.36	2614	2094	5.157	4.131	11.05
9.968	2849	2557	5.621	5.045	5.401
11.84	1683	1660	3.321	3.275	0.6880

Raman Spectroscopy

*Note: Raman were not fit for the (**4,4'-bpe**)□(**1,*n*-$C_6H_6O_2$**) (where n = 2, 3 or 4) as the pre- and post-irradiated samples both could not withstand any laser power (1-15 mW) and burned upon exposure to the laser.

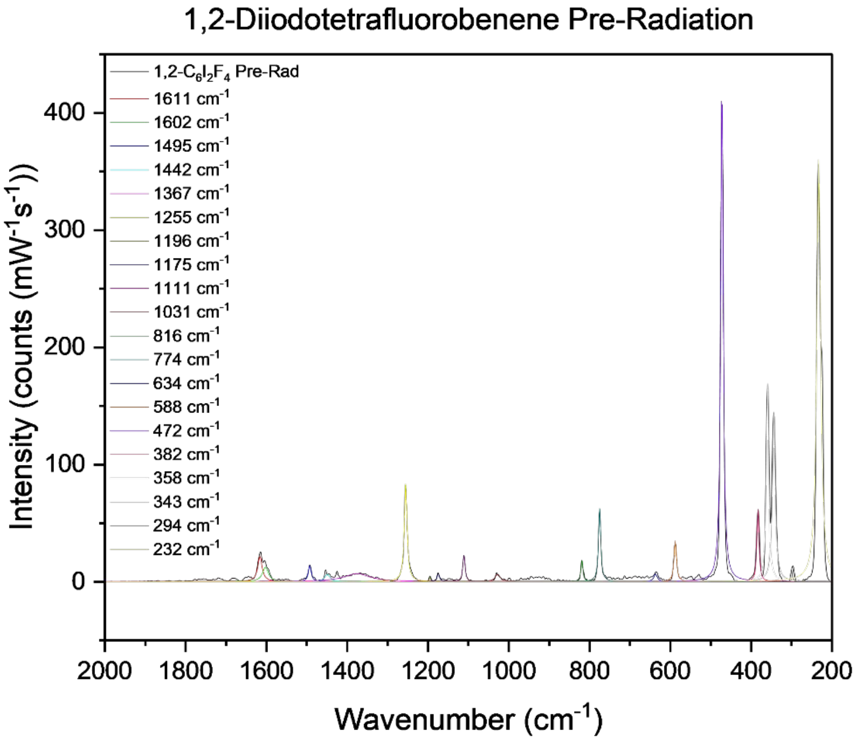

Raman Shift (cm-1)	Assignment (Symmetry)
232	a_1v_{10}
294	a_1v_9
343	b_2v_{26}
358	a_1v_8
382	b_1v_{16}
472	a_1v_7
588	a_2v_{12}
634	b_1v_{15}
774	a_1v_6
816	b_2v_{23}
1031	b_2v_{22}
1111	a_1v_5
1175	a_1v_5
1196	a_1v_5
1255	a_1v_4
1367	b_2v_{21}
1442	a_1v_3
1495	a_1v_2
1602	b_2v_{19}
1611	a_1v_1

Figure S24. Fitted Raman spectrum of **1,2-$C_6I_2F_4$** pre-radiation.[1]

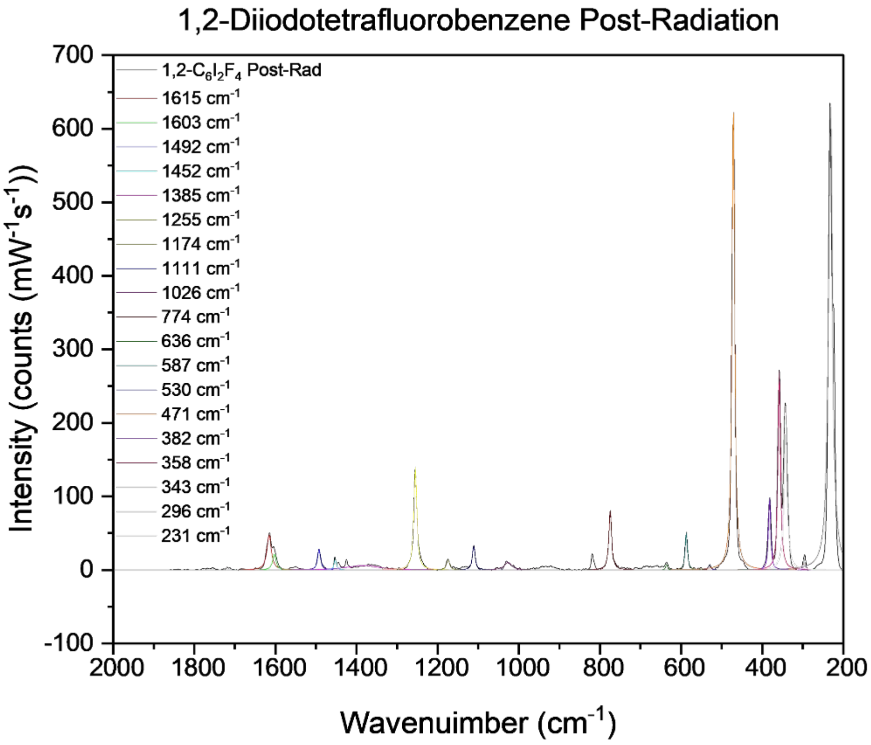

Raman Shift (cm-1)	Assignment (Symmetry)
231	$a_1\nu_{10}$
296	$a_1\nu_9$
343	$b_2\nu_{26}$
358	$a_1\nu_8$
382	$b_1\nu_{16}$
471	$a_1\nu_7$
530	$b_2\nu_{25}$
587	$a_2\nu_{12}$
636	$b_1\nu_{15}$
774	$a_1\nu_6$
1026	$b_2\nu_{22}$
1111	$a_1\nu_5$
1174	$a_1\nu_5$
1255	$a_1\nu_4$
1385	$b_2\nu_{21}$
1452	$a_1\nu_3$
1492	$a_1\nu_2$
1603	$b_2\nu_{19}$
1615	$a_1\nu_1$

Figure S25. Fitted Raman spectrum of **1,2-$C_6I_2F_4$** post-radiation.[1]

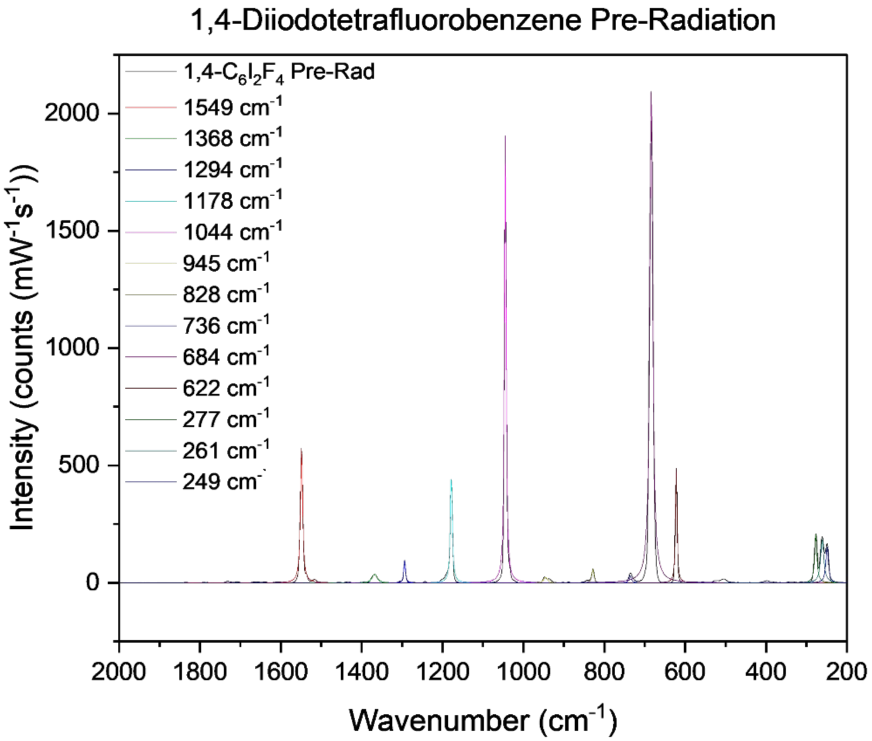

Raman Shift (cm-1)	Assignment (Symmetry)
249	$b_{1u}v_{22}$
261	$b_{1u}v_{22}$
277	$a_{g}v_{5}$
622	$b_{3u}v_{28}$
684	$a_{u}v_{16}$
736	$b_{1u}v_{20}$
828	$a_{g}v_{3}$
945	$b_{2u}v_{25}$
1044	$b_{3g}v_{12}$
1178	$b_{3g}v_{12}$
1294	$b_{1u}v_{19}$
1368	$a_{g}v_{2}$
1549	$b_{3g}v_{11}$

Figure S26. Fitted Raman spectrum of **1,4-$C_6I_2F_4$** pre-radiation.[2]

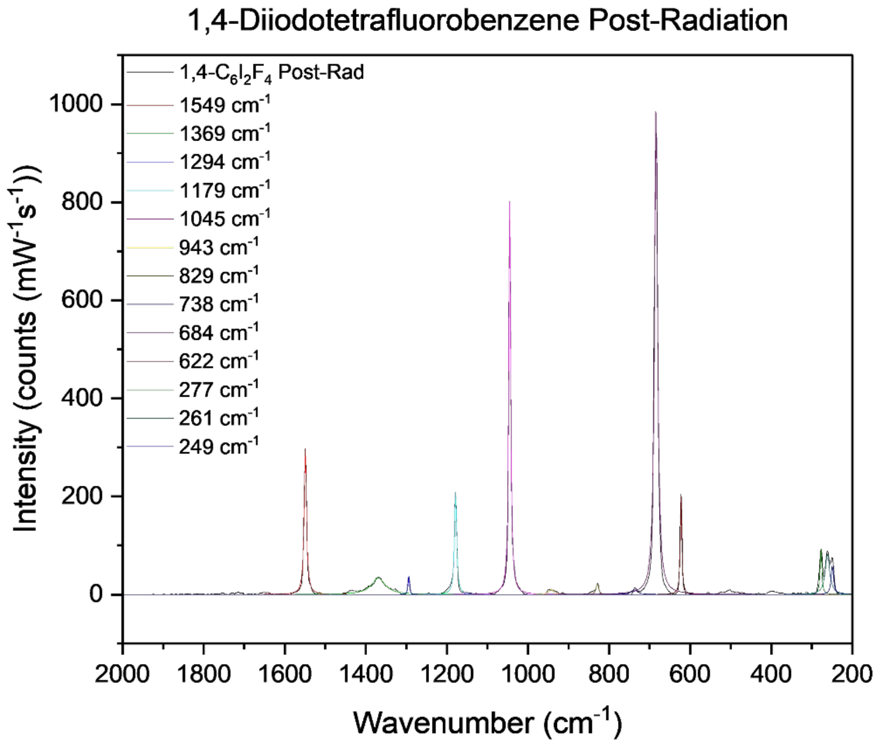

Raman Shift (cm-1)	Assignment (Symmetry)
249	$b_{1u}\nu_{22}$
261	$b_{1u}\nu_{22}$
277	$a_g\nu_5$
622	$b_{3u}\nu_{28}$
684	$a_u\nu_{16}$
738	$b_{1u}\nu_{20}$
829	$a_g\nu_3$
943	$b_{2u}\nu_{25}$
1045	$b_{3g}\nu_{12}$
1179	$b_{3g}\nu_{12}$
1294	$b_{1u}\nu_{19}$
1369	$a_g\nu_2$
1549	$b_{3g}\nu_{11}$

Figure S27. Fitted Raman spectrum of **1,4-$C_6I_2F_4$** post-radiation.[2]

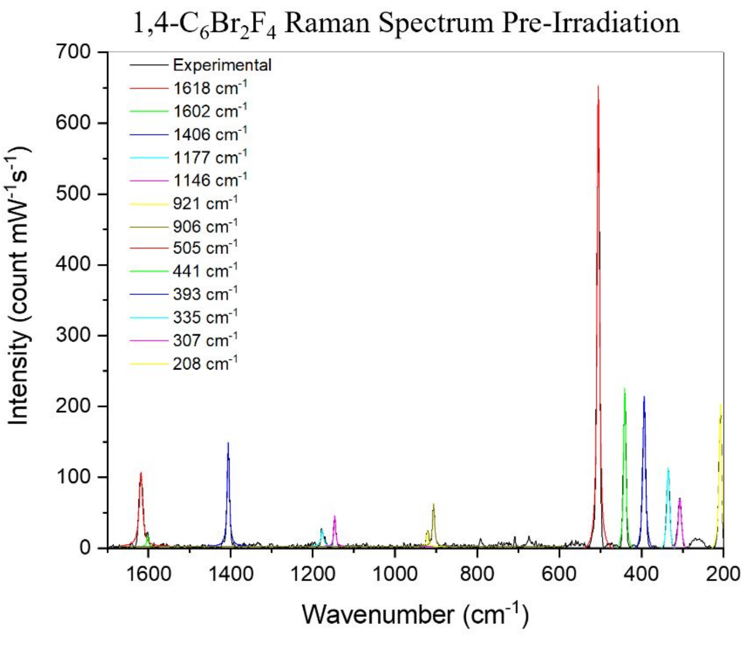

Raman Shift (cm-1)	Assignment (Symmetry)
208	$a_g\,\nu_6$
307	$a_g\,\nu_5$
335	$b_{2g}\,\nu_9$
393	$b_{1g}\,\nu_7$
441	$b_{3g}\,\nu_{14}$
505	$a_g\,\nu_4$
906	$a_g\,\nu_3$
921	$a_g\,\nu_3$
1146	$b_{3g}\,\nu_{12}$
1177	$b_{3g}\,\nu_{12}$
1406	$a_g\,\nu_2$
1602	$a_g\,\nu_1$
1618	$a_g\,\nu_1$

Figure S28. Fitted Raman spectrum of **1,4-$C_6Br_2F_4$** pre-radiation.[3]

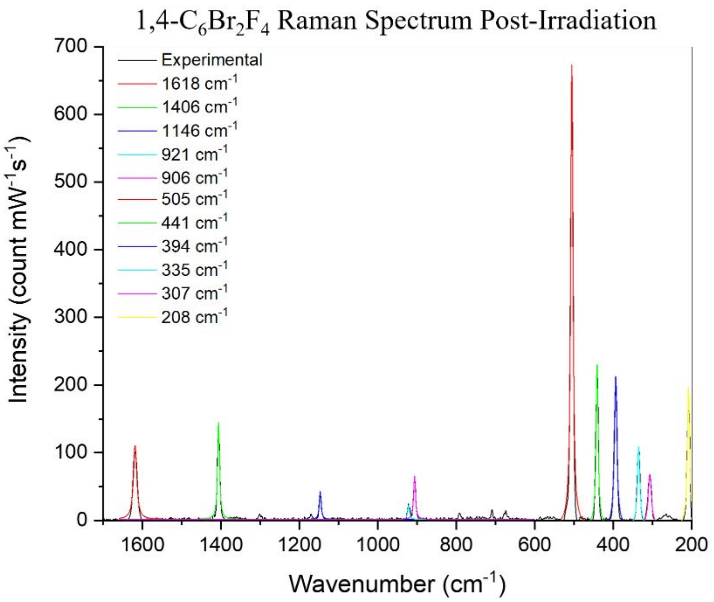

Raman Shift (cm-1)	Assignment (Symmetry)
208	$a_g\, \nu_6$
307	$a_g\, \nu_5$
335	$b_{2g}\, \nu_9$
394	$b_{1g}\, \nu_7$
441	$b_{3g}\, \nu_{14}$
505	$a_g\, \nu_4$
906	$a_g\, \nu_3$
921	$a_g\, \nu_3$
1146	$b_{3g}\, \nu_{12}$
1406	$a_g\, \nu_2$
1618	$a_g\, \nu_1$

Figure S29. Fitted Raman spectrum of **1,4-$C_6Br_2F_4$** post-radiation.[3]

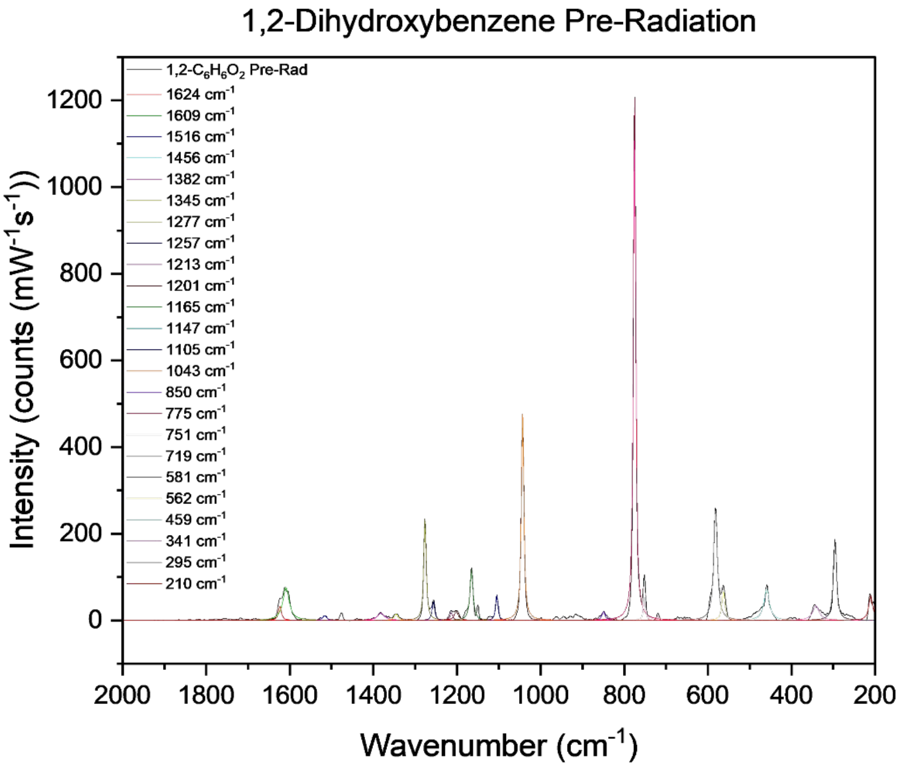

Raman Shift (cm-1)	Assignment (Symmetry)
210	$b_1\nu_2$
295	$a_1\nu_3$
341	$b_1\nu_4$
459	$b_1\nu_5$
562	$a_1\nu_7$
581	$a_2\nu_8$
719	$a_1\nu_8$
751	$b_1\nu_9$
775	$a_1\nu_{10}$
850	$b_1\nu_{11}$
1043	$a_1\nu_{14}$
1147	$b_2\nu_{15}$
1165	$a_1\nu_{16}$
1201	$b_2\nu_{17}$
1213	$b_2\nu_{17}$
1257	$b_2\nu_{18}$
1277	$a_1\nu_{19}$
1345	$a_1\nu_{20}$
1382	$a_1\nu_{20}$
1456	$b_2\nu_{21}$
1516	$b_2\nu_{22}$
1609	$a_1\nu_{23}$
1624	$a_1\nu_{25}$

Figure S30. Fitted Raman spectrum of **1,2-$C_6H_6O_2$** pre-radiation.[4]

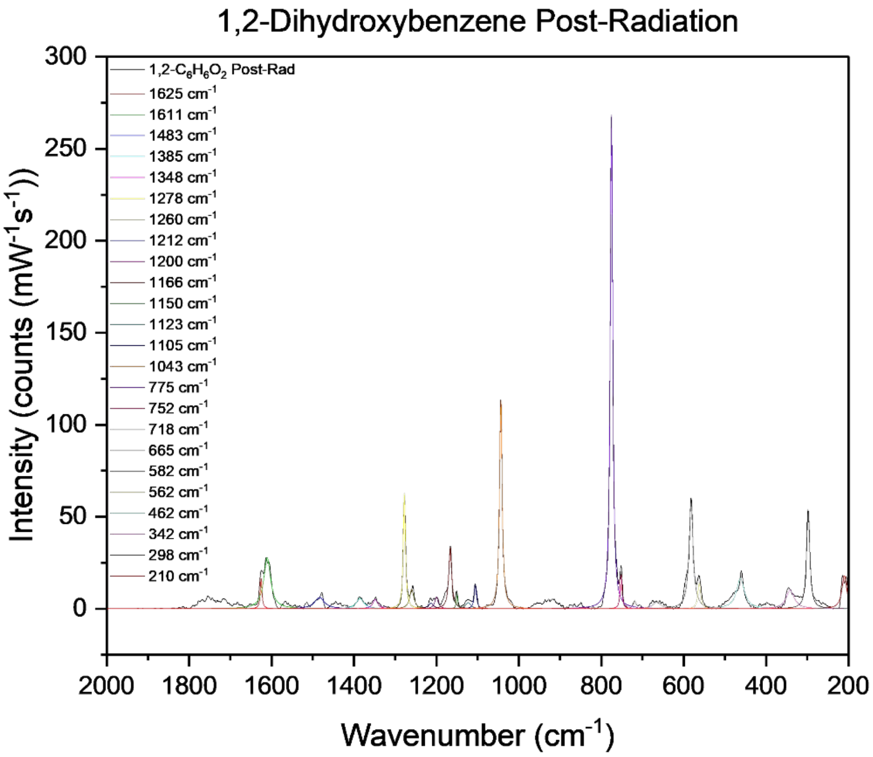

Raman Shift (cm-1)	Assignment (Symmetry)
210	b_1v_2
298	a_1v_3
342	b_1v_4
462	b_1v_5
562	a_1v_7
582	a_2v_8
718	a_1v_8
752	b_1v_9
775	a_1v_{10}
1043	a_1v_{14}
1150	b_2v_{15}
1166	a_1v_{16}
1200	b_2v_{17}
1212	b_2v_{17}
1260	b_2v_{18}
1278	a_1v_{19}
1348	a_1v_{20}
1385	a_1v_{20}
1483	b_2v_{21}
1611	a_1v_{23}
1625	a_1v_{25}

Figure S31. Fitted Raman spectrum of **1,2-$C_6H_6O_2$** post-radiation.[4]

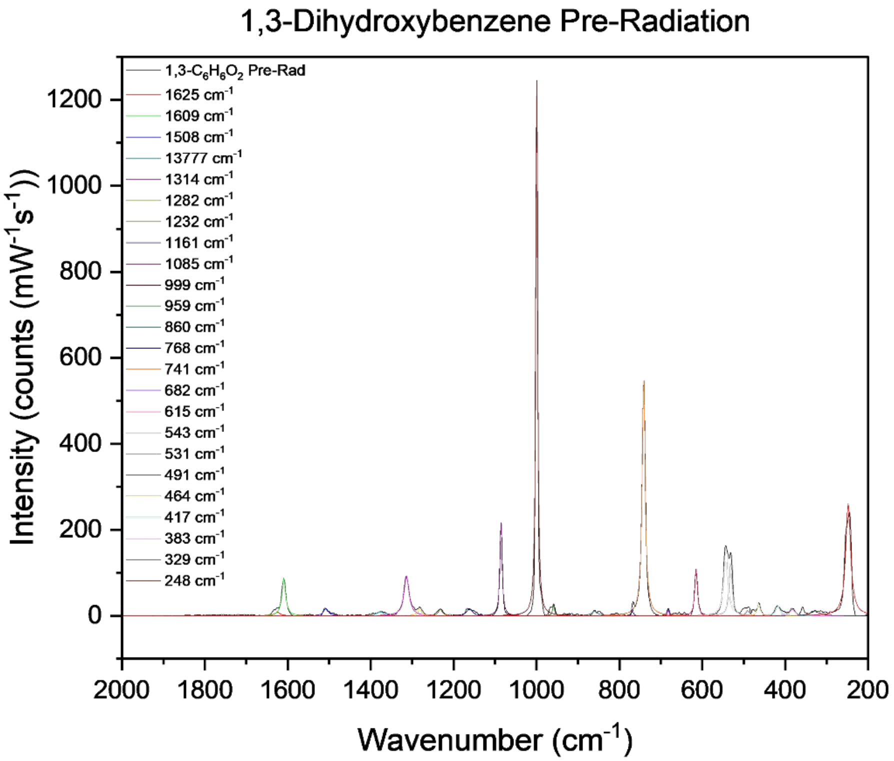

Raman Shift (cm-1)	Assignment (Symmetry)
248	$a_2\nu_{17}$
329	$a_1\nu_{13}$
383	$a_1\nu_{13}$
417	$a_1\nu_{13}$
464	$b_1\nu_{29}$
491	$b_1\nu_{29}$
531	$b_1\nu_{28}$
543	$a_1\nu_{12}$
615	$a_2\nu_{15}$
682	$b_2\nu_{33}$
741	$a_1\nu_{11}$
768	$b_2\nu_{32}$
860	$b_2\nu_{31}$
959	$b_1\nu_{27}$
999	$a_1\nu_{10}$
1085	$a_1\nu_9$
1161	$a_1\nu_8$
1232	$b_1\nu_{24}$
1282	$a_1\nu_7$
1314	$b_1\nu_{23}$
1377	$b_1\nu_{22}$
1508	$a_1\nu_6$
1609	$a_1\nu_5$
1625	$b_1\nu_{20}$

Figure S32. Fitted Raman spectrum of **1,3-$C_6H_6O_2$** pre-radiation.[5]

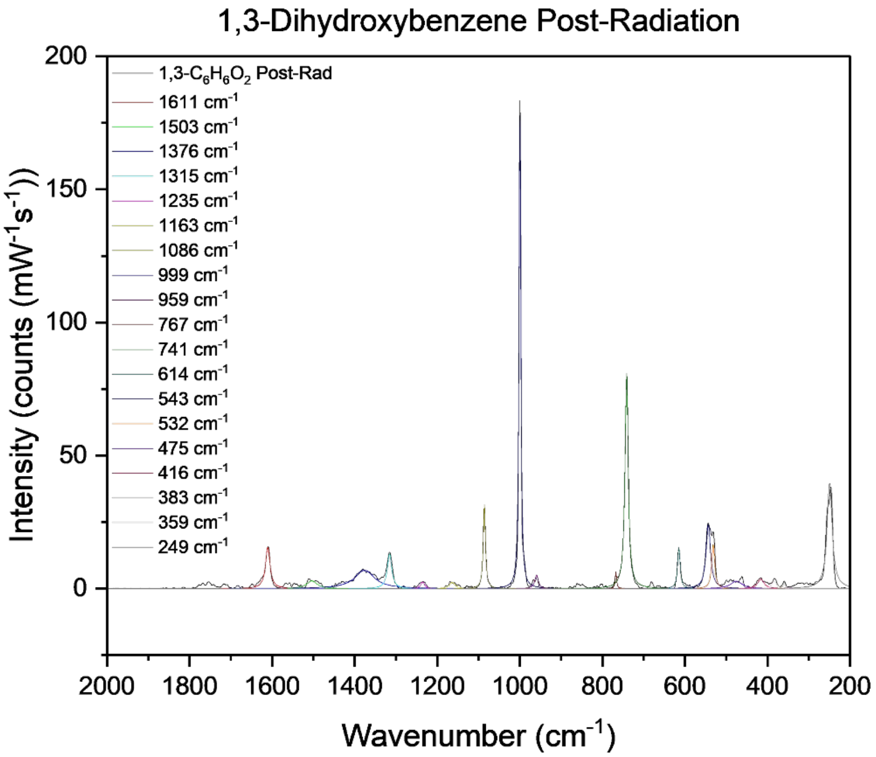

Raman Shift (cm-1)	Assignment (Symmetry)
249	$a_2\nu_{17}$
359	$a_1\nu_{13}$
383	$a_1\nu_{13}$
416	$a_1\nu_{13}$
475	$b_1\nu_{29}$
532	$b_1\nu_{28}$
543	$a_1\nu_{12}$
614	$a_2\nu_{15}$
741	$a_1\nu_{11}$
767	$b_2\nu_{32}$
959	$b_1\nu_{27}$
999	$a_1\nu_{10}$
1086	$a_1\nu_9$
1163	$a_1\nu_8$
1235	$b_1\nu_{24}$
1315	$b_1\nu_{23}$
1376	$b_1\nu_{22}$
1503	$a_1\nu_6$
1625	$b_1\nu_{20}$

Figure S33. Fitted Raman spectrum of **1,3-$C_6H_6O_2$** post-radiation.[5]

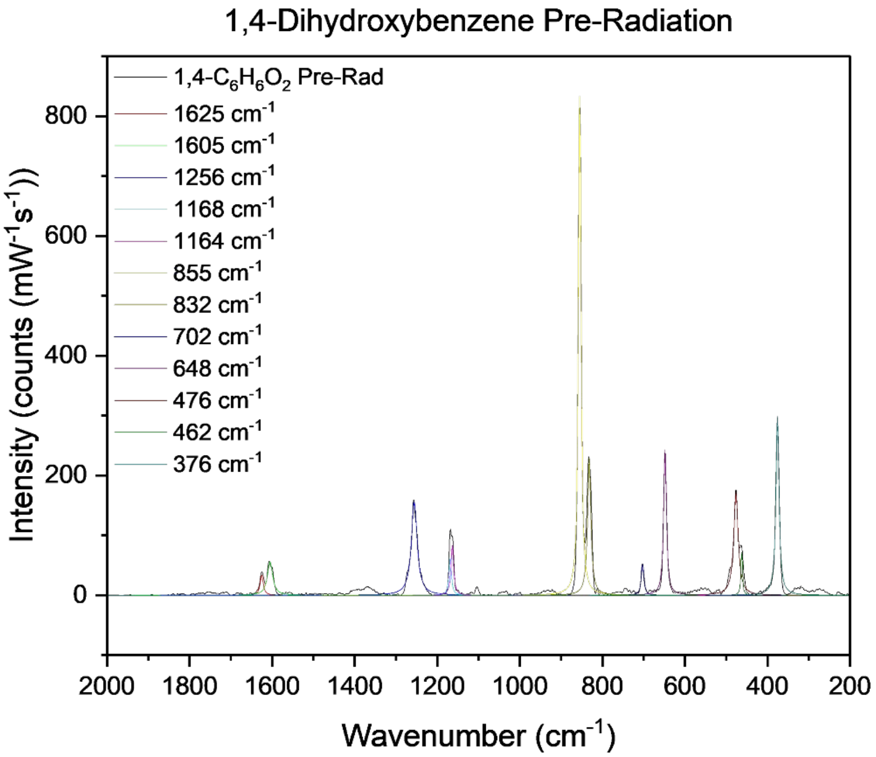

Raman Shift (cm-1)	Assignment (Symmetry)
376	$b_g\nu_5$
462	$a_g\nu_6$
476	$a_g\nu_7$
648	$a_g\nu_{10}$
702	$b_g\nu_{11}$
832	$b_g\nu_{13}$
855	$a_g\nu_{14}$
1164	$a_g\nu_{16}$
1168	$a_g\nu_{16}$
1256	$a_g\nu_{18}$
1605	$a_g\nu_{20}$
1625	$a_g\nu_{21}$

Figure S34. Fitted Raman spectrum of **1,4-$C_6H_6O_2$** pre-radiation.[6]

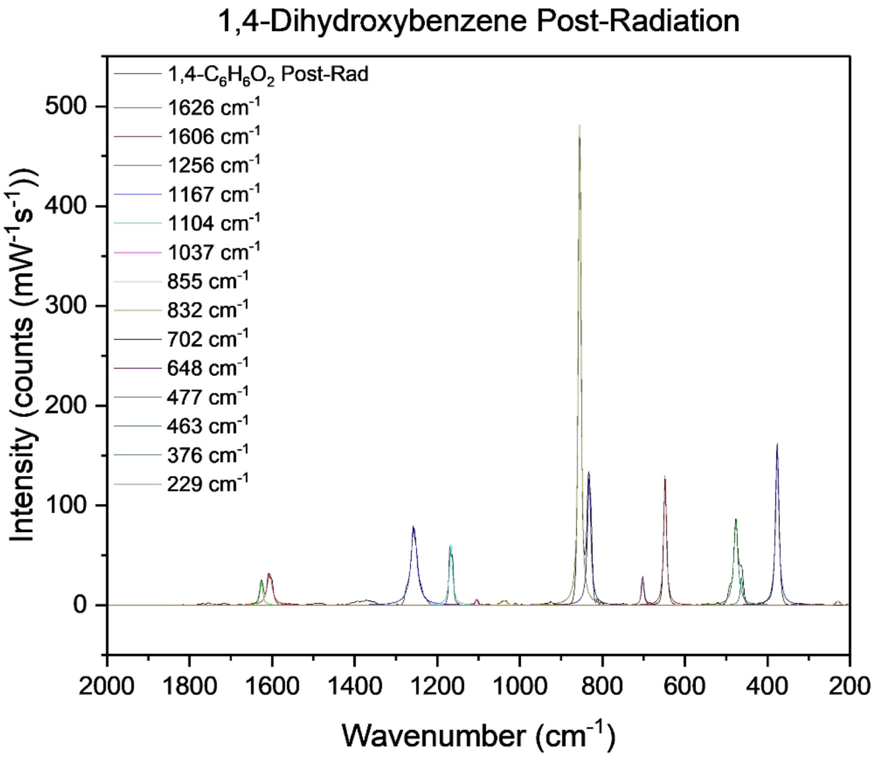

Raman Shift (cm-1)	Assignment (Symmetry)
229	$b_g\nu_5$
376	$b_g\nu_6$
463	$a_g\nu_7$
477	$a_g\nu_8$
648	$a_g\nu_{11}$
702	$b_g\nu_{12}$
832	$b_g\nu_{14}$
855	$a_g\nu_{15}$
1037	$b_g\nu_{16}$
1104	$b_g\nu_{16}$
1167	$a_g\nu_{17}$
1256	$a_g\nu_{19}$
1606	$a_g\nu_{21}$
1626	$a_g\nu_{22}$

Figure S35. Fitted Raman spectrum of **1,4-$C_6H_6O_2$** post-radiation.[6]

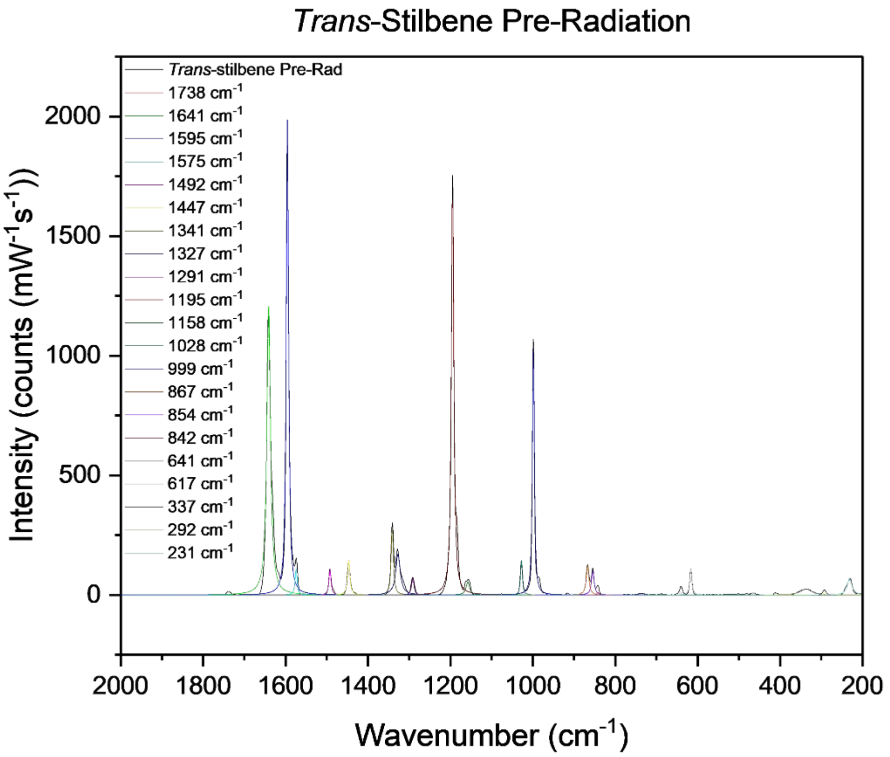

Raman Shift (cm-1)	Assignment (Symmetry)
231	$a_g\nu_{25}$
292	$a_g\nu_{24}$
337	---
617	$a_g\nu_{23}$
641	$a_g\nu_{22}$
842	$a_g\nu_{21}$
854	$a_g\nu_{21}$
867	$a_g\nu_{21}$
999	$a_g\nu_{20}$
1028	$a_g\nu_{19}$
1158	$a_g\nu_{17}$
1195	$a_g\nu_{15}$
1291	$a_g\nu_{14}$
1327	$a_g\nu_{13}$
1341	$a_g\nu_{12}$
1447	$a_g\nu_{11}$
1492	$a_g\nu_{10}$
1575	$a_g\nu_9$
1595	$a_g\nu_8$
1641	$a_g\nu_7$
1738	---

Figure S36. Fitted Raman spectrum of ***trans*-stilbene** pre-radiation.[7]

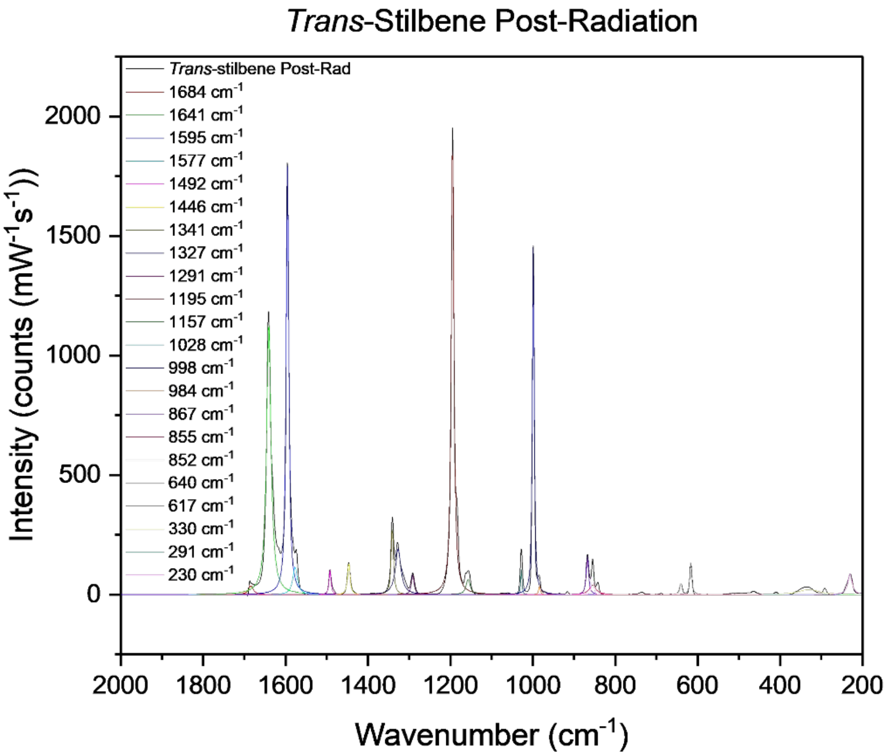

Raman Shift (cm-1)	Assignment (Symmetry)
230	$a_g\nu_{25}$
291	$a_g\nu_{24}$
330	---
617	$a_g\nu_{23}$
640	$a_g\nu_{22}$
852	$a_g\nu_{21}$
855	$a_g\nu_{21}$
867	$a_g\nu_{21}$
984	$a_g\nu_{20}$
998	$a_g\nu_{20}$
1028	$a_g\nu_{19}$
1157	$a_g\nu_{17}$
1195	$a_g\nu_{15}$
1291	$a_g\nu_{14}$
1327	$a_g\nu_{13}$
1341	$a_g\nu_{12}$
1446	$a_g\nu_{11}$
1492	$a_g\nu_{10}$
1577	$a_g\nu_9$
1595	$a_g\nu_8$
1641	$a_g\nu_7$
1684	---

Figure S37. Fitted Raman spectrum of ***trans*-stilbene** post-radiation.[7]

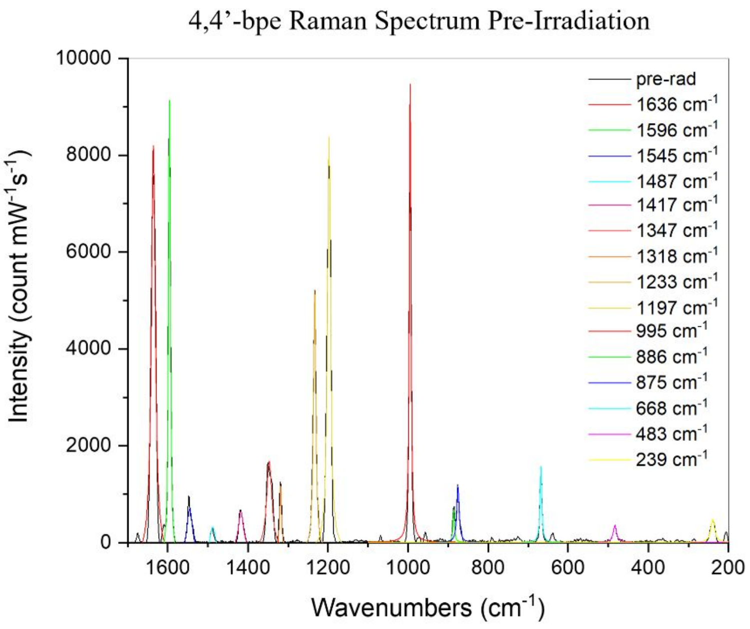

Raman Shift (cm-1)	Assignment (Symmetry)
239	$a_g\nu_{17}$
483	$b_g\nu_7$
668	$a_g\nu_{15}$
875	$a_g\nu_{14}$
886	$b_g\nu_4$
995	$a_g\nu_{13}$
1197	$a_g\nu_9$
1233	$a_g\nu_8$
1318	$a_g\nu_7$
1347	$a_g\nu_6$
1417	$a_g\nu_5$
1487	$a_g\nu_4$
1545	$a_g\nu_3$
1596	$a_g\nu_2$
1636	$a_g\nu_1$

Figure S38. Fitted Raman spectrum of **4,4'-bpe** pre-radiation.[8]

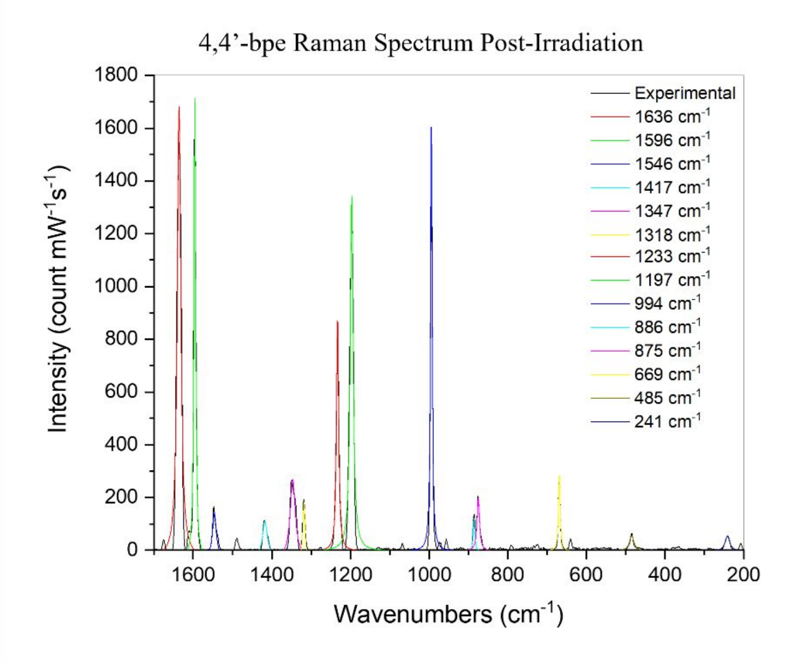

Raman Shift (cm-1)	Assignment (Symmetry)
241	$a_g\nu_{17}$
485	$b_g\nu_7$
669	$a_g\nu_{15}$
875	$a_g\nu_{14}$
886	$b_g\nu_4$
994	$a_g\nu_{13}$
1197	$a_g\nu_9$
1233	$a_g\nu_8$
1318	$a_g\nu_7$
1347	$a_g\nu_6$
1417	$a_g\nu_5$
1546	$a_g\nu_3$
1596	$a_g\nu_2$
1636	$a_g\nu_1$

Figure S39. Fitted Raman spectrum of **4,4’-bpe** post-radiation.[8]

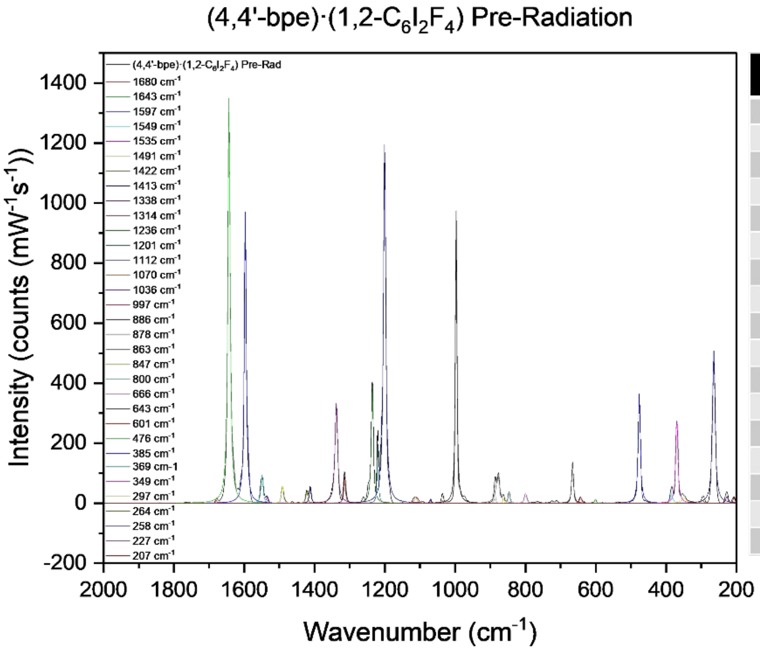

Raman Shift (cm-1)	Assignment (Symmetry)	Raman Shift (cm-1)	Assignment (Symmetry)
207	$b_1\nu_{17}$*	997	$a_g\nu_{13}$†
227	$a_1\nu_{10}$*	1036	$b_2\nu_{22}$*
258	$a_2\nu_{13}$*	1070	$a\nu_{29}$†
264	$a_2\nu_{13}$*	1112	$a_1\nu_5$*
297	$a_1\nu_9$*	1201	$a_g\nu_9$†
349	$b_2\nu_{26}$*	1236	$a_g\nu_8$†
369	$b_1\nu_{16}$*	1314	$a_g\nu_7$†
385	$b_1\nu_{16}$*	1338	$a_g\nu_6$†
476	$a_1\nu_7$*	1413	$a_g\nu_5$†
601	$a_2\nu_{12}$*	1422	$a_g\nu_5$†
643	$b_3\nu_{12}$†	1491	$a_1\nu_2$*
666	$a_g\nu_{15}$†	1535	$a_g\nu_3$†
800	$b_2\nu_{23}$*	1549	$a_g\nu_3$†
847	---	1597	$a_g\nu_3$†
863	---	1643	$a_g\nu_1$†
878	$a_g\nu_{14}$†	1680	---
886	$b_g\nu_4$†		

Figure S40. Fitted Raman spectrum of (**4,4’-bpe**)□(**1,2-$C_6I_2F_4$**) pre-radiation.[1,8]

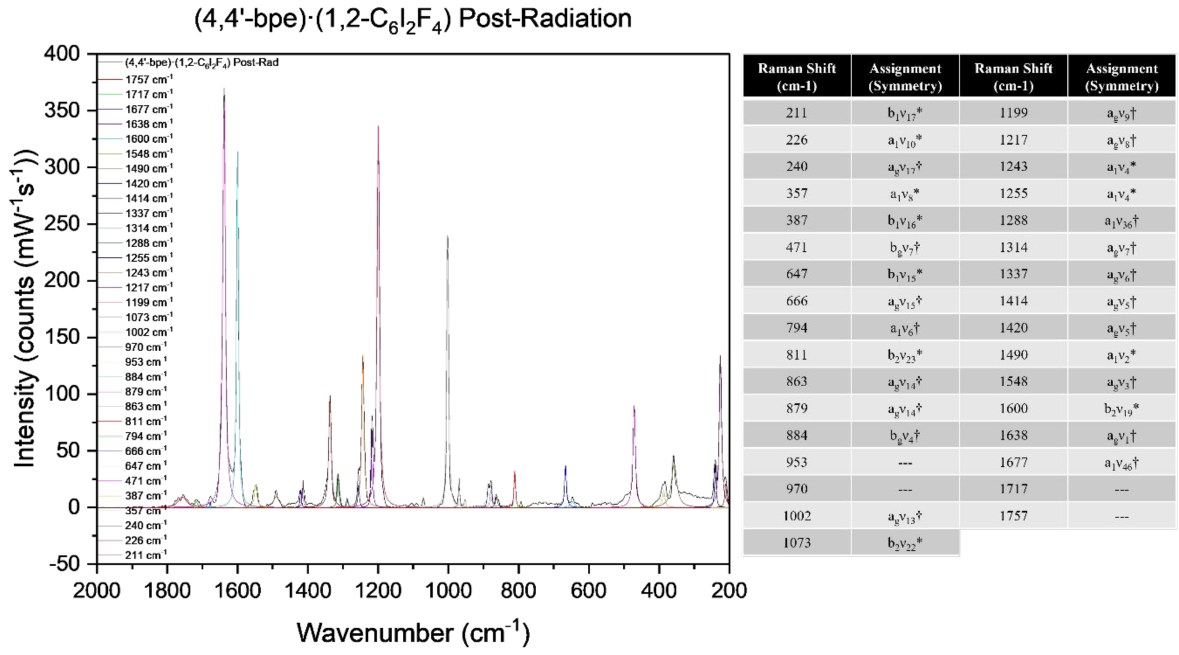

Raman Shift (cm-1)	Assignment (Symmetry)	Raman Shift (cm-1)	Assignment (Symmetry)
211	b_1v_{17}*	1199	a_gv_9†
226	a_1v_{10}*	1217	a_gv_8†
240	a_gv_{17}†	1243	a_1v_4*
357	a_1v_8*	1255	a_1v_4*
387	b_1v_{16}*	1288	a_1v_{36}†
471	b_gv_7†	1314	a_gv_7†
647	b_1v_{15}*	1337	a_gv_6†
666	a_gv_{15}†	1414	a_gv_5†
794	a_1v_6†	1420	a_gv_5†
811	b_2v_{23}*	1490	a_1v_2*
863	a_gv_{14}†	1548	a_gv_3†
879	a_gv_{14}†	1600	b_2v_{19}*
884	b_gv_4†	1638	a_gv_1†
953	---	1677	a_1v_{46}†
970	---	1717	---
1002	a_gv_{13}†	1757	---
1073	b_2v_{22}*		

Figure S41. Fitted Raman spectrum of (**4,4'-bpe**)□(**1,2-$C_6I_2F_4$**) post-radiation.[1,8]

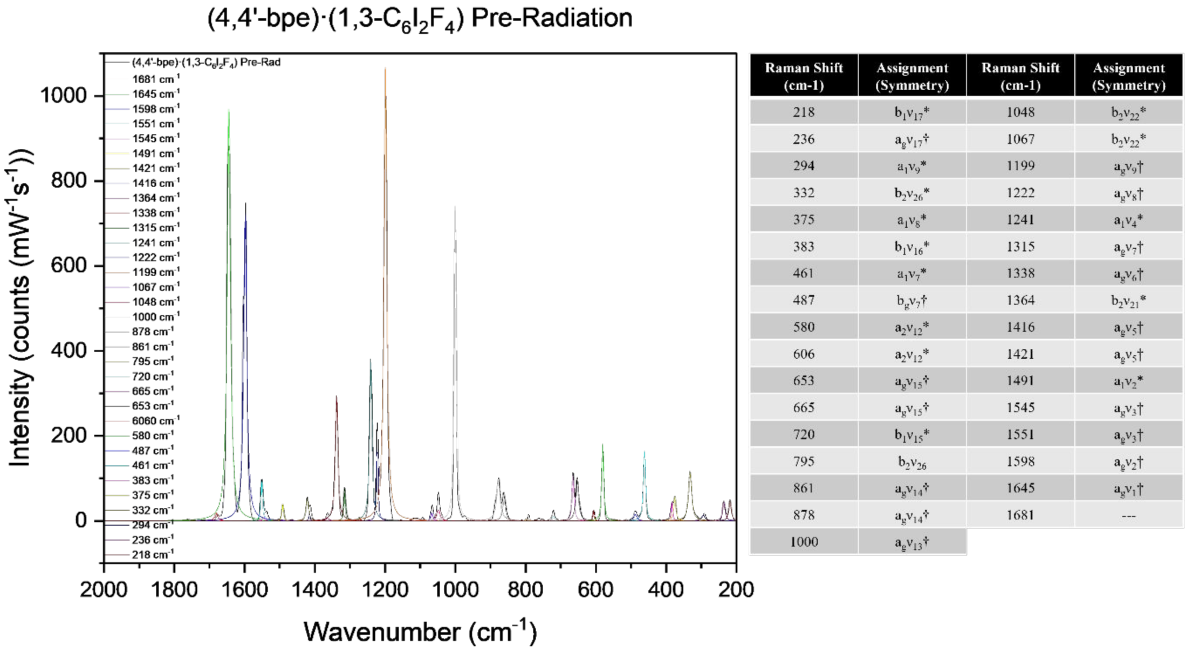

Raman Shift (cm-1)	Assignment (Symmetry)	Raman Shift (cm-1)	Assignment (Symmetry)
218	b_1v_{17}*	1048	b_2v_{22}*
236	a_gv_{17}†	1067	b_2v_{22}*
294	a_1v_9*	1199	a_gv_9†
332	b_2v_{26}*	1222	a_gv_8†
375	a_1v_8*	1241	a_1v_4*
383	b_1v_{16}*	1315	a_gv_7†
461	a_1v_7*	1338	a_gv_6†
487	b_gv_7†	1364	b_2v_{21}*
580	a_2v_{12}*	1416	a_gv_5†
606	a_2v_{12}*	1421	a_gv_5†
653	a_gv_{15}†	1491	a_1v_2*
665	a_gv_{15}†	1545	a_gv_3†
720	b_1v_{15}*	1551	a_gv_3†
795	b_2v_{26}	1598	a_gv_2†
861	a_gv_{14}†	1645	a_gv_1†
878	a_gv_{14}†	1681	---
1000	a_gv_{13}†		

Figure S42. Fitted Raman spectrum of (**4,4'-bpe**)□(**1,3-$C_6I_2F_4$**) pre-radiation.[3,8]

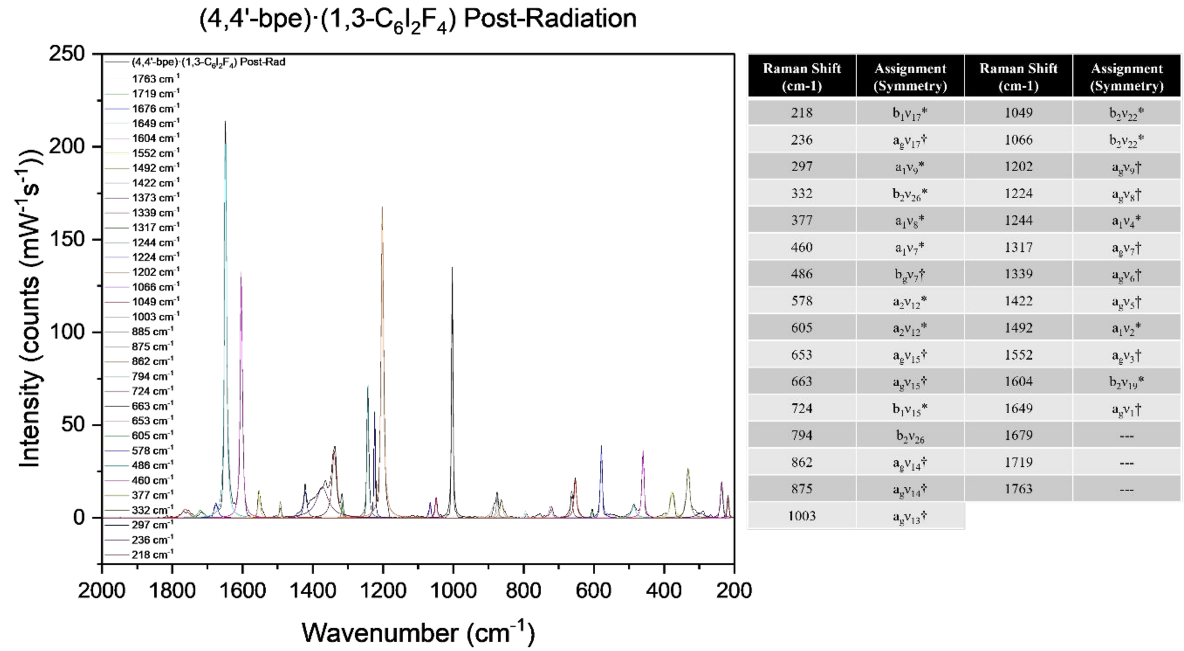

Raman Shift (cm-1)	Assignment (Symmetry)	Raman Shift (cm-1)	Assignment (Symmetry)
218	$b_1\nu_{17}$*	1049	$b_2\nu_{22}$*
236	$a_g\nu_{17}$†	1066	$b_2\nu_{22}$*
297	$a_1\nu_9$*	1202	$a_g\nu_9$†
332	$b_2\nu_{26}$*	1224	$a_g\nu_8$†
377	$a_1\nu_8$*	1244	$a_1\nu_4$*
460	$a_1\nu_7$*	1317	$a_g\nu_7$†
486	$b_g\nu_7$†	1339	$a_g\nu_6$†
578	$a_2\nu_{12}$*	1422	$a_g\nu_5$†
605	$a_2\nu_{12}$*	1492	$a_1\nu_2$*
653	$a_g\nu_{15}$†	1552	$a_g\nu_3$†
663	$a_g\nu_{15}$†	1604	$b_2\nu_{19}$*
724	$b_1\nu_{15}$*	1649	$a_g\nu_1$†
794	$b_2\nu_{26}$	1679	---
862	$a_g\nu_{14}$†	1719	---
875	$a_g\nu_{14}$†	1763	---
1003	$a_g\nu_{13}$†		

Figure S43. Fitted Raman spectrum of (**4,4'-bpe**)□(**1,3-$C_6I_2F_4$**) post-radiation. [3,8]

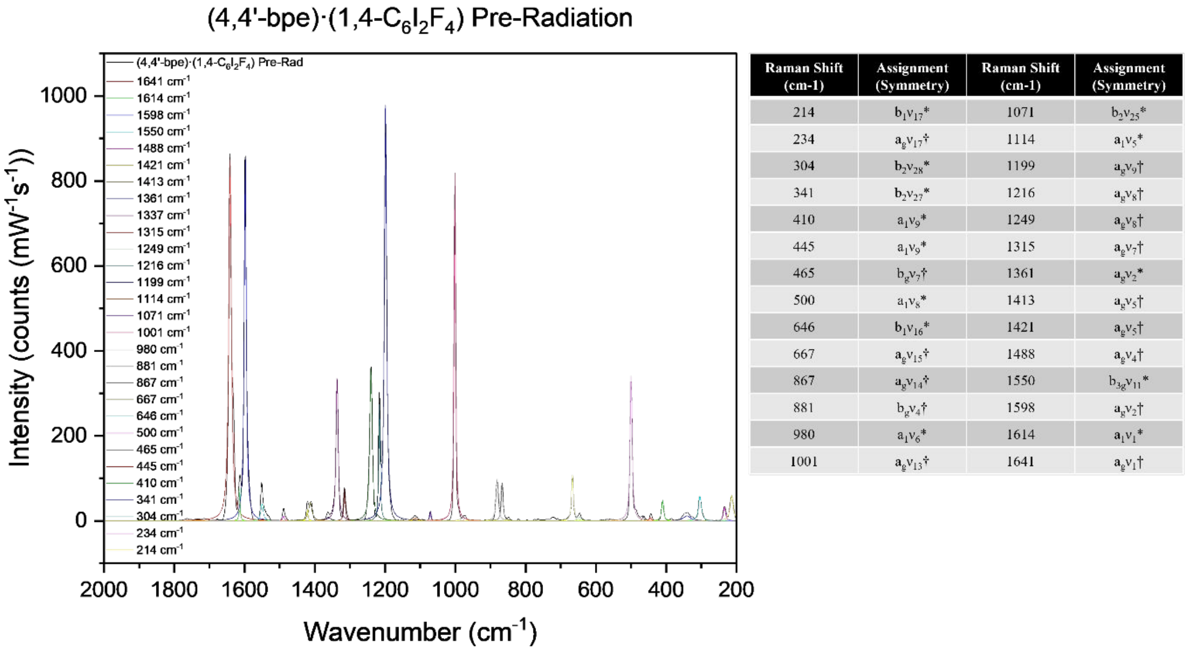

Raman Shift (cm-1)	Assignment (Symmetry)	Raman Shift (cm-1)	Assignment (Symmetry)
214	$b_1\nu_{17}$*	1071	$b_2\nu_{25}$*
234	$a_g\nu_{17}$†	1114	$a_1\nu_5$*
304	$b_2\nu_{28}$*	1199	$a_g\nu_9$†
341	$b_2\nu_{27}$*	1216	$a_g\nu_8$†
410	$a_1\nu_9$*	1249	$a_g\nu_8$†
445	$a_1\nu_9$*	1315	$a_g\nu_7$†
465	$b_g\nu_7$†	1361	$a_g\nu_2$*
500	$a_1\nu_8$*	1413	$a_g\nu_5$†
646	$b_1\nu_{16}$*	1421	$a_g\nu_5$†
667	$a_g\nu_{15}$†	1488	$a_g\nu_4$†
867	$a_g\nu_{14}$†	1550	$b_{3g}\nu_{11}$*
881	$b_g\nu_4$†	1598	$a_g\nu_2$†
980	$a_1\nu_6$*	1614	$a_1\nu_1$*
1001	$a_g\nu_{13}$†	1641	$a_g\nu_1$†

Figure S44. Fitted Raman spectrum of (**4,4'-bpe**)□(**1,4-$C_6I_2F_4$**) pre-radiation. [2,8]

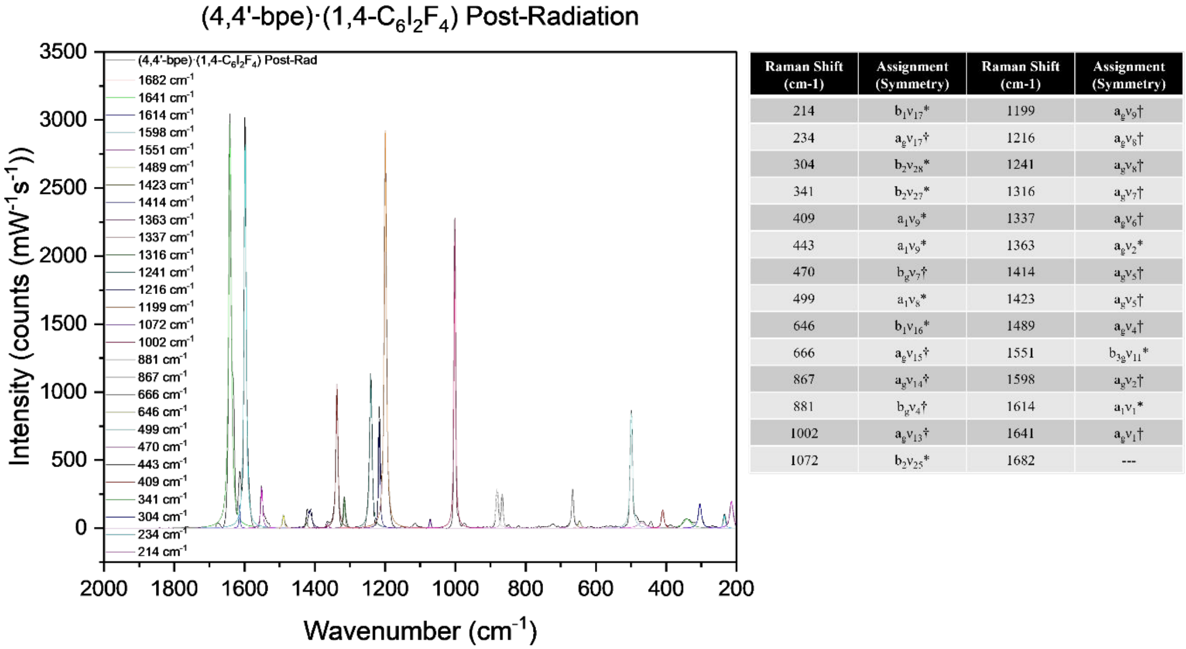

Raman Shift (cm-1)	Assignment (Symmetry)	Raman Shift (cm-1)	Assignment (Symmetry)
214	$b_1\nu_{17}$*	1199	$a_g\nu_9$†
234	$a_g\nu_{17}$†	1216	$a_g\nu_8$†
304	$b_2\nu_{28}$*	1241	$a_g\nu_8$†
341	$b_2\nu_{27}$*	1316	$a_g\nu_7$†
409	$a_1\nu_9$*	1337	$a_g\nu_6$†
443	$a_1\nu_9$*	1363	$a_g\nu_2$*
470	$b_g\nu_7$†	1414	$a_g\nu_5$†
499	$a_1\nu_8$*	1423	$a_g\nu_5$†
646	$b_1\nu_{16}$*	1489	$a_g\nu_4$†
666	$a_g\nu_{15}$†	1551	$b_{3g}\nu_{11}$*
867	$a_g\nu_{14}$†	1598	$a_g\nu_2$†
881	$b_g\nu_4$†	1614	$a_1\nu_1$*
1002	$a_g\nu_{13}$†	1641	$a_g\nu_1$†
1072	$b_2\nu_{25}$*	1682	---

Figure S45. Fitted Raman spectrum of (**4,4'-bpe**)□(**1,4-$C_6I_2F_4$**) post-radiation. [2,8]

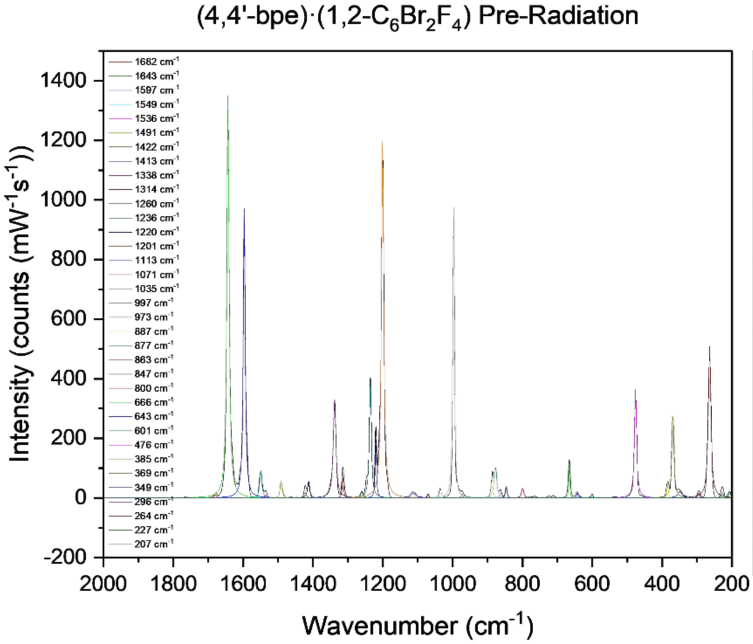

Raman Shift (cm-1)	Assignment (Symmetry)	Raman Shift (cm-1)	Assignment (Symmetry)
207	$b_1\nu_{19}$*	1035	$a_1\nu_5$*
227	$a_g\nu_{17}$†	1071	---
264	$a_1\nu_9$*	1113	$b_2\nu_{24}$*
296	$b_2\nu_{29}$*	1201	---
349	$b_2\nu_{28}$*	1220	$a_g\nu_8$†
369	$b_1\nu_{18}$*	1236	$a_g\nu_8$†
385	$a_1\nu_8$*	1260	$b_2\nu_{23}$*
476	$b_g\nu_7$†	1314	$a_g\nu_7$†
601	$b_1\nu_{17}$*	1338	$a_g\nu_6$†
643	$b_2\nu_{26}$*	1413	$a_g\nu_5$†
666	$a_g\nu_{15}$†	1422	$a_g\nu_5$†
800	$a_1\nu_6$*	1491	$a_g\nu_4$*
847	$a_1\nu_6$*	1536	$a_1\nu_2$*
863	$a_1\nu_6$*	1549	$a_g\nu_3$†
877	$a_g\nu_{14}$†	1597	$a_g\nu_2$†
887	$b_g\nu_4$†	1643	$a_g\nu_1$†
973	$b_2\nu_{25}$*	1682	---
997	$a_g\nu_{13}$†		

Figure S46. Fitted Raman spectrum of (**4,4'-bpe**)□(**1,2-$C_6Br_2F_4$**) pre-radiation. [3,8]

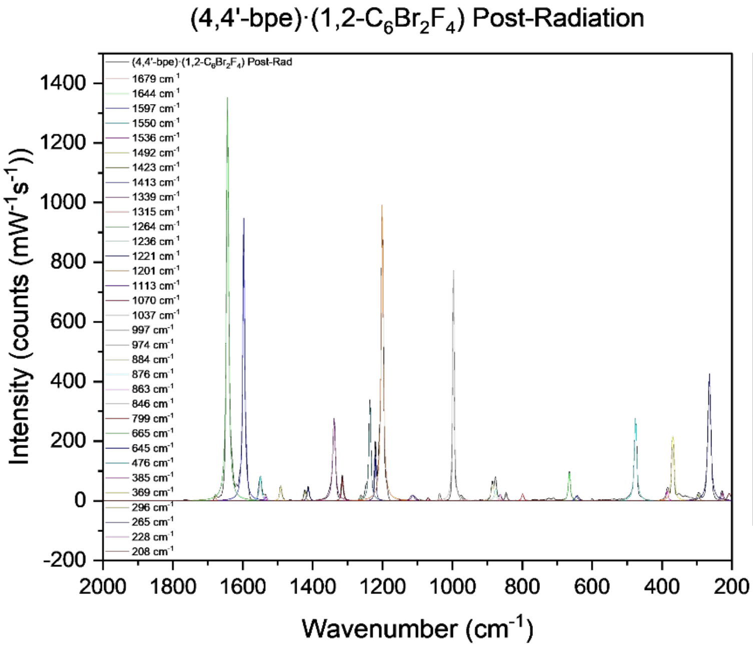

Raman Shift (cm-1)	Assignment (Symmetry)	Raman Shift (cm-1)	Assignment (Symmetry)
208	b_1v_{19}*	1037	a_1v_5*
228	a_gv_{17}†	1070	---
264	a_1v_9*	1113	b_2v_{24}*
296	b_2v_{29}*	1201	---
369	b_1v_{18}*	1221	a_gv_8†
385	a_1v_8*	1236	a_gv_8†
476	b_gv_7†	1264	b_2v_{23}*
645	b_2v_{26}*	1315	a_gv_7†
665	a_gv_{15}†	1339	a_gv_6†
799	a_1v_6*	1413	a_gv_5†
846	a_1v_6*	1423	a_gv_5†
863	a_1v_6*	1492	a_gv_4*
876	a_gv_{14}†	1536	a_1v_2*
884	b_gv_4†	1550	a_gv_3†
974	b_2v_{25}*	1597	a_gv_2†
997	a_gv_{13}†	1644	a_gv_1†
		1679	---

Figure S47. Fitted Raman spectrum of (**4,4'-bpe**)□(**1,2-$C_6Br_2F_4$**) post-radiation. [3,8]

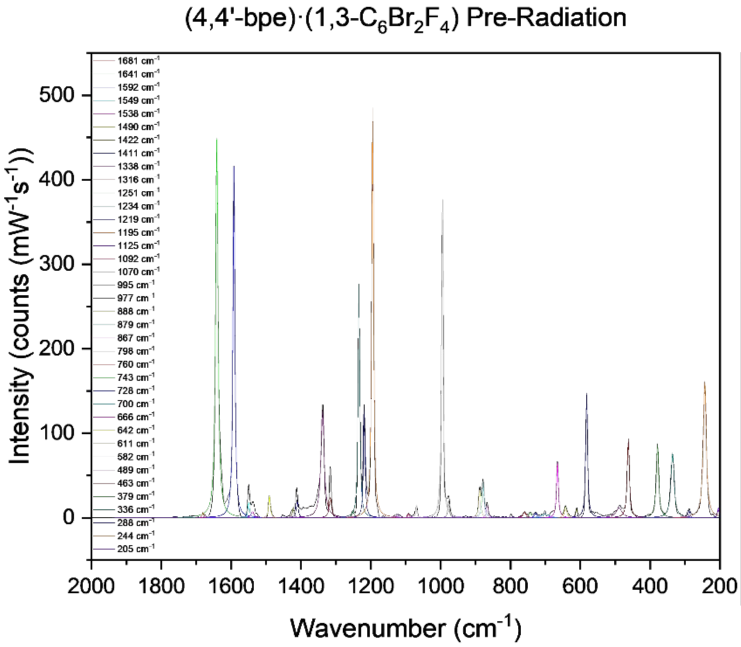

Raman Shift (cm-1)	Assignment (Symmetry)	Raman Shift (cm-1)	Assignment (Symmetry)
205	b_1v_{19}*	977	a_1v_6*
244	a_gv_{17}†	995	a_gv_{13}†
288	b_2v_{28}*	1070	a_1v_5*
336	b_2v_{27}*	1092	b_2v_{24}*
379	b_1v_{17}*	1125	b_2v_{24}*
463	a_1v_8*	1195	a_gv_9†
489	b_gv_7†	1219	---
582	a_1v_7*	1234	a_gv_8†
611	a_2v_{12}*	1251	b_2v_{24}*
642	b_1v_{16}*	1316	a_gv_7†
666	a_gv_{15}†	1338	a_gv_6†
700	b_1v_{15}*	1411	a_gv_5†
728	b_2v_{26}*	1422	a_gv_5†
743	b_2v_{26}*	1490	a_gv_4†
760	b_2v_{26}*	1538	a_1v_2*
798	a_1v_6*	1549	a_gv_3
867	a_gv_{14}†	1592	b_2v_{22}*
879	a_gv_{14}†	1641	a_gv_1†
888	b_gv_4†	1681	---

Figure S48. Fitted Raman spectrum of (**4,4'-bpe**)□(**1,3-$C_6Br_2F_4$**) pre-radiation. [3,8]

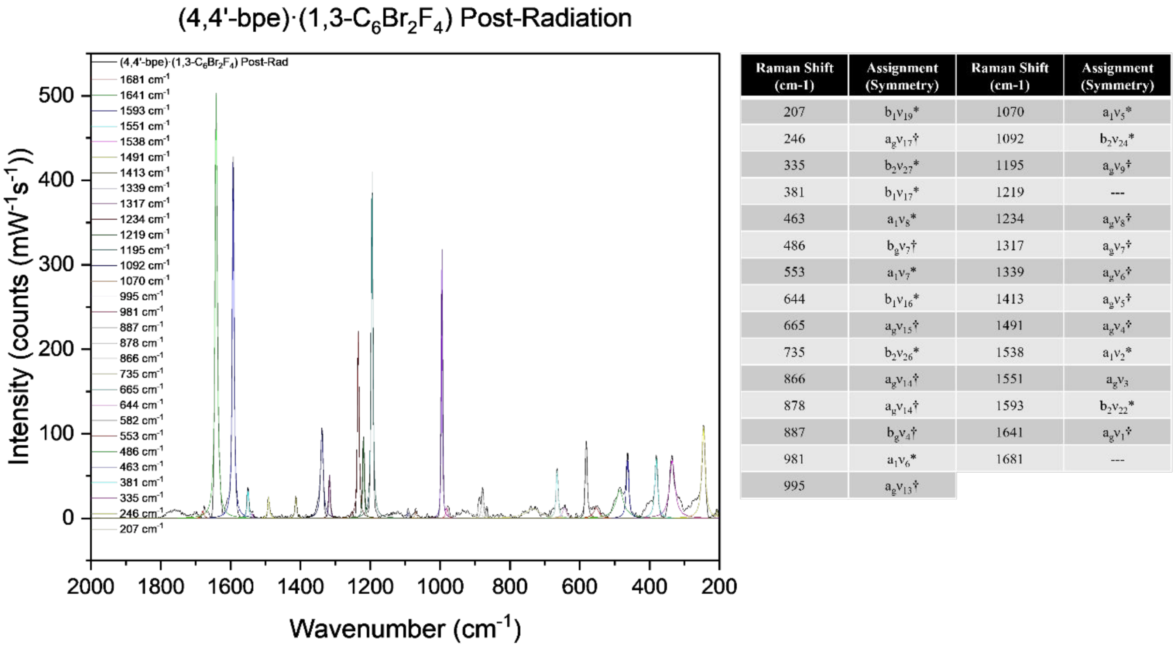

Raman Shift (cm-1)	Assignment (Symmetry)	Raman Shift (cm-1)	Assignment (Symmetry)
207	$b_1\nu_{19}$*	1070	$a_1\nu_5$*
246	$a_g\nu_{17}$†	1092	$b_2\nu_{24}$*
335	$b_2\nu_{27}$*	1195	$a_g\nu_9$†
381	$b_1\nu_{17}$*	1219	---
463	$a_1\nu_8$*	1234	$a_g\nu_8$†
486	$b_g\nu_7$†	1317	$a_g\nu_7$†
553	$a_1\nu_7$*	1339	$a_g\nu_6$†
644	$b_1\nu_{16}$*	1413	$a_g\nu_5$†
665	$a_g\nu_{15}$†	1491	$a_g\nu_4$†
735	$b_2\nu_{26}$*	1538	$a_1\nu_2$*
866	$a_g\nu_{14}$†	1551	$a_g\nu_3$
878	$a_g\nu_{14}$†	1593	$b_2\nu_{22}$*
887	$b_g\nu_4$†	1641	$a_g\nu_1$†
981	$a_1\nu_6$*	1681	---
995	$a_g\nu_{13}$†		

Figure S49. Fitted Raman spectrum of (**4,4'-bpe**)□(**1,3-$C_6Br_2F_4$**) post-radiation. [3,8]

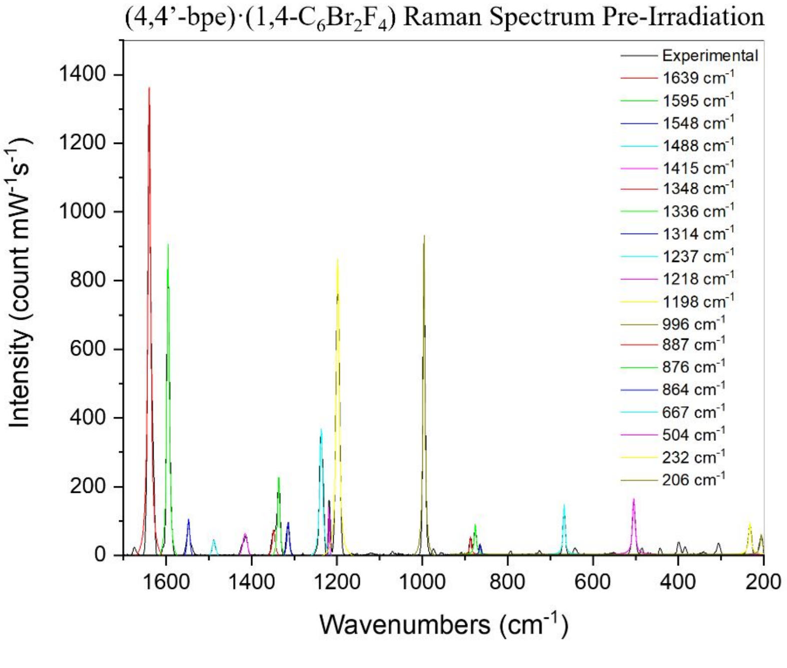

Raman Shift (cm-1)	Assignment (Symmetry)
206	$a_g\ \nu_6$*
232	$a_g\ \nu_6$*
504	$a_g\ \nu_4$*
667	$a_g\nu_{15}$†
864	$a_g\nu_{14}$†
876	$a_g\nu_{14}$†
887	$b_g\nu_4$†
996	$a_g\nu_{13}$†
1198	$a_g\nu_9$†
1218	$a_g\nu_8$†
1237	$a_g\nu_8$†
1314	$a_g\nu_7$†
1336	$a_g\nu_7$†
1348	$a_g\nu_6$†
1415	$a_g\nu_5$†
1488	$a_g\nu_4$†
1548	$a_g\nu_3$†
1595	$a_g\nu_2$†
1639	$a_g\nu_1$†

Figure S50. Fitted Raman spectrum of (**4,4'-bpe**)□(**1,4-$C_6Br_2F_4$**) pre-radiation. [3,8]

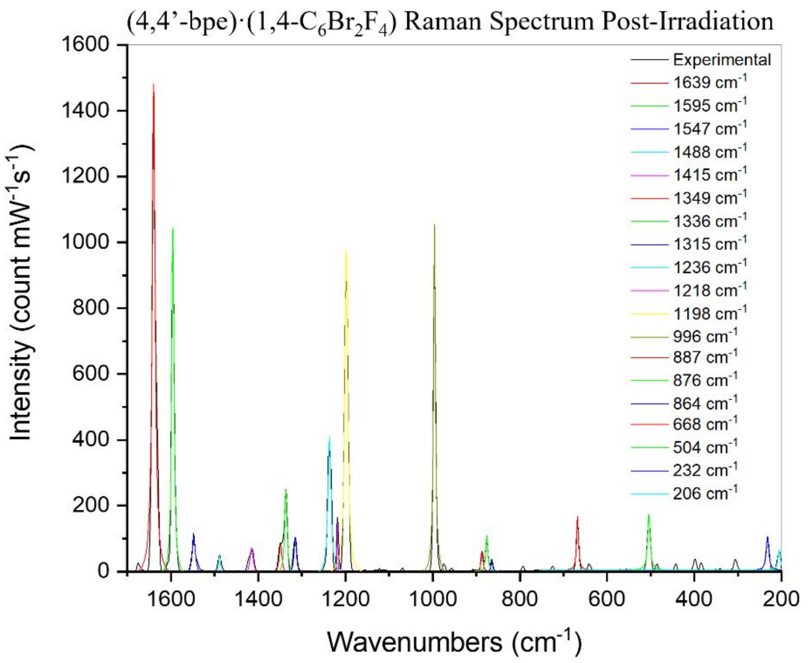

Raman Shift (cm-1)	Assignment (Symmetry)
206	$a_g \nu_6$*
232	$a_g \nu_6$*
504	$a_g \nu_4$*
668	$a_g \nu_{15}$†
864	$a_g \nu_{14}$†
876	$a_g \nu_{14}$†
887	$b_g \nu_4$†
996	$a_g \nu_{13}$†
1198	$a_g \nu_9$†
1218	$a_g \nu_8$†
1236	$a_g \nu_8$†
1315	$a_g \nu_7$†
1336	$a_g \nu_7$†
1349	$a_g \nu_6$†
1415	$a_g \nu_5$†
1488	$a_g \nu_4$†
1547	$a_g \nu_3$†
1595	$a_g \nu_2$†
1639	$a_g \nu_1$†

Figure S51. Fitted Raman spectrum of (**4,4'-bpe**)□(**1,4-$C_6Br_2F_4$**) post-radiation. [3,8]

Solid-State Fluorimetry

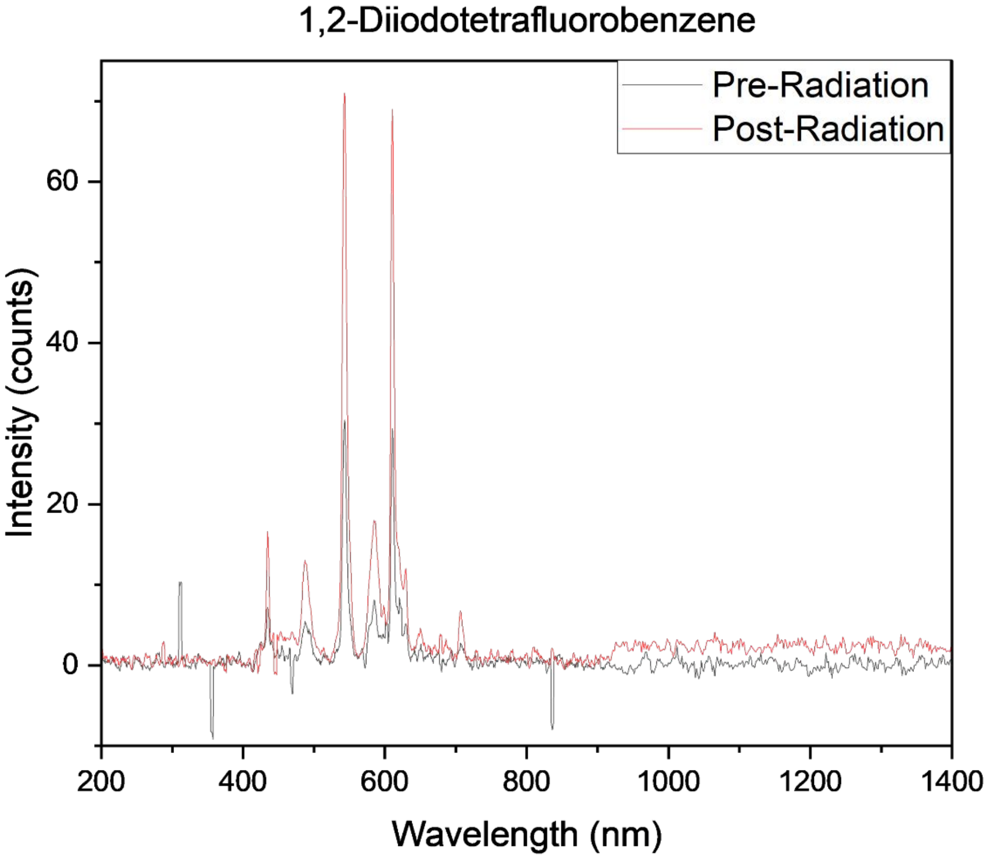

Figure S52. Solid-state fluorescence spectra of **1,2-$C_6I_2F_4$** pre- (black) and post-irradiation (red).

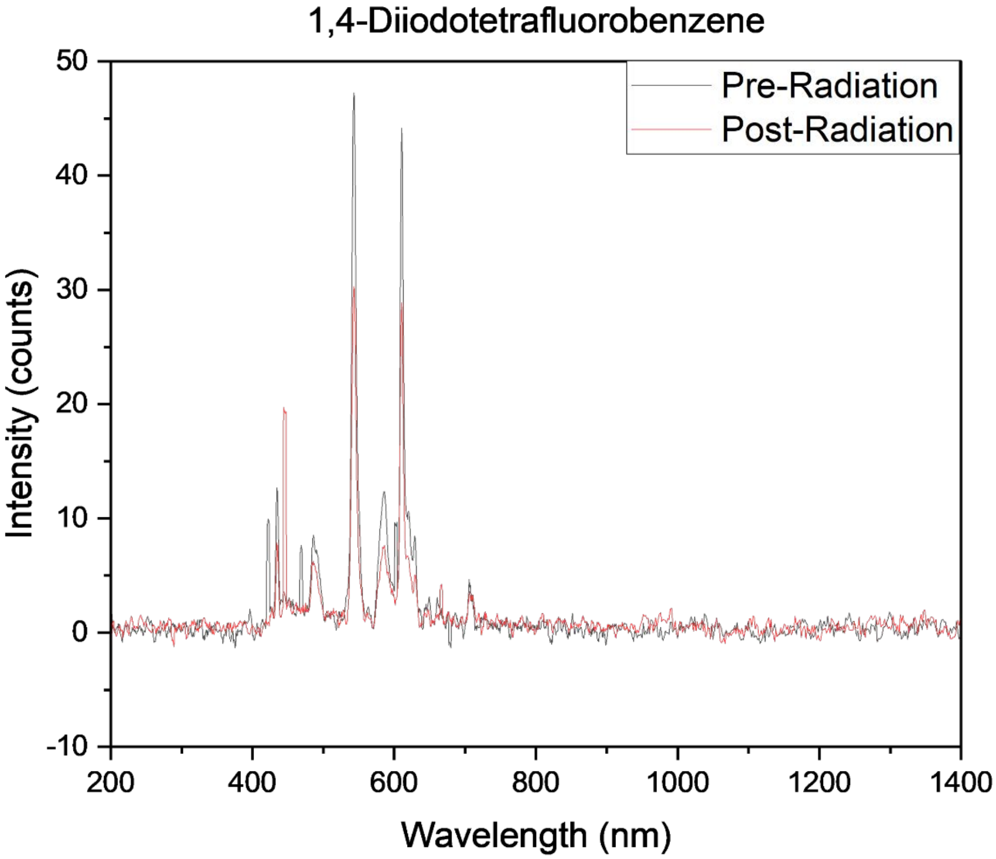

Figure S53. Solid-state fluorescence spectra of **1,4-$C_6I_2F_4$** pre- (black) and post-irradiation (red).

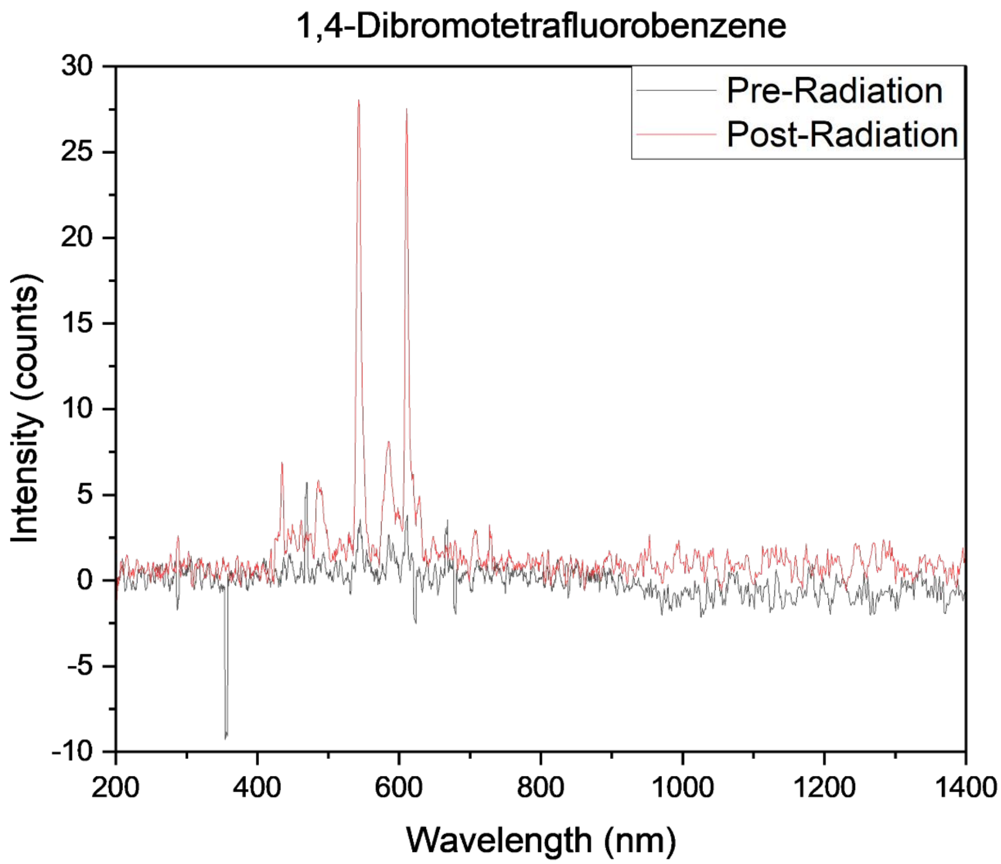

Figure S54. Solid-state fluorescence spectra of **1,4-$C_6Br_2F_4$** pre- (black) and post-irradiation (red).

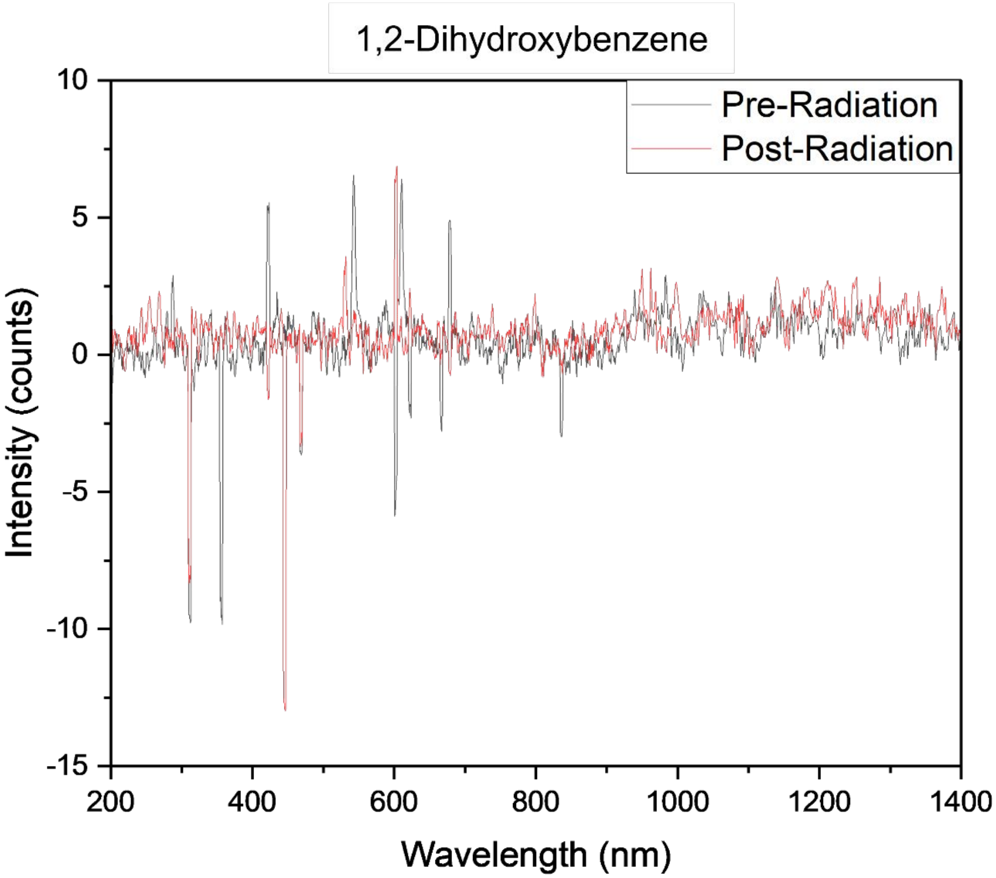

Figure S55. Solid-state fluorescence spectra of **1,2-$C_6H_6O_2$** pre- (black) and post-irradiation (red).

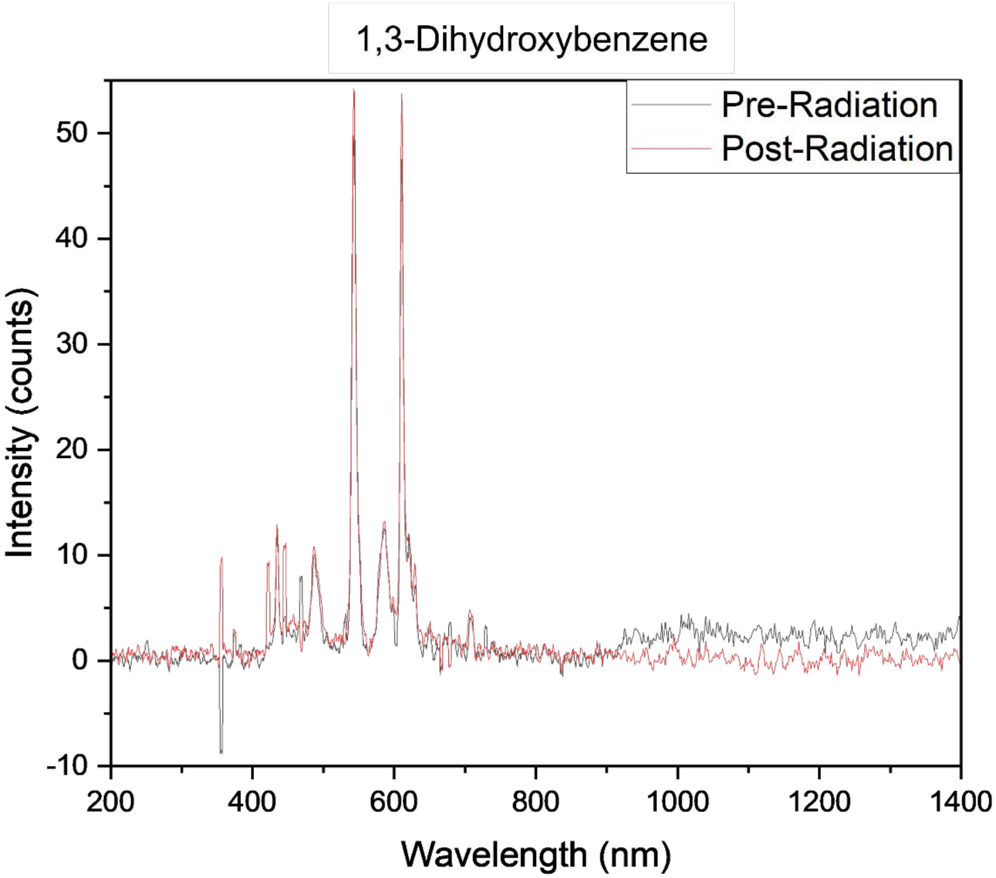

Figure S56. Solid-state fluorescence spectra of **1,3-$C_6H_6O_2$** pre- (black) and post-irradiation (red).

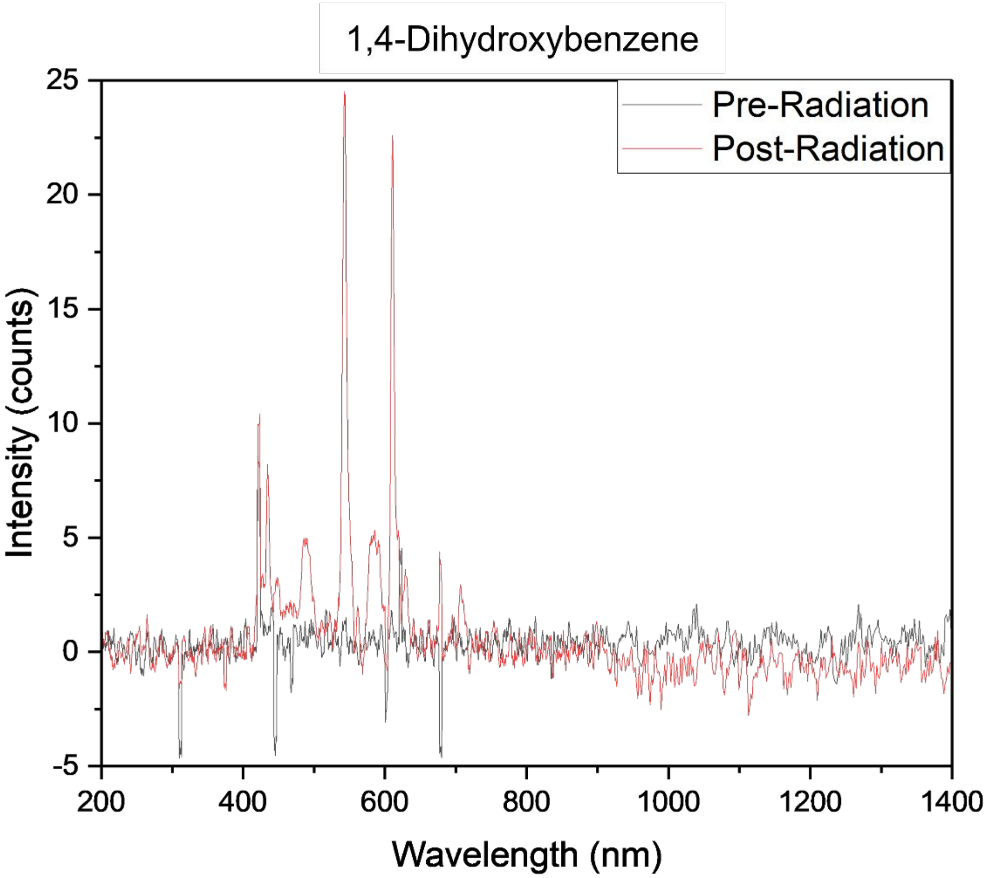

Figure S57. Solid-state fluorescence spectra of **1,4-$C_6H_6O_2$** pre- (black) and post-irradiation (red).

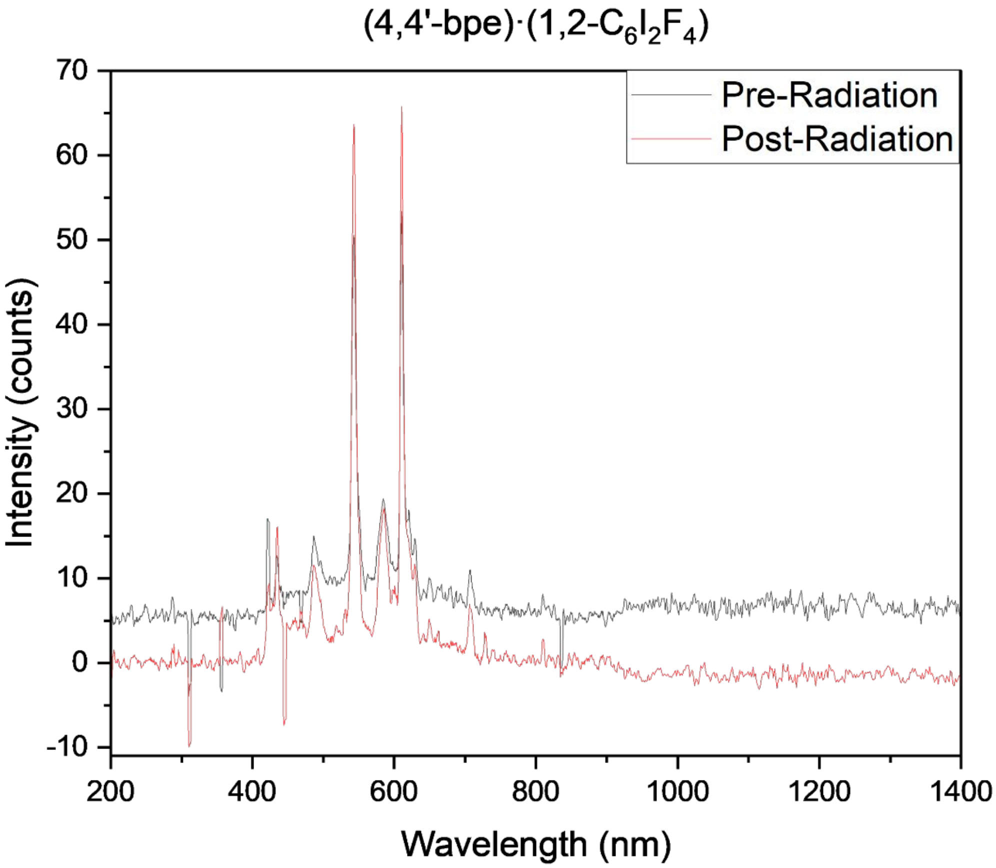

Figure S58. Solid-state fluorescence spectra of (**4,4'-bpe**)□(1**,2-$C_6I_2F_4$**) pre- (black) and post-irradiation (red).

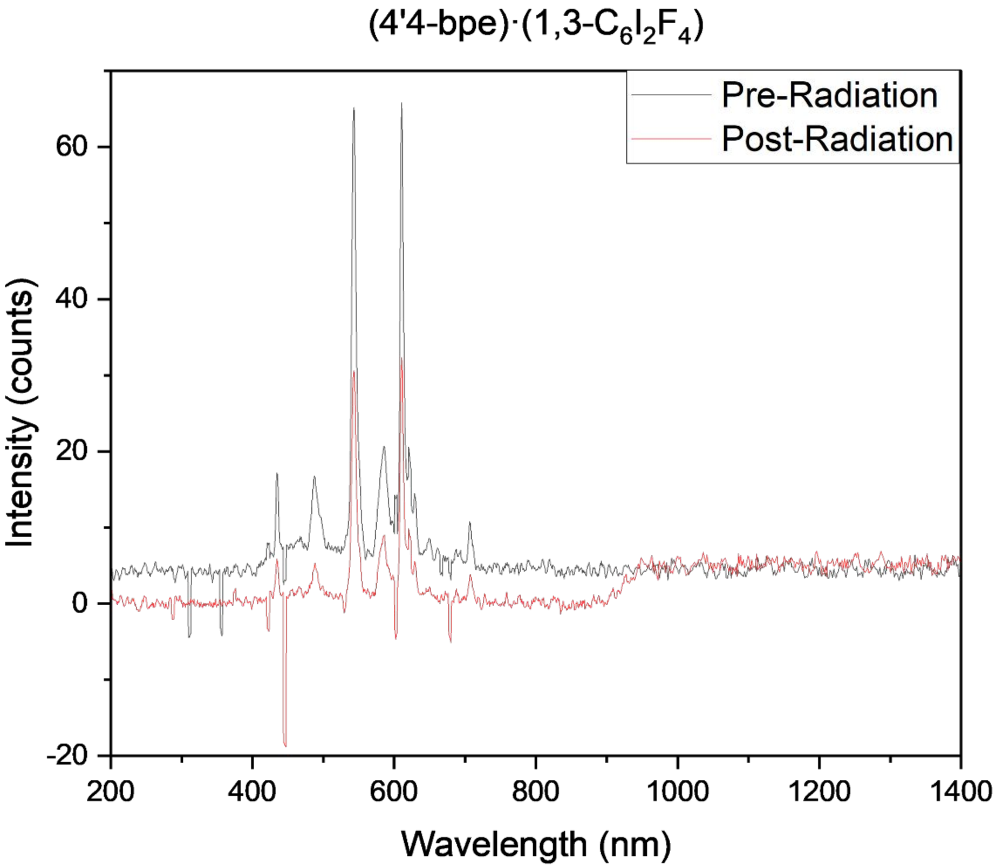

Figure S59. Solid-state fluorescence spectra of (**4,4'-bpe**)□(1**,3-**$\mathbf{C_6I_2F_4}$) pre- (black) and post-irradiation (red).

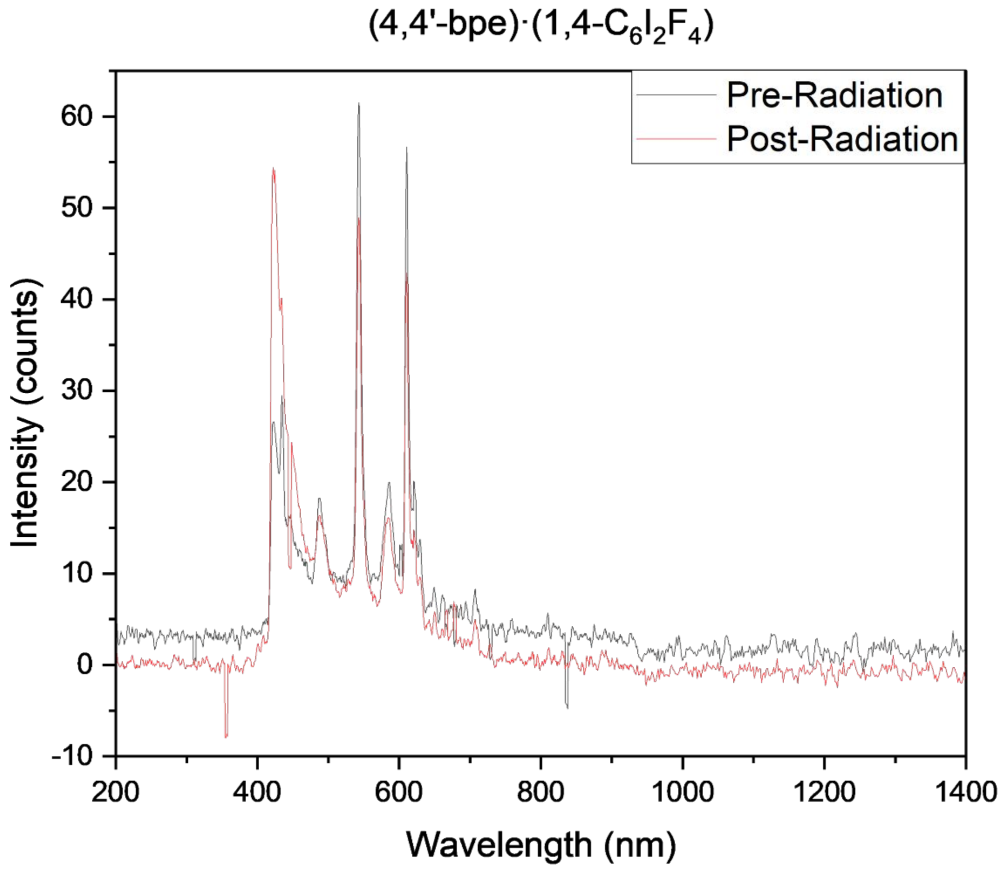

Figure S60. Solid-state fluorescence spectra of (**4,4'-bpe**)□(1**,4-**$C_6I_2F_4$) pre- (black) and post-irradiation (red).

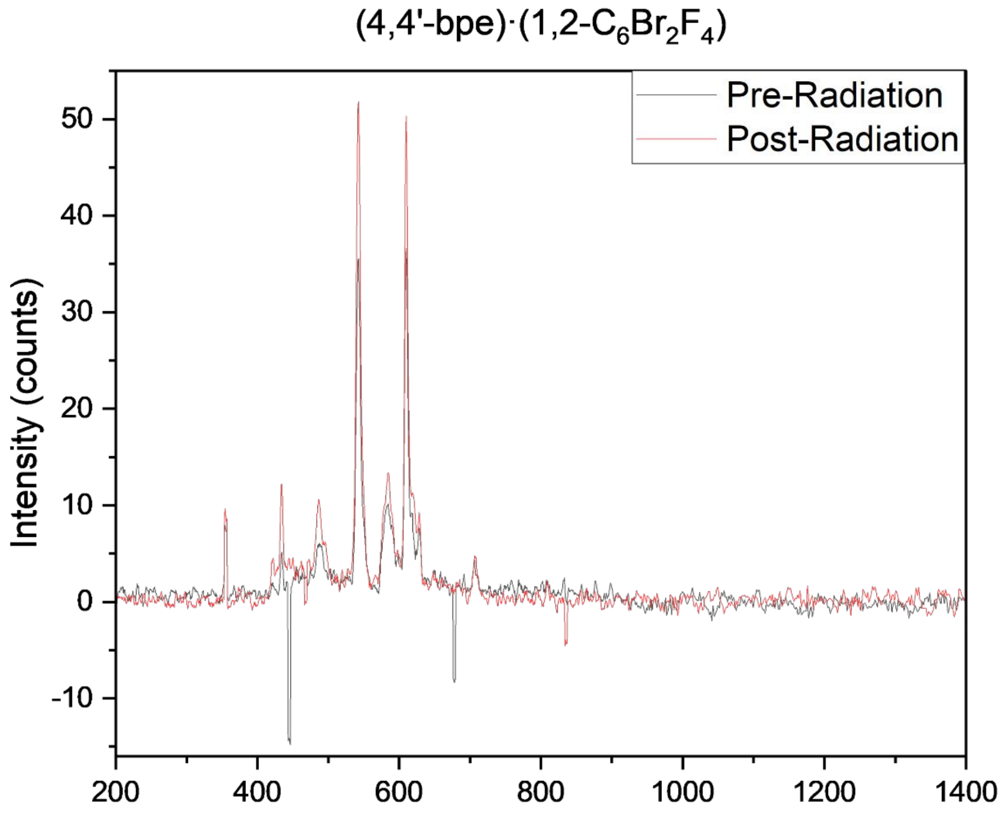

Figure S61. Solid-state fluorescence spectra of (**4,4’-bpe**)(1,**2-**$C_6Br_2F_4$) pre- (black) and post-irradiation (red).

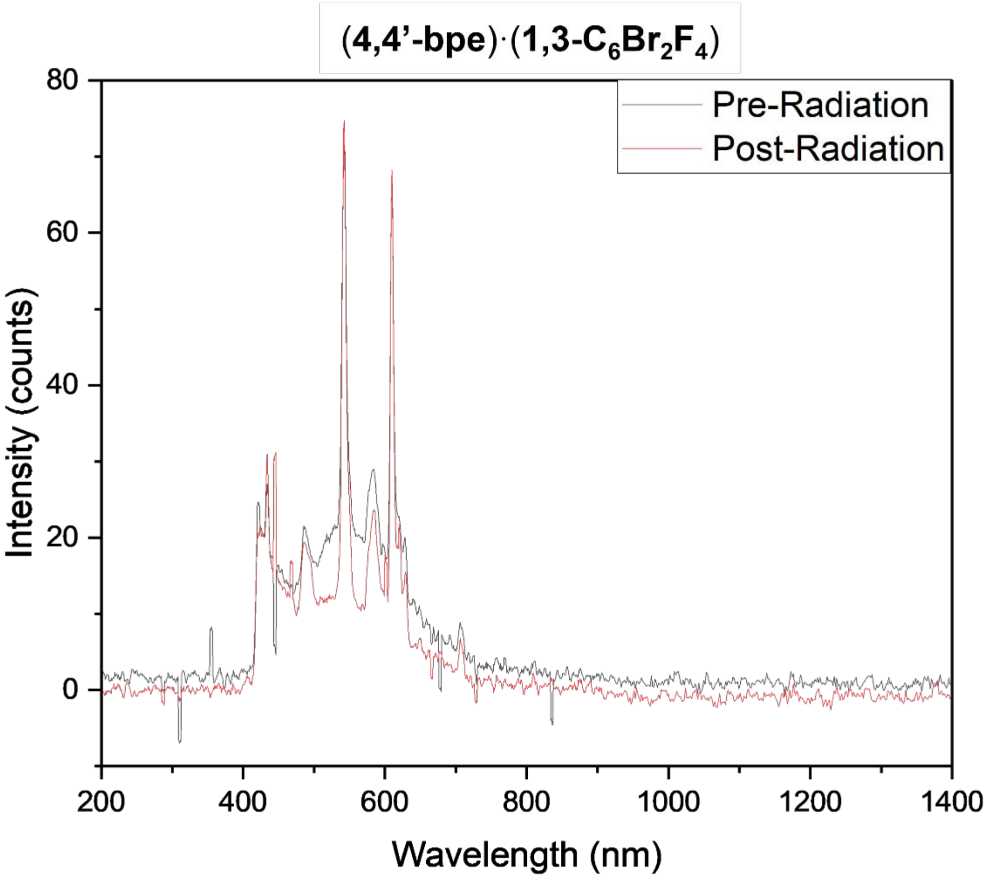

Figure S62. Solid-state fluorescence spectra of (**4,4'-bpe**)(1**,3-**$C_6Br_2F_4$) pre- (black) and post-irradiation (red).

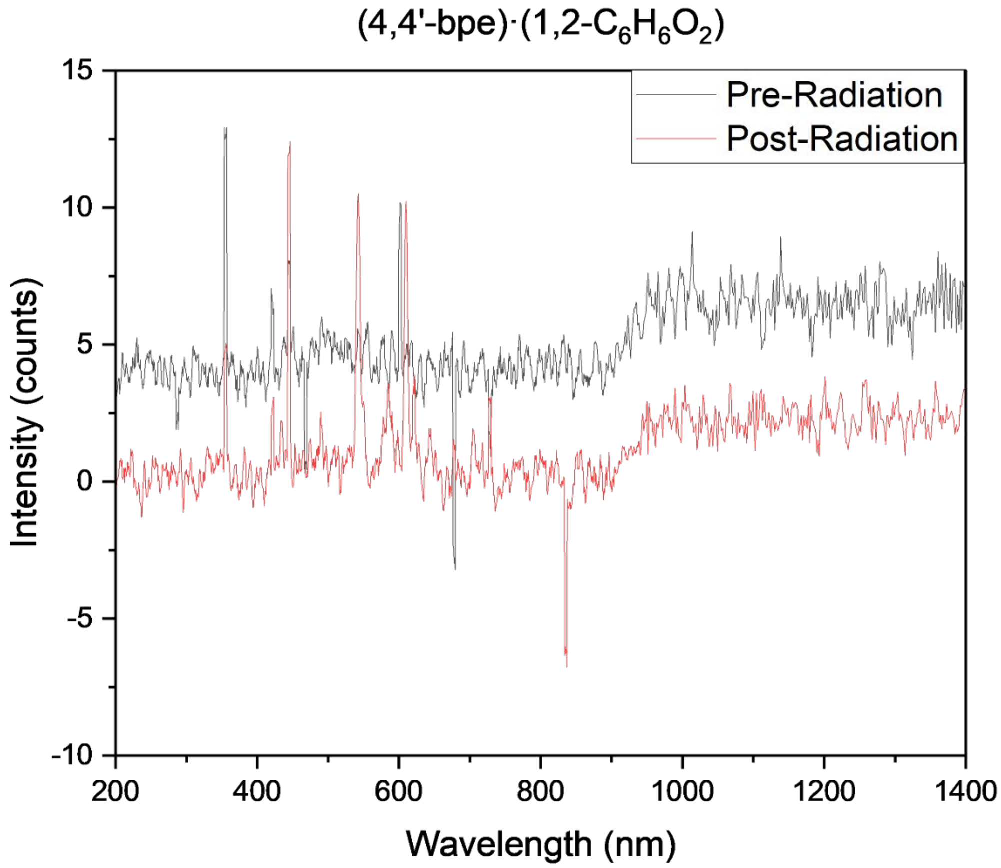

Figure S63. Solid-state fluorescence spectra of (**4,4'-bpe**)□(1**,2-$C_6H_6O_2$**) pre- (black) and post-irradiation (red).

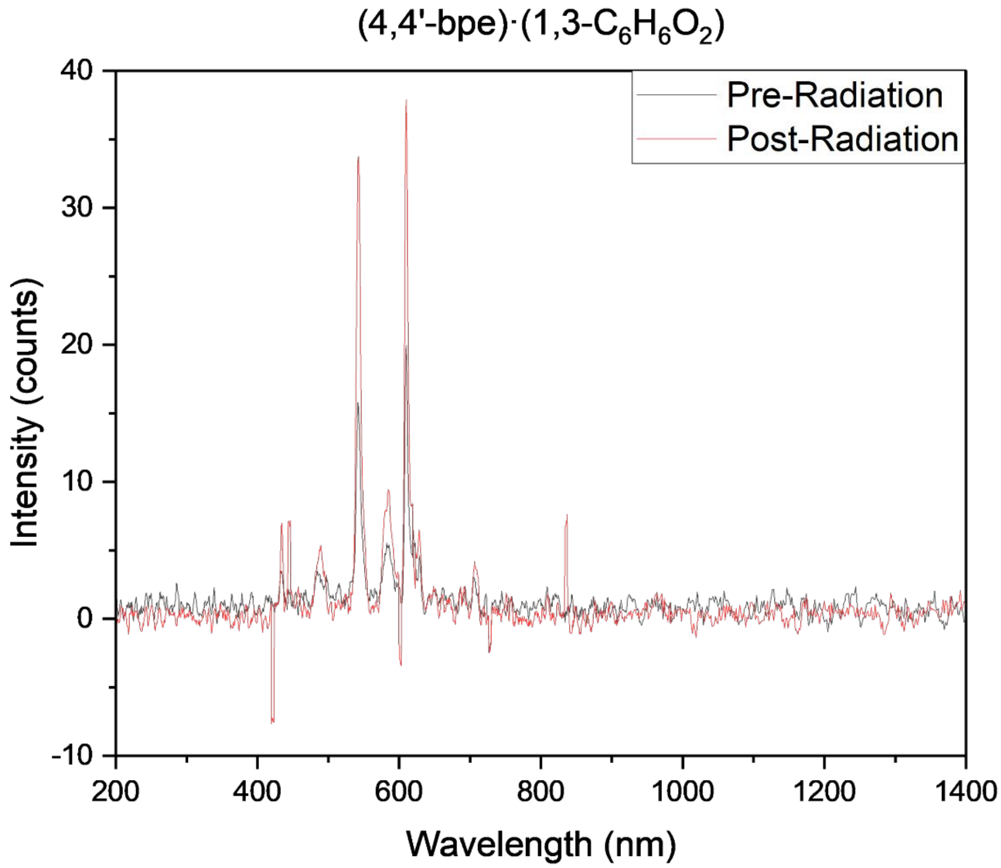

Figure S64. Solid-state fluorescence spectra of (**4,4’-bpe**)□(1**,3-**$C_6H_6O_2$) pre- (black) and post-irradiation (red).

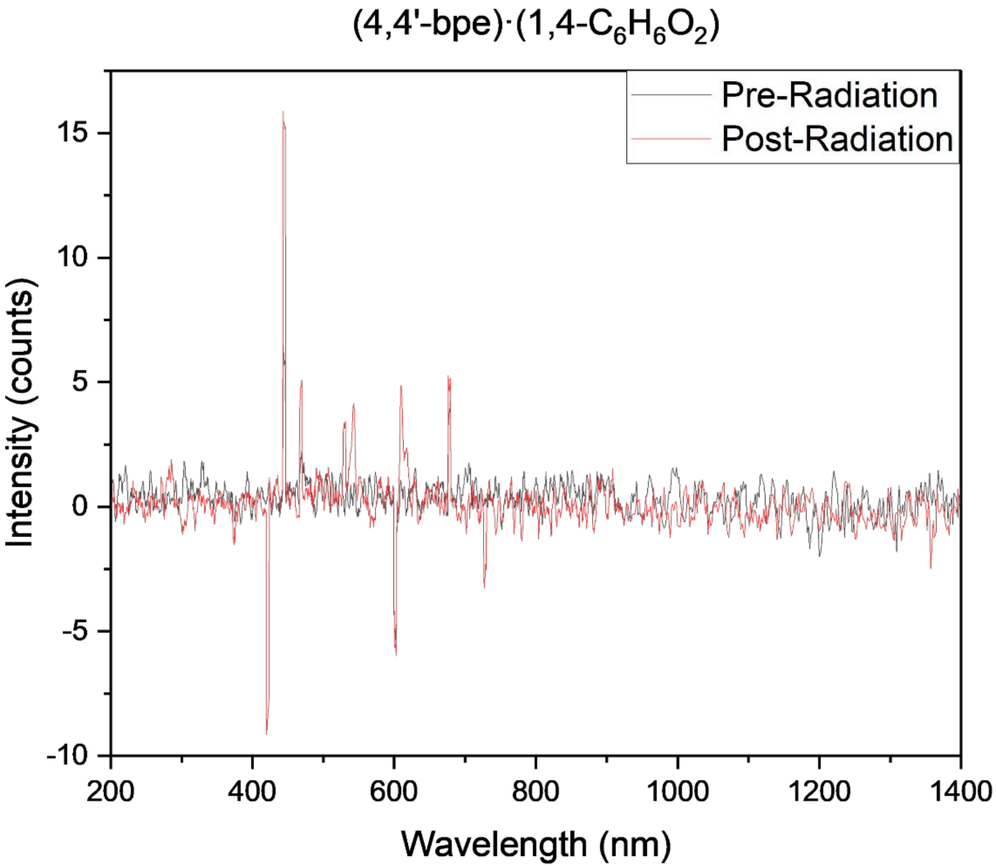

Figure S65. Solid-state fluorescence spectra of (**4,4'-bpe**)□(1,**4-$C_6H_6O_2$**) pre- (black) and post-irradiation (red).

Differential Scanning Calorimetry

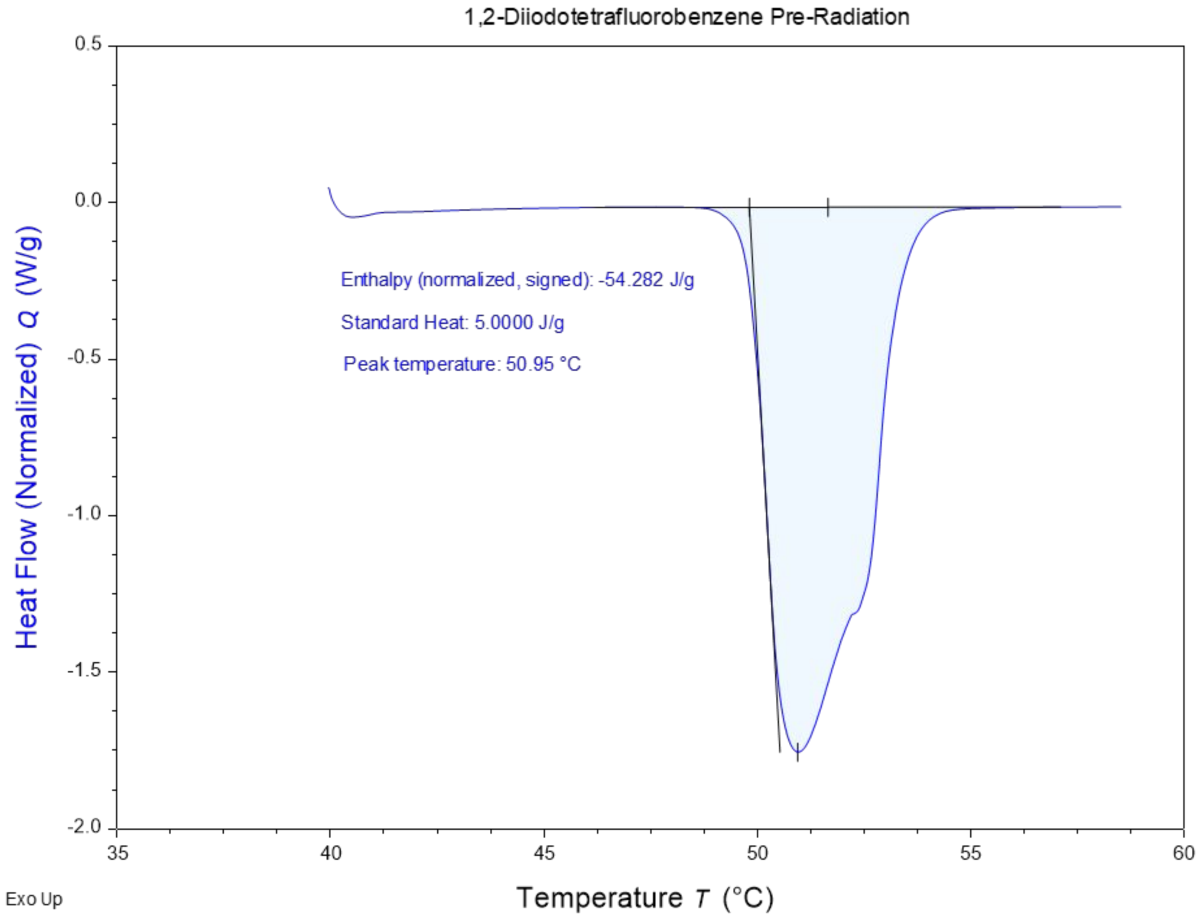

Figure S66. Integrated differential thermogram of **1,2-$C_6I_2F_4$** pre-radiation.

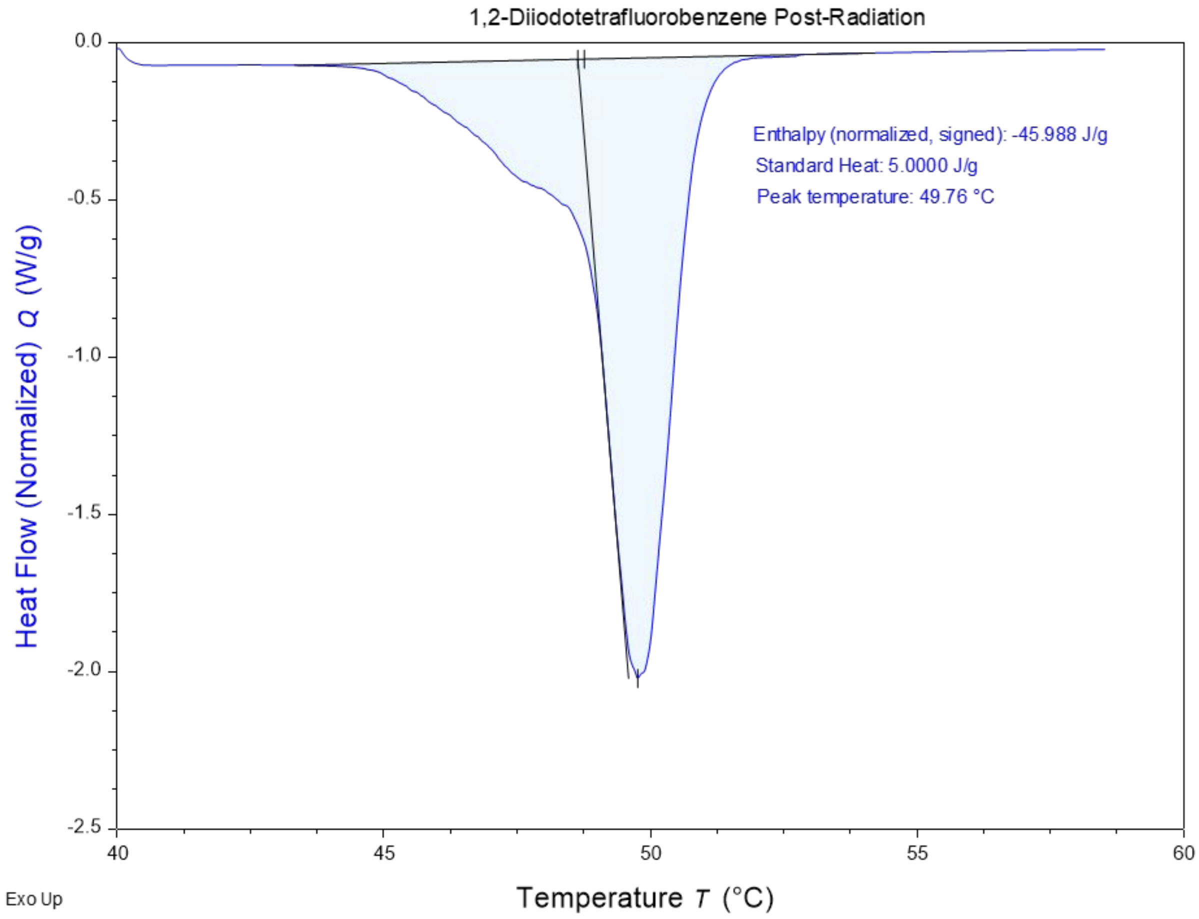

Figure S67. Integrated differential thermogram of **1,2-$C_6I_2F_4$** post-radiation.

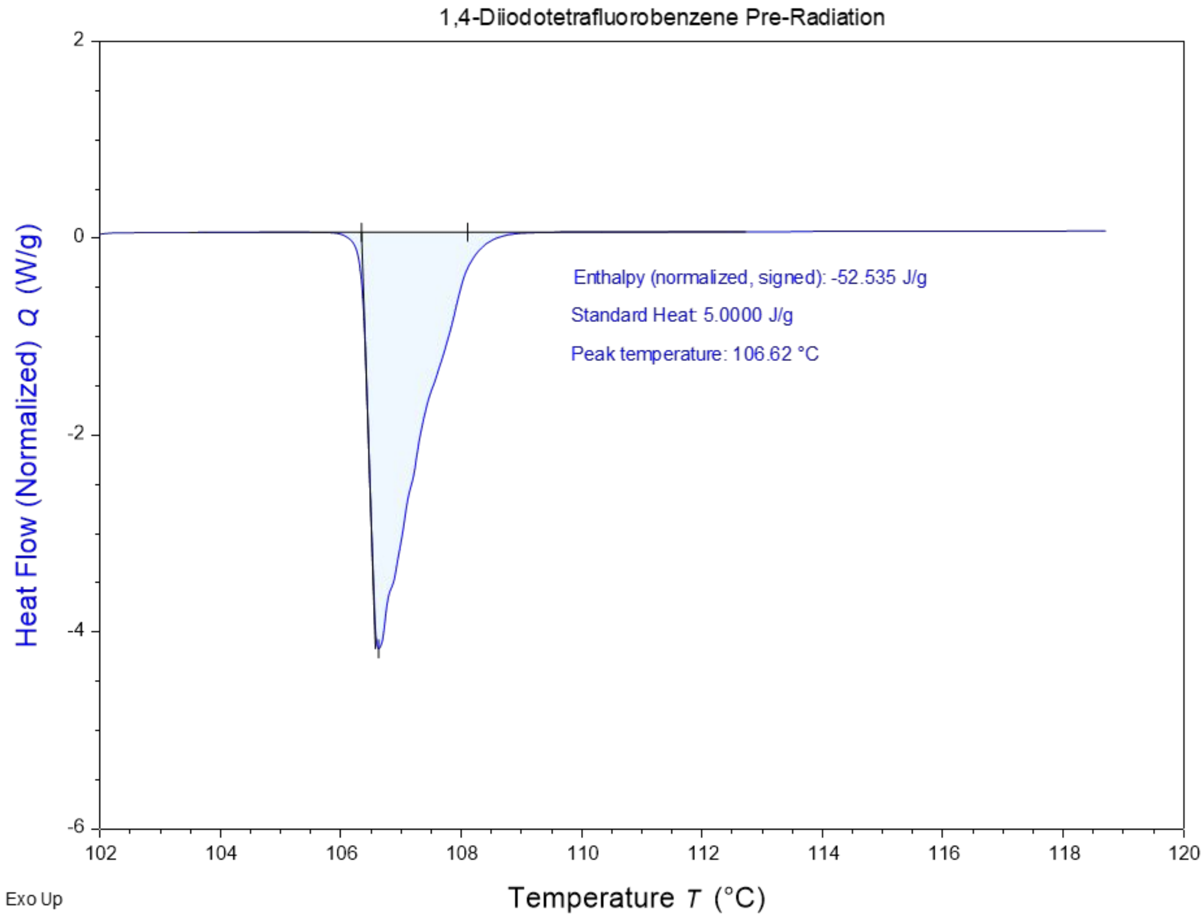

Figure S68. Integrated differential thermogram of **1,4-$C_6I_2F_4$** pre-radiation.

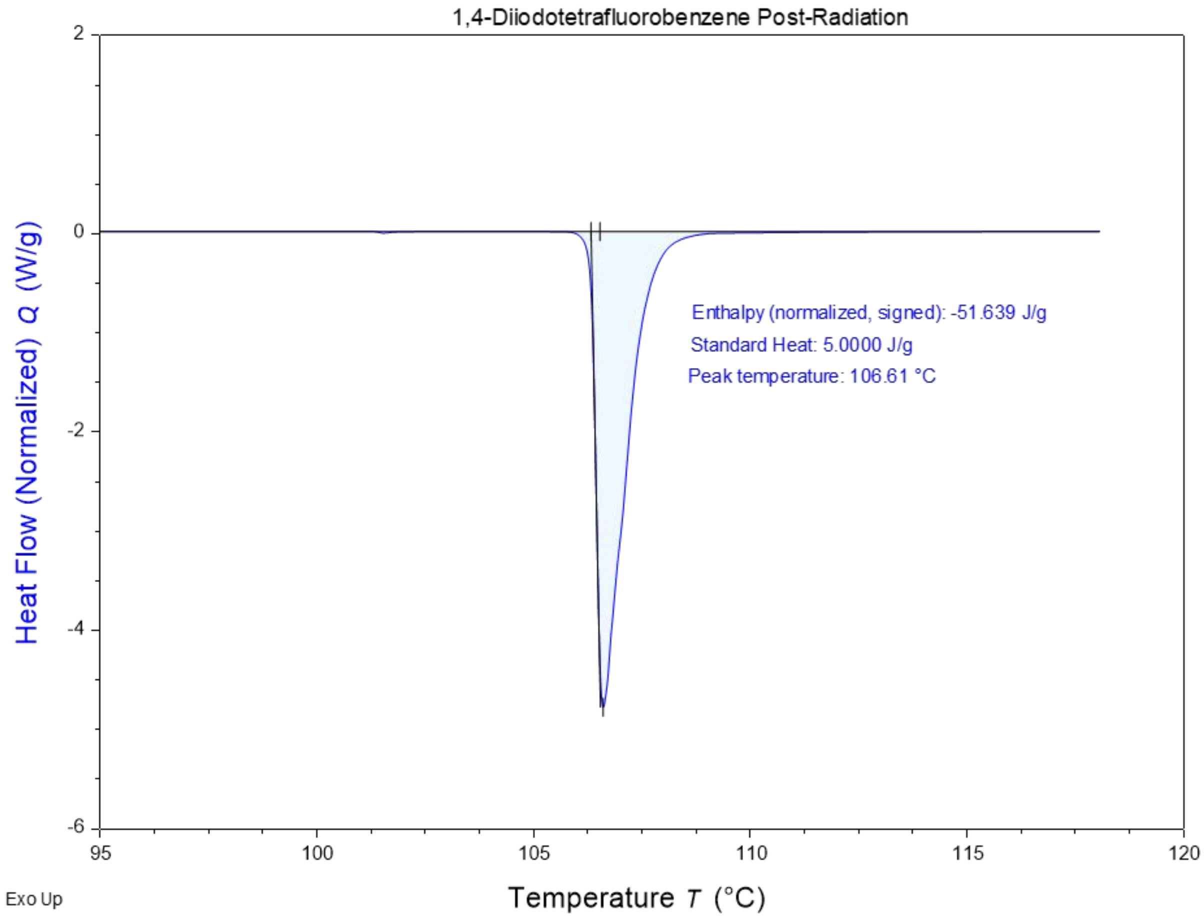

Figure S69. Integrated differential thermogram of **1,4-$C_6I_2F_4$** post-radiation.

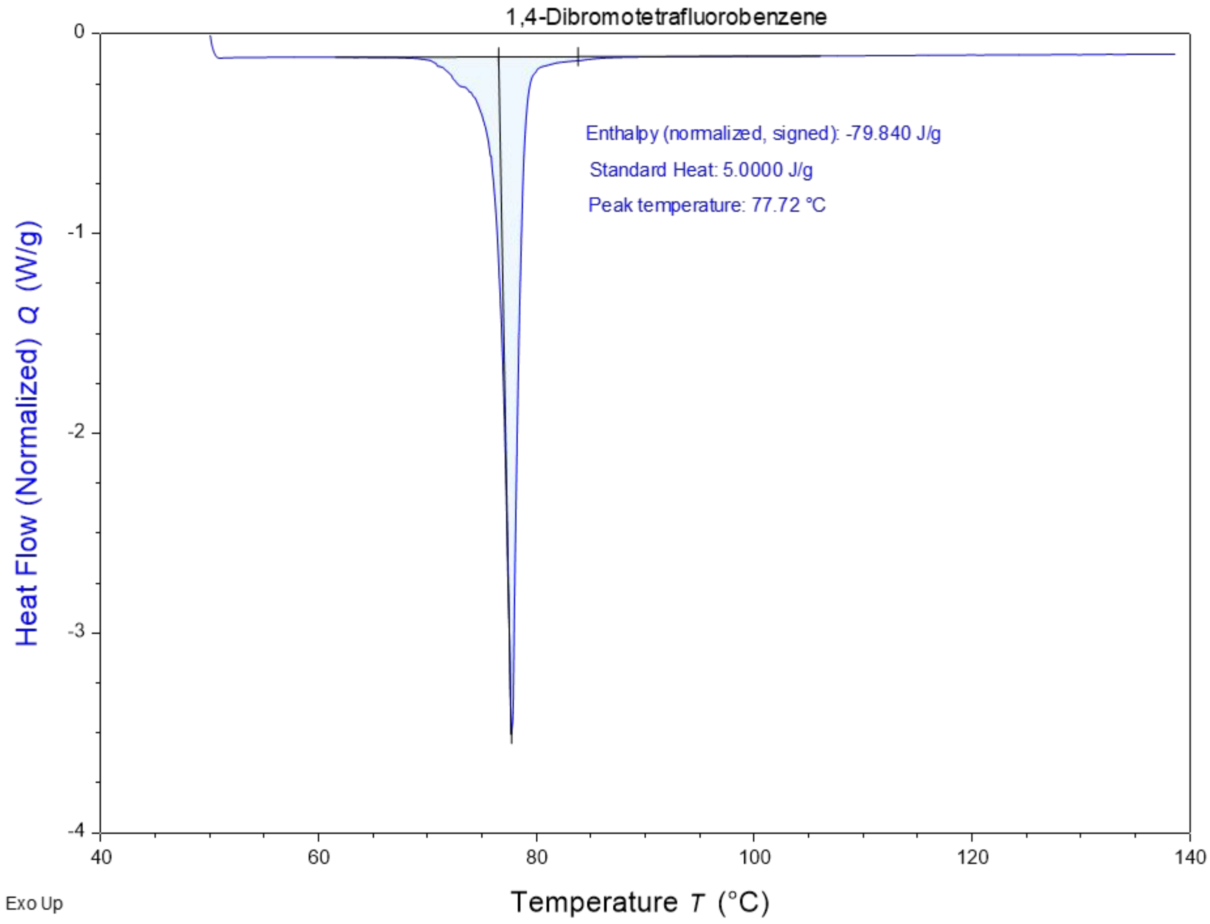

Figure S70. Integrated differential thermogram of **1,4- $C_6Br_2F_4$** pre-radiation.

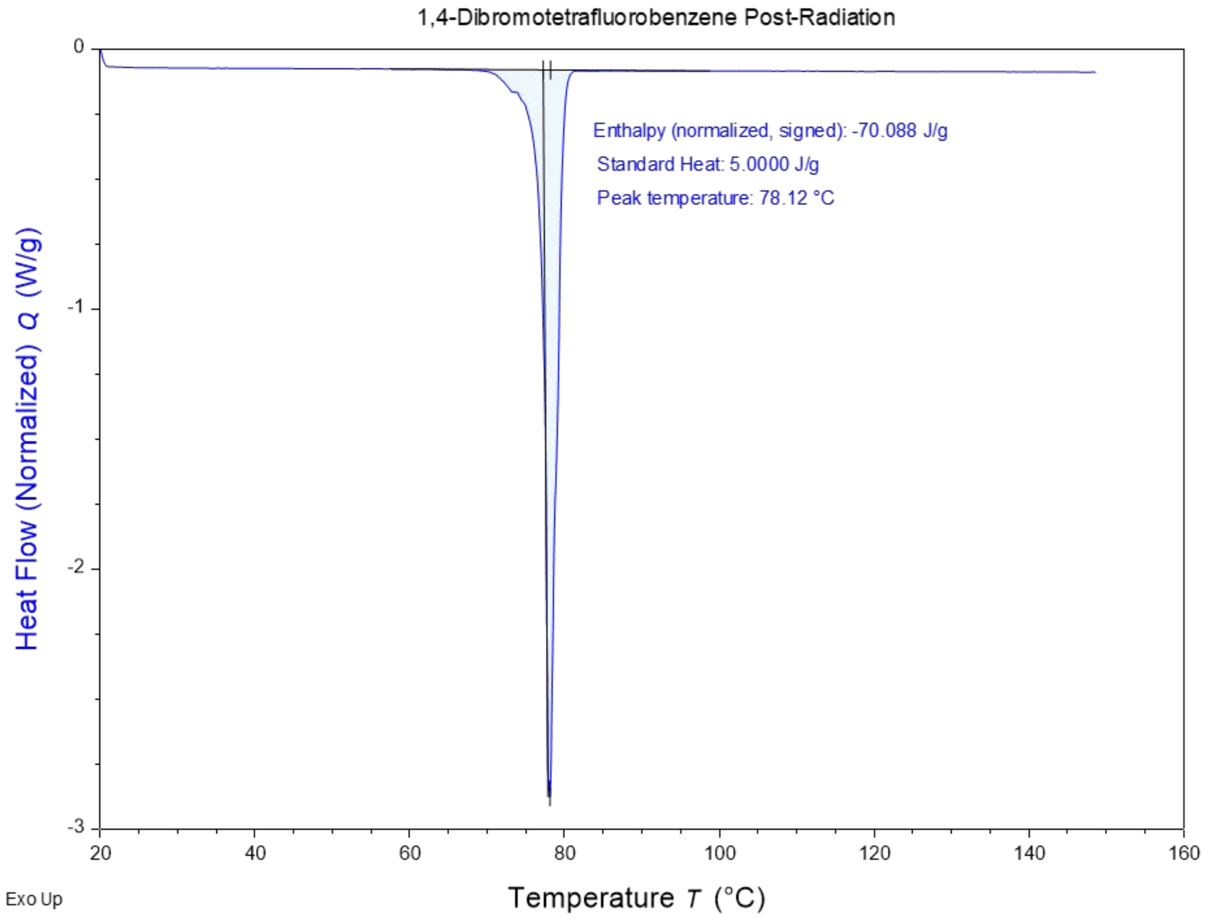

Figure S71. Integrated differential thermogram of **1,4-$C_6Br_2F_4$** post-radiation.

a)

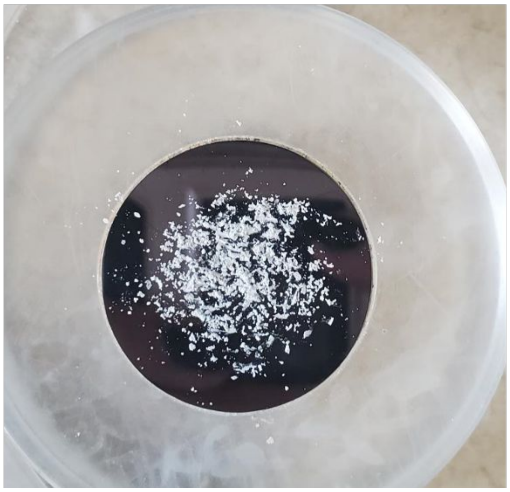

b)

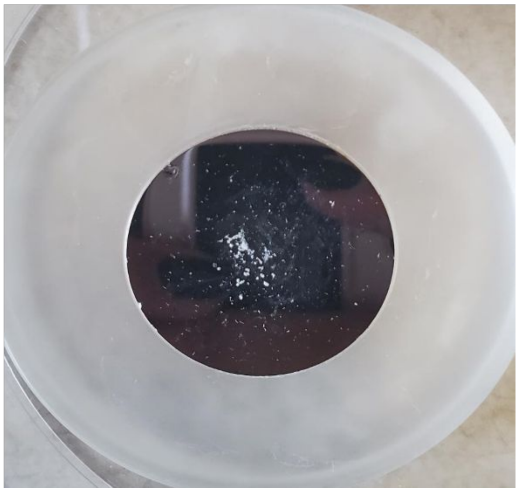

Figure S72. 1,4-$C_6Br_2F_4$ powder post-irradiation with **a)** 0 minutes of air exposure and **b)** 20 minutes of air exposure, showing enhanced sublimation.

References

1. Yadav, R. A.; Singh, I. S., The Raman and Infrared Spectra and Normal Coordinate Analysis for 1,2-Diiodotetrafluorobenzene. The Journal of Raman Spectroscopy, 2005, 14 (5), 353-357.
2. Hanson, G. R.; Jensen, P.; McMurtrie, J.; Rintoul, L.; Micallef, A. S., Halogen Bonding between an Isoindoline Nitroxide and 1,4-Diiodotetrafluorobenzene: New Tools and Tecton for Self-Assembling Organic Spin Systems. Chemistry A European Journal, 2009, **15** (16), 4156-4164.
3. Green, J. H. S.; Harrison, D. J., Vibrational spectra of benzene derivatives—XVIII Dihalogenotetrafluorobenzenes, Spectrochimica Acta Part A: Molecular Spectroscopy, 1977, **33** (2), 193-197.
4. Greaves, S., J.; Griffith, W. P., Vibrational spectra of catechol, catechol-d_2, and -d_6 and the catecholate monoanion. Spectrochimica Acta Part A: Molecular Spectroscopy, 1991, **47** (1), 133-140.
5. Onawole, A. T.; Halim, M. A.; Ullah, N.; Al-Saadi, A. A., Structural, spectroscopic and docking properties of resorcinol, its -OD isotopomer and dianion derivative: a comparative study. Structural Chemistry, 2018, **19**, 403-414.
6. Kubinyi, M.; Billes, F.; Grofcsik, A.; Keresztury, G., Vibrational spectra and normal coordinate analysis of phenol and hydroquinone, Journal of Molecular Structure, 1992, 266, 339-344.
7. Meić, Z.; Güsten, H., Vibrational studies of trans-stilbenes—I. Infrared and Raman spectra of trans-stilbene and deuterated trans-stilbenes, Spectrochimica Acta Part A: Molecular Spectroscopy, 1978, **34** (1), 101-111.
8. Yang, W.; Hulteen, J.; Schatz, G. C.; Van Duyne, R. P., A surface-enhanced hyper-Raman and surface-enhanced Raman scattering study of trans-1,2-bis(4-pyridyl)ethylene adsorbed onto silver film over nanosphere electrodes. Vibrational assignments: Experiment and theory. Journal of Chemical Physics, 1996, **104** (11), 1413-4323.
